# In search of the Goldilocks zone for hybrid speciation

**DOI:** 10.1371/journal.pgen.1007613

**Published:** 2018-09-07

**Authors:** Alexandre Blanckaert, Claudia Bank

**Affiliations:** Instituto Gulbenkian de Ciência, Oeiras, Portugal; University of Michigan, UNITED STATES

## Abstract

Hybridization has recently gained considerable interest both as a unique opportunity for observing speciation mechanisms and as a potential engine for speciation. The latter remains a controversial topic. It was recently hypothesized that the reciprocal sorting of genetic incompatibilities from parental species could result in hybrid speciation, when the hybrid population maintains a mixed combination of the parental incompatibilities that prevents further gene exchange with both parental populations. However, the specifics of the purging/sorting process of multiple incompatibilities have not been examined theoretically. We here investigate the allele-frequency dynamics of an isolated hybrid population that results from a single hybridization event. Using models of two or four loci, we investigate the fate of one or two genetic incompatibilities of the Dobzhansky-Muller type (DMIs). We study how various parameters affect both the sorting/purging of the DMIs and the probability of observing hybrid speciation by reciprocal sorting. We find that the probability of hybrid speciation is strongly dependent on the linkage architecture (i.e. the order and recombination rate between loci along chromosomes), the population size of the hybrid population, and the initial relative contributions of the parental populations to the hybrid population. We identify a Goldilocks zone for specific linkage architectures and intermediate recombination rates, in which hybrid speciation becomes highly probable. Whereas an equal contribution of parental populations to the hybrid population maximizes the hybrid speciation probability in the Goldilocks zone, other linkage architectures yield unintuitive asymmetric maxima. We provide an explanation for this pattern, and discuss our results both with respect to the best conditions for observing hybrid speciation in nature and their implications regarding patterns of introgression in hybrid zones.

## Author summary

Hybridization is observed ubiquitously in nature. Its outcome can range from extinction to the creation of new species. With respect to the latter, the probability of homoploid hybrid speciation, i.e. the formation of a new species as a result of a hybridization event without changes in the ploidy of the organism, is a hotly debated topic. Here, we analyze a minimal model for homoploid hybrid speciation, in which reproductive isolation is achieved by means of (postzygotic) Dobzhansky-Muller incompatibilities. When these postzygotic genetic incompatibilities are resolved in the hybrid population, their reciprocal sorting can result in reproductive isolation from both parental populations, thus creating a hybrid species. We show that, in accordance with the current literature, hybrid speciation tends to be rare. However, specific arrangements of the genes responsible for reproductive isolation can make reciprocal sorting almost unavoidable and thus create barriers to the parental population in an almost deterministic matter. We discuss the implications of these results for hybrid speciation and patterns of introgression in nature.

## Introduction

The role of hybridization in adaptation and speciation is an ongoing contentious question [1–10]. On the one hand, hybridization may serve as a source of genetic variation. Various examples of adaptive introgression have been reported (reviewed in [11]), and it has been argued that hybridization may provide the fuel for adaptive radiations [12]. On the other hand, gene flow between diverging populations may slow down or even reverse speciation either by purging isolating barriers or by one population swamping the other [13–15]. Thus, hybridization may act both as an engine of speciation and a boost to genetic variation, and as a detrimental mechanism that reduces population fitness and promotes extinction. This duality makes hybridization an important subject of study not only from an evolutionary but also a conservation biology point of view.

Hybrid speciation describes a scenario in which hybridization is essential for the formation of a “daughter” species that is isolated from both its parental species. The term “hybrid speciation” covers different scenarios that can be distinguished by the mechanism responsible for the buildup of reproductive isolation. In the case of polyploidization, individuals of the newly formed species possess more than one copy of each parents’ chromosomes. The parents can be of the same species (autopolyploidization, although the hybrid species tends to be outcompeted by the parental diploid [6]), or different ones (allopolyploidization), resulting in a single-step speciation event. In contrast, homoploid hybrid speciation (or recombinational speciation) corresponds to the formation of a hybrid species without a change in the ploidy level. For homoploid hybrid speciation to be possible, two criteria have to be fulfilled. On the one hand, there must be existing genetic isolating barriers between the parental species. On the other hand, these barriers have to be incomplete, such that sufficiently fit F1 hybrids can found the hybrid species. Despite this apparent paradox, numerous empirical cases of homoploid hybrid speciation have recently been reported [16–22]. Whether all of these represent true cases of homoploid hybrid speciation has been subject to debate. This debate has been led mainly around the definition of hybrid speciation and the resulting implications for the reported cases of empirical evidence [9, 10, 23]. However, to our knowledge there exists little work that has evaluated the probability of hybrid speciation theoretically.

Buerkle *et al*. [4] studied a specific case of hybrid speciation via two overlapping parental inversions. Their simulations suggested a rather narrow parameter range in which hybrid speciation is possible, and indicated that (among other restrictions) high fertility of F1 hybrids is necessary to produce a stable hybrid population, which, as a consequence, is only poorly isolated from its parental species. Moreover, Schumer *et al*. [24] studied the conditions for reciprocal sorting of genetic incompatibilities. A single genetic incompatibility can only isolate the hybrid population from one of its parental origins. However, if multiple DMIs exist between two species, in a hybrid population they might be resolved reciprocally with respect to the parental allelic origin, which can result in a hybrid species that is isolated from both parental populations. Proposing this model, Schumer *et al*. [24] demonstrated via simulations that pairs of genetic incompatibilities can trigger hybrid speciation when there is a cost to both the ancestral genotype and the incompatibility-bearing genotypes. Based on a similar model, Comeault [25] recently investigated the impact of adaptive loci linked to genetic incompatibilities on the probability of homoploid hybrid speciation in a stepping-stone model.

Inspired by Schumer’s model, we here provide a detailed analysis of the probability and dynamics of (reciprocal) sorting of classical Dobzhansky-Muller incompatibilities (DMIs [26–28]). A DMI consists of two (individually neutral or beneficial) alleles at different loci that are negatively epistatic, i.e., their combination is deleterious. The Dobzhansky-Muller model is arguably the most widely accepted model to explain the buildup of intrinsic postzygotic isolation in allopatric populations, and many examples of DMIs have been identified empirically [29–36]. Under this premise, such DMIs are expected to be the most prevalent type of genetic incompatibility that can be involved in reciprocal sorting and thus contribute to hybrid speciation. In our model, direct selection on the derived alleles is assumed to be absent or weak as compared with epistasis (in contrast to the fitness landscape in Schumer *et al*. [24]), and thus our model relies exclusively on classical DMIs [37]. We explain the differences between the two models in Supplementary Section S1. Note that we focus only on the genetic mechanisms responsible for hybrid speciation and ignore the potential role of ecological factors [38].

**Fig 1:**
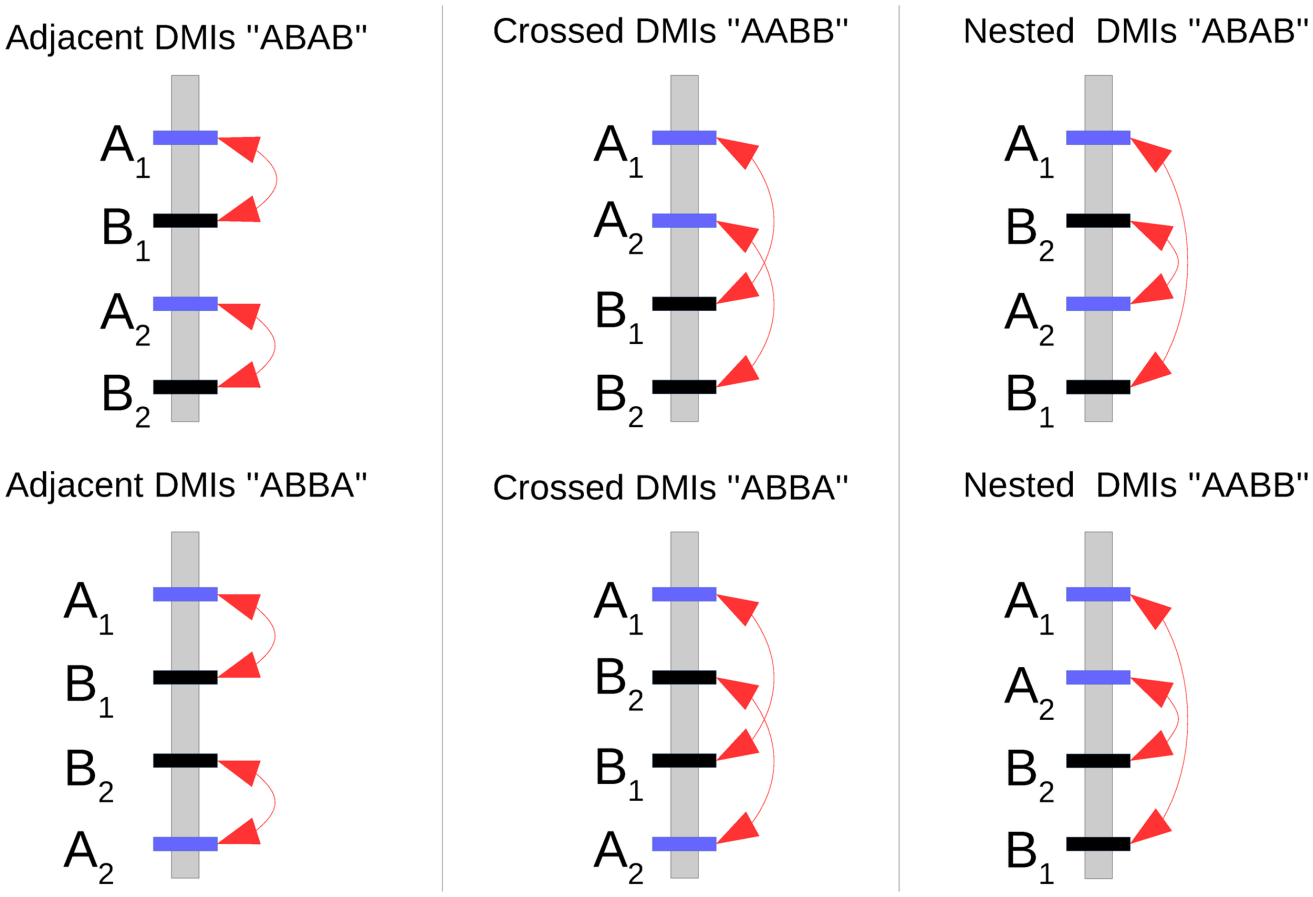
Illustration of all six different linkage architectures possible along a single chromosome. The *A*_*k*_ loci are given in blue and *B*_*k*_ in black. Red arrows show the incompatible interactions. The name of each linkage architecture is derived from the relative arrangement of the two incompatibilities and the order of the *A* and *B* loci.

We identify several parameters that greatly influence the probability of hybrid speciation via DMIs. Specifically, we quantify how the population size, the initial contribution of parental alleles, and the linkage architecture affect the probability of hybrid speciation. As linkage architecture, we define the relative position of and recombination rate between the different loci involved in the hybrid incompatibilities that contribute to the species barriers (see also Fig 1). Consistent with Schumer *et al*. [24], we define hybrid speciation as the successful reciprocal sorting of incompatibilities, independent of the amount of isolation they confer. We discuss both weak and strong isolating barriers and consider recessive and codominant DMIs [39, 40], which differ considerably in their sorting patterns. Our results indicate that the linkage architecture of the DMIs plays an essential role, such that a specific architecture can make hybrid speciation almost unavoidable, whereas exchanging only two loci may make hybrid speciation impossible for otherwise unaltered parameter values. Thus, we identify a Goldilocks zone of hybrid speciation, in which an interplay of population size, linkage architecture and the relative contribution of parental genomes can make hybrid speciation more likely than previously assumed.

## Materials and methods

### Simulations

Simulations are implemented in C++ (available at https://gitlab.com/evoldyn/four-loci) and each run ended when the population was monomorphic.

## Results

### Model

**Fig 2:**
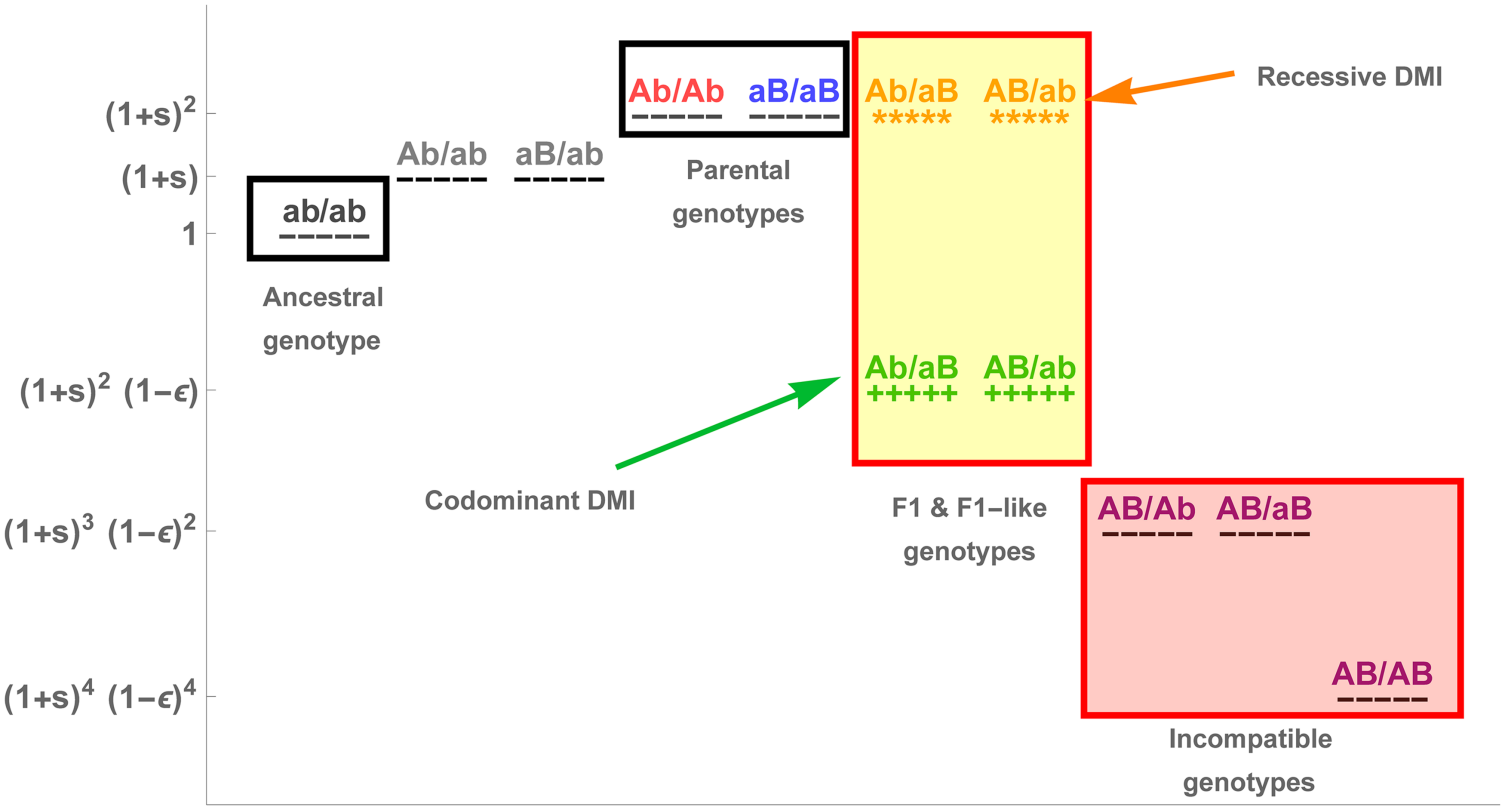
Fitness landscape of the 16 genotypes in the two-locus model, highlighting the effect of dominance of the incompatibility on the fitness of F1 hybrids. For simplicity, we illustrate the case of *α* = *β* = *s*, where *s* is the selective advantage of a derived allele in the ancestral background. Note that there are only 10 genotypes represented here, as we do not distinguish the parental origins of each haplotype.

We study the evolution of a single population of constant size *N* in discrete generations. We model four diallelic loci, *A*_1_, *A*_2_, *B*_1_, *B*_2_. At each locus, the derived allele is named after the locus, and the ancestral allele is named after its corresponding lower-case letter. Note that we do not detail here the two-locus model as it is fully included in the four-locus model. It can be obtained by keeping only loci *A*_1_ and *B*_1_. Derived alleles at the different loci are under direct selection (soft selection), with *α*_*k*_ the (direct) fitness advantage of allele *A*_*k*_ over *a*_*k*_, and *β*_*k*_ the fitness advantage of allele *B*_*k*_ over *b*_*k*_. Selection happens in the diploid phase of the life cycle. In addition, negative epistasis, *ϵ*_*k*_, which determines the strength of hybrid incompatibility, occurs in a pairwise fashion between the derived *A*_*k*_ and *B*_*k*_ alleles (with *k* ∈ {1, 2}). Dominance affects only the epistatic interactions, whereas the direct fitness effects are assumed to be multiplicative throughout the whole manuscript. We focus mainly on two cases of dominance of the epistatic interactions, which were proven representative of the general pattern [40]: a recessive scenario and a codominant scenario, illustrated in Fig 2. We introduce $\phi_{k}^{n}$ as a mathematical placeholder used to distinguish between the recessive and codominant scenario at the DMI *k*, with *n* the number of pairs of incompatible alleles in a genotype. Note that *n* = 1, *n* = 2 and *n* = 4 correspond to the *H*_0_, *H*_1_ and *H*_2_ incompatibilities in Turelli & Orr [39]. Therefore, for a codominant DMI, $\phi_{k}^{n}=1\;\forall n\in \left\{0,1,2,4\right\}$ while for a recessive DMI, the effect of epistasis is masked for the double heterozygote genotype, i.e. $\phi_{k}^{1}=0$ while ∀*n* ∈ {0, 2, 4}, $\phi_{k}^{n}=1$.

The population is initially composed of two single genotypes, since it is assumed to result from a one-time secondary contact between two monomorphic parental populations 1 and 2; *i*_*p*_ denotes the contribution of the parental population 1 to the newly formed hybrid population. We assume that parental population 1 is fixed for the *A*_1_*b*_1_*A*_2_*b*_2_/*A*_1_*b*_1_*A*_2_*b*_2_ genotype and parental population 2 for *a*_1_*B*_1_*a*_2_*B*_2_/*a*_1_*B*_1_*a*_2_*B*_2_. The fitness of a genotype composed of haplotypes *i* and *j* is given by:

$$\begin{array}{c} \omega^{ij}=\prod_{k=1}^{2}{\left(1+\alpha_{k}\right)}^{X_{k}^{i}+X_{k}^{j}}{\left(1+\beta_{k}\right)}^{Y_{k}^{i}+Y_{k}^{j}}{\left(1+\phi_{k}^{\left(X_{k}^{i}+X_{k}^{j}\right)\left(Y_{k}^{i}+Y_{k}^{j}\right)}\cdot \epsilon_{k}\right)}^{\left(X_{k}^{i}+X_{k}^{j}\right)\left(Y_{k}^{i}+Y_{k}^{j}\right)}, \end{array}$$ (1)

where $X_{k}^{i}$ is the number of alleles *A*_*k*_ in haplotype *i* and $Y_{k}^{i}$ the number of alleles *B*_*k*_ in haplotype *i*. The full fitness landscape is illustrated in S1 Fig.

Mating is random. We assume that the parents generate an infinite pool of gametes from which zygotes are formed through multinomial sampling *M*(2*N*, *p*_1_, …, *p*_16_). Note that the deterministic case (i.e., in the absence of drift, *N* → ∞) can be obtained by skipping the multinomial sampling step during zygote formation.

As introduced above, hybrid speciation is defined as the fixation of a haplotype that is incompatible with both parental haplotypes, see Table 1. Indeed, if an individual homozygous for the *A*_1_*b*_1_*a*_2_*B*_2_ haplotype is backcrossed with an individual from, e.g., parental population 1, then the second DMI is expressed either in the F1 generation (codominant case) or in the F2 generation (recessive case). Similarly, introduction of an *A*_1_*b*_1_*a*_2_*B*_2_/*A*_1_*b*_1_*a*_2_*B*_2_ individual in the parental population 2 leads to the expression of the first DMI. This definition corresponds to an early stage speciation mechanism, leading to a hybrid population that is only partially isolated from both parental populations. Note that full isolation is impossible in this setting, as barriers responsible for full reproductive isolation would also prevent the formation of the hybrid population in the first place.

**Table 1:** Classification of possible haplotypes for the “Adjacent ABAB” linkage architecture.

Ancestral haplotype	*a*_1_*b*_1_*a*_2_*b*_2_
Parental pop. 1 haplotype	*A*_1_*b*_1_*A*_2_*b*_2_
Parental pop. 2 haplotype	*a*_1_*B*_1_*a*_2_*B*_2_
Hybrid haplotypes	*A*_1_*b*_1_*a*_2_*B*_2_ or *a*_1_*B*_1_*A*_2_*b*_2_
“Epistasis-free” F2 haplotype	*A*_1_*b*_1_*a*_2_*b*_2_ or *a*_1_*B*_1_*a*_2_*b*_2_ or *a*_1_*b*_1_*A*_2_*b*_2_ or *a*_1_*b*_1_*a*_2_*B*_2_
1^st^ incompatibility haplotypes	*A*_1_*B*_1_*a*_2_*b*_2_ or *A*_1_*B*_1_*A*_2_*b*_2_ or *A*_1_*B*_1_*a*_2_*B*_2_
2^nd^ incompatibility haplotypes	*a*_1_*b*_1_*A*_2_*B*_2_ or *a*_1_*B*_1_*A*_2_*B*_2_ or *A*_1_*b*_1_*A*_2_*B*_2_
Double-incompatibilities haplotype	*A*_1_*B*_1_*A*_2_*B*_2_

We consider all possible linkage architectures formed by the two DMIs; they are illustrated in Fig 1. There are 6 different ways to arrange the 4 loci along a single chromosome (assuming the chromosome does not have an orientation). The two DMIs can be “Adjacent”, “Crossed”, or “Nested” (Fig 1). The recombination rate between adjacent loci X and Y is given by 0 ≤ *r*_*XY*_ ≤ 0.5. The recombination rate between non-adjacent loci X and Y, separated by a single locus W, is given by *r*_*XY*_ = *r*_*XW*_(1 − *r*_*WY*_) + *r*_*WY*_(1 − *r*_*XW*_). If the four loci are spread across multiple chromosomes, this represents a special case of the single-chromosome scenario presented above, in which one or more recombination rates are set to 0.5. If not otherwise specified, we assume that all loci are located on different chromosomes, i.e. *r*_*XY*_ = 0.5.

### Resolution of a single DMI

In the first part, we focus on the resolution of a single DMI following the formation of the hybrid population. We define a DMI as “resolved” when either allele *A* or *B* is lost, such that there is no genetic conflict left in the population. With a single DMI, hybrid speciation according to our definition is impossible, because one of the negatively interacting partners *A* and *B* in the DMI will invariably be lost (see also below). Thus, maintaining a genetic barrier to both parental species is impossible. Nevertheless, the study of a single incompatibility pair is necessary to understand which properties can be extrapolated to multiple incompatibilities. We characterize the resolution of the genetic conflict resulting from the contact between two diverged populations by quantifying the probability of fixation of the different haplotypes, the time of resolution of the DMI (i.e., the time until at least one of the incompatible alleles is lost) and the time to fixation of a single haplotype. In this section, we only focus on the loci *A*_1_ and *B*_1_ and drop the indices as they do not carry any information.

#### Dynamics following secondary contact

**Fig 3:**
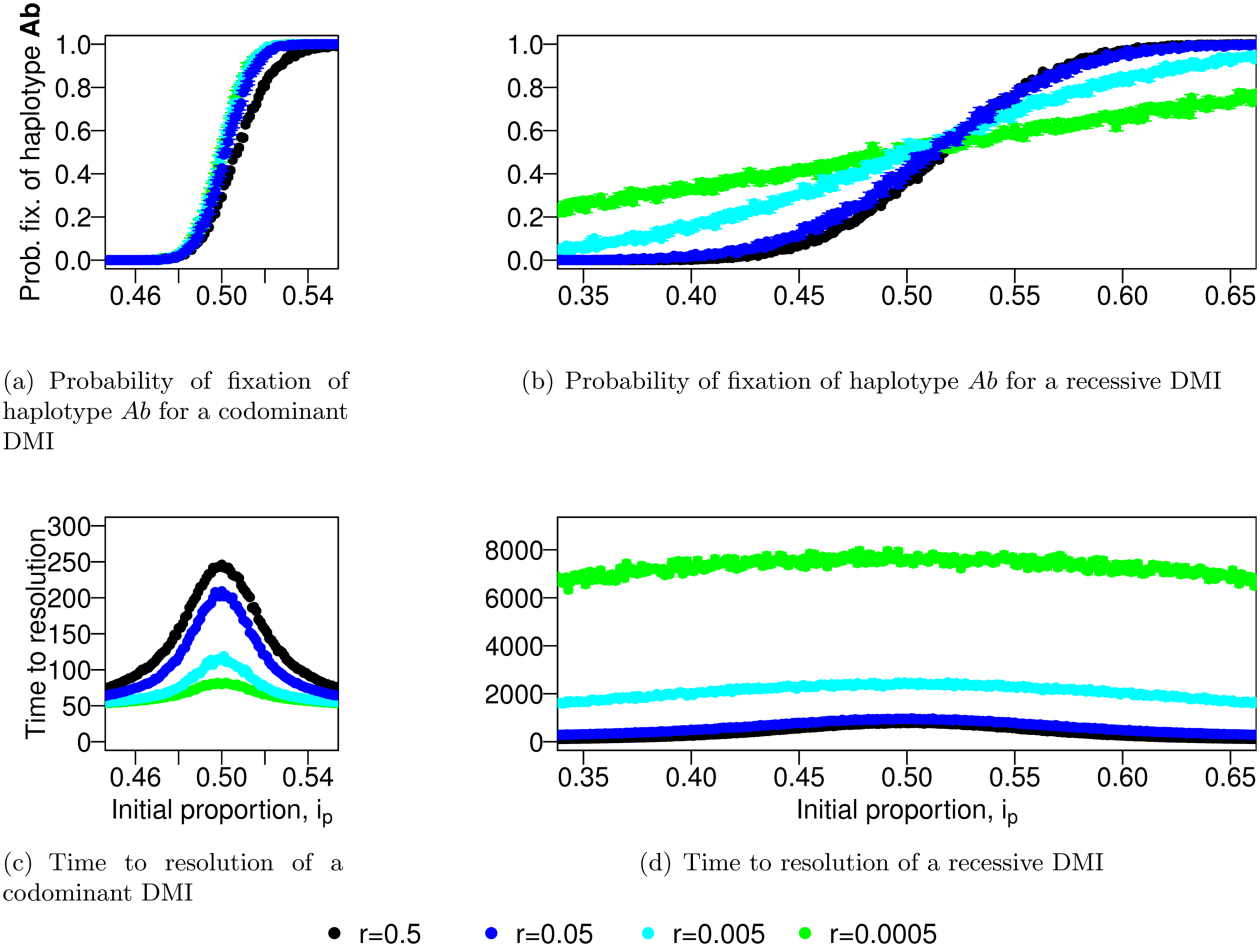
Recombination slows down the resolution of a codominant DMI whereas it speeds DMI resolution up for a recessive one. We represent the probability of fixation of the *Ab* haplotype (panels (a,b)) for different recombination rates and different dominance schemes (codominant (a,c), recessive (b,d)). In panels (c,d), we illustrate the time to resolve the genetic conflict (i.e., either allele *A* or *B* is lost). Each value is obtained from 1000 independent simulations. Note the much larger y-axis scale (x30) in panel (d). Parameters used: *α* = *β* = 0.001, *ϵ* = −0.2, *N* = 5000.

In a single randomly mating population such as the hybrid population we consider here, a DMI cannot be maintained unless directional selection is strong as compared with the epistatic effect of the incompatibility [40]. This is because the formation of hybrid individuals initially leads to selection against both derived haplotypes. These haplotypes suffer from the incompatibility, either directly by forming an unfit hybrid genotype or indirectly through the production of unfit offspring. In contrast, the ancestral haplotype has an advantage as soon as it appears, and rises in frequency, because it only forms compatible genotypes and produces compatible offspring (if the proportion of incompatible *AB* haplotypes in the population remains low). As soon as the ancestral haplotype becomes frequent or either of the derived haplotypes becomes rare, this marginal advantage disappears, and the ancestral type will either be swamped by the more frequent derived type (in the case of direct selection acting on the derived alleles, i.e., if *α*, *β* > 0), or segregate neutrally (if *α*, *β* = 0). The incompatibility is usually resolved in favor of the more frequent derived allele (if they have similar fitness). Thus, one main determining factor is the initial frequency ratio between the two derived alleles (Supplementary Section S2.1). Both direct selection and codominance of the incompatibility reduce the impact of genetic drift (i.e., the outcome converges to the deterministic case). Indeed, once the DMI is resolved, selection increases the probability of fixation of a single derived allele [41, 42]. The codominance of the incompatibility shortens the time required to resolve the DMI (Fig 3), and therefore reduces the time spent at low frequencies, where loss of the derived alleles because of drift is a likely outcome (see Supplementary Section S2.2).

#### Recombination has opposite effects under different dominance schemes

Recombination has a dual impact on the outcome of a hybridization event, depending on the dominance of the DMI, as illustrated in Fig 3 for haplotype *Ab*. Recombination breaks the association between the alleles of the parental haplotype and therefore leads to the formation of both the incompatible haplotype *AB* and the ancestral haplotype *ab*. On the one hand, this allows the expression of the incompatibility through the formation of the *AB* haplotype, which leads to faster sorting of the derived alleles. On the other hand, building a genotype with the ancestral haplotype protects both parental haplotypes from suffering from the genetic incompatibility, which leads to slower sorting of the derived alleles. The balance between these two effects is different between a recessive and a codominant DMI, which results in an opposite behavior, as illustrated in the following.

In the recessive case, recombination is necessary for the expression of the incompatibility, because only production of *AB* haplotypes unmasks the epistatic interaction. The faster these haplotypes are produced, the stronger (epistatic) selection acts, which leads to a faster resolution of the DMI. An increase in recombination therefore always accelerates the resolution of a recessive DMI. This reduces the time the derived alleles spend at low frequency, which makes them less susceptible to being lost through genetic drift. This, in turn, reduces the probability that the ancestral haplotype becomes fixed.

In the codominant case, the incompatibility is already expressed in the F1 generation. Recombination is not necessary to express the incompatibility and therefore slows down the resolution of the DMI, as the ancestral haplotype prevents the effective purging of the parental haplotypes through the formation of *ab*/*Ab* or *ab*/*AB* individuals. In this situation, both derived alleles are driven to lower frequencies than in the recessive model, which makes them more likely to be both lost through genetic drift, resulting in the fixation of the ancestral haplotype.

### Resolution of two DMIs and hybrid speciation

Expanding from what we learned above, we now consider what happens when two incompatibilities exist between the parental populations. In contrast to the case of a single DMI, a new evolutionary outcome, namely hybrid speciation, becomes feasible with more than one DMI. As “hybrid speciation”, we denote the reciprocal sorting of the two DMIs, i.e. fixation of either alleles *A*_1_ and *B*_2_ or *A*_2_ and *B*_1_. Such a hybrid population will then carry isolating barriers to both parental populations.

#### Isolation of the hybrid population by reciprocal sorting of two DMIs

**Fig 4:**
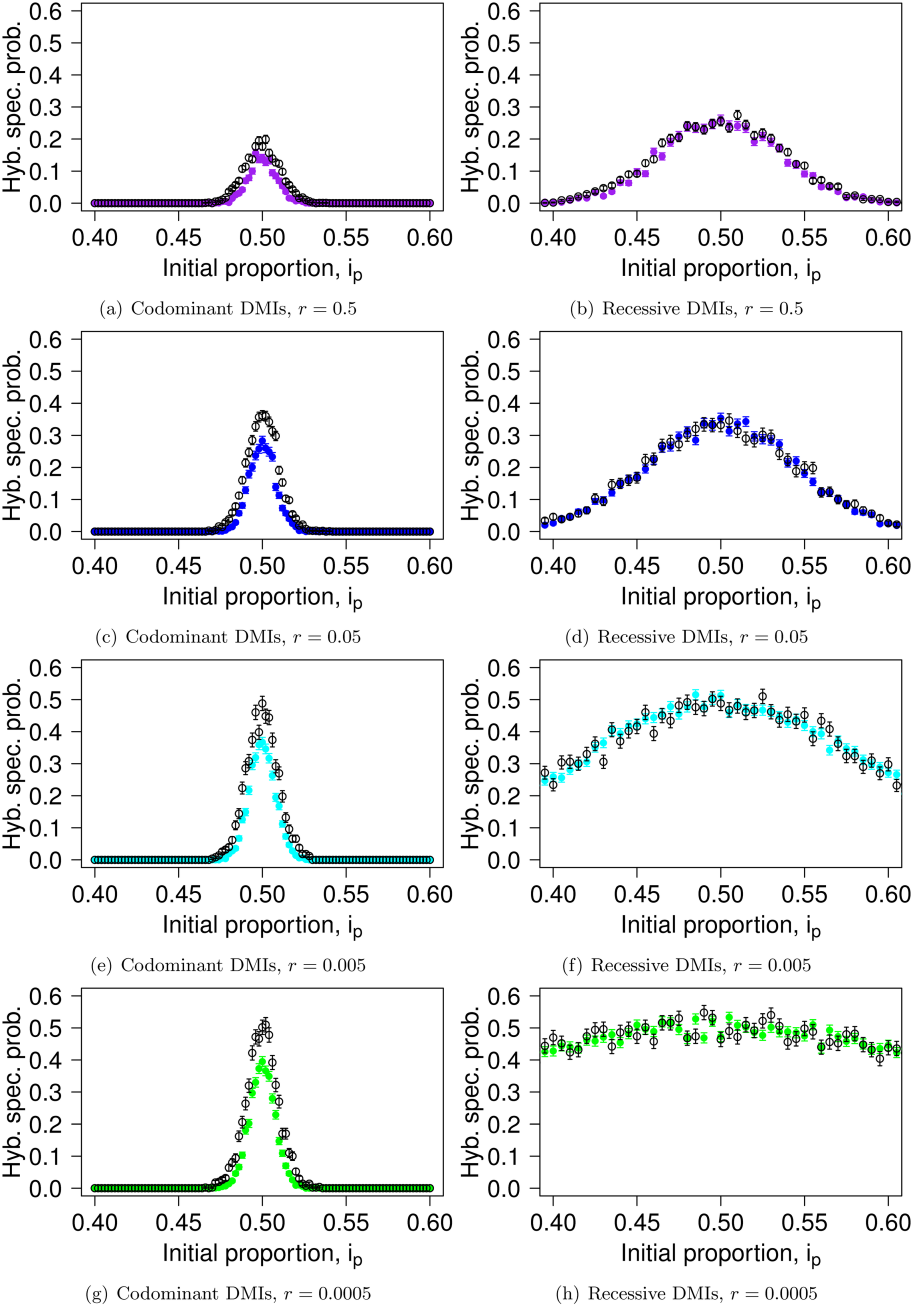
Hybrid speciation probability for codominant (panels (a,c,e,g)) and recessive (panels (b,d,f,h)) DMIs. The colored dots correspond to the probability of hybrid speciation for two DMIs situated on different chromosomes (*r*_23_ = 0.5). The recombination rate between the interacting loci is indicated below each panel (*r*_12_ = *r*_34_ = *r*): panels (a,b), *r* = 0.5; panels (c,d), *r* = 0.05; panels (e,f), *r* = 0.005; panels (g,h), *r* = 0.0005. The black dots correspond to the predicted hybrid speciation probability based on the resolution of a single DMI. The fast resolution of the codominant DMIs leads to a correlation between their fate, which makes hybrid speciation less likely than the independent expectation predicts. Parameters used are *α*_*i*_ = *β*_*i*_ = 0.001, *N* = 5000, *ϵ* = −0.2. Each dot is obtained from 1000 replicates.

Given the observed shape of the fixation probability of a derived allele in the case of a single DMI as a function of the initial contribution of both parental populations (Fig 3), hybrid speciation should be observable only around symmetric contact, and this condition should be more stringent for codominant incompatibilities than recessive ones. In Fig 4, we test this expectation by comparing the probability of hybrid speciation for two DMIs that are located on separate chromosomes (with *A*_1_ and *B*_1_ on the first chromosome and *A*_2_ and *B*_2_ on the second one; colored dots in Fig 4) with the expected probability of resolving two independent single DMIs for opposite derived alleles (e.g. first DMI resolved towards allele *A* and the second one for allele *B*; black dots). In the recessive case, the prediction for independent DMIs matches the hybrid speciation probability. In the codominant case, the independent expectation overestimates the probability of hybrid speciation. This can be explained by the faster resolution of the DMIs in the codominant model, which, even in the case of free recombination, leaves insufficient time for the two DMIs to become uncoupled and independently resolved, as the *A*_1_ and *A*_2_ loci start in maximum linkage disequilibrium. In the codominant case, this effect is amplified at low recombination rates as, in that case, the resolution of the DMIs happens even faster (S3 Fig), therefore preserving more of the initial linkage disequilibrium. This leads to a positive correlation between the fixation of the different *A*_*i*_ alleles (as well as *B*_*j*_ alleles), S2 Fig. In the recessive case, the resolution of the two DMIs remains independent as it takes much longer to resolve any single recessive DMI.

S3 Fig illustrates the mean time to resolve both DMIs in opposite directions conditioned on the outcome being hybrid speciation. Recombination has the same effect on the resolution of two DMIs as it did for a single one: it accelerates the resolution of recessive DMIs and slows down the resolution of codominant DMIs. However, the average resolution time is not affected by the initial proportion of the parental species; only trajectories that resolve slowly, which ensure that the initial linkage disequilibrium has been broken, can contribute to hybrid speciation, and we are conditioning on this outcome.

Due to the complexity of the dynamics, the possibility to obtain analytical results was very limited. However, we were able to obtain a condition for the (deterministic) local stability of the hybrid species with respect to back mutation or single migration events (see Supplementary Section S4). Most notably, we find that the condition for local stability of the monomorphic equilibrium that contains the reciprocally sorted haplotypes is independent of the dominance of the incompatibilities.

#### The linkage architecture determines which alleles survive

**Fig 5:**
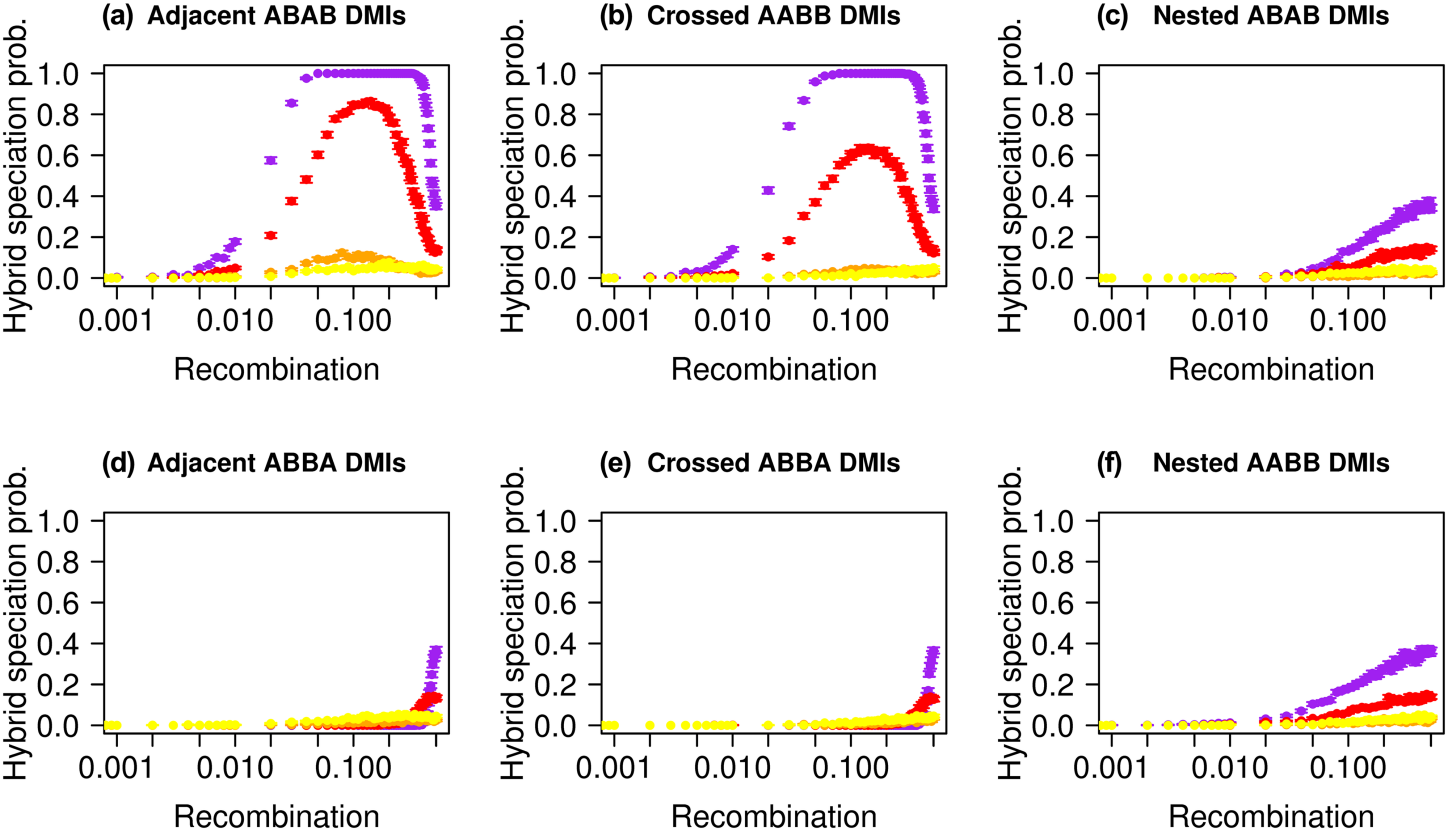
Hybrid speciation probability is a nonlinear function of recombination. We consider that all four loci have the same selective advantage (*α*_*k*_ = *β*_*j*_ = 0.001) and are equidistant along a single chromosome. The hybrid speciation probability is plotted for different population sizes: yellow corresponds to *N* = 50, orange to *N* = 500, red to *N* = 5000 and purple to *N* = 50000. Epistasis (*ϵ* = −0.2) is here codominant but we obtain qualitatively similar results for recessive incompatibilities, see S10 Fig. The contribution of both parental populations is symmetric (*i*_*p*_ = 0.5). Each panel corresponds to a different linkage architecture: (a) “Adjacent ABAB”; (b) “Crossed AABB”; (c) “Nested ABAB”; (d) “Adjacent ABBA”; (e) “Crossed ABBA”; (f) “Nested AABB”.

Fig 5 illustrates the effect of recombination and the linkage architecture on hybrid speciation, when all loci are on the same chromosome (as opposed to one DMI per chromosome, as illustrated in Fig 4). As mentioned above, for codominant DMIs, recombination, on the one hand, allows the formation of the hybrid haplotype and helps to reduce the initial linkage disequilibrium. On the other hand, it slows down the resolution of the DMI through the formation of compatible haplotypes. Depending on the balance between these two effects, recombination impacts the probability of hybrid speciation differently.

**Fig 6:**
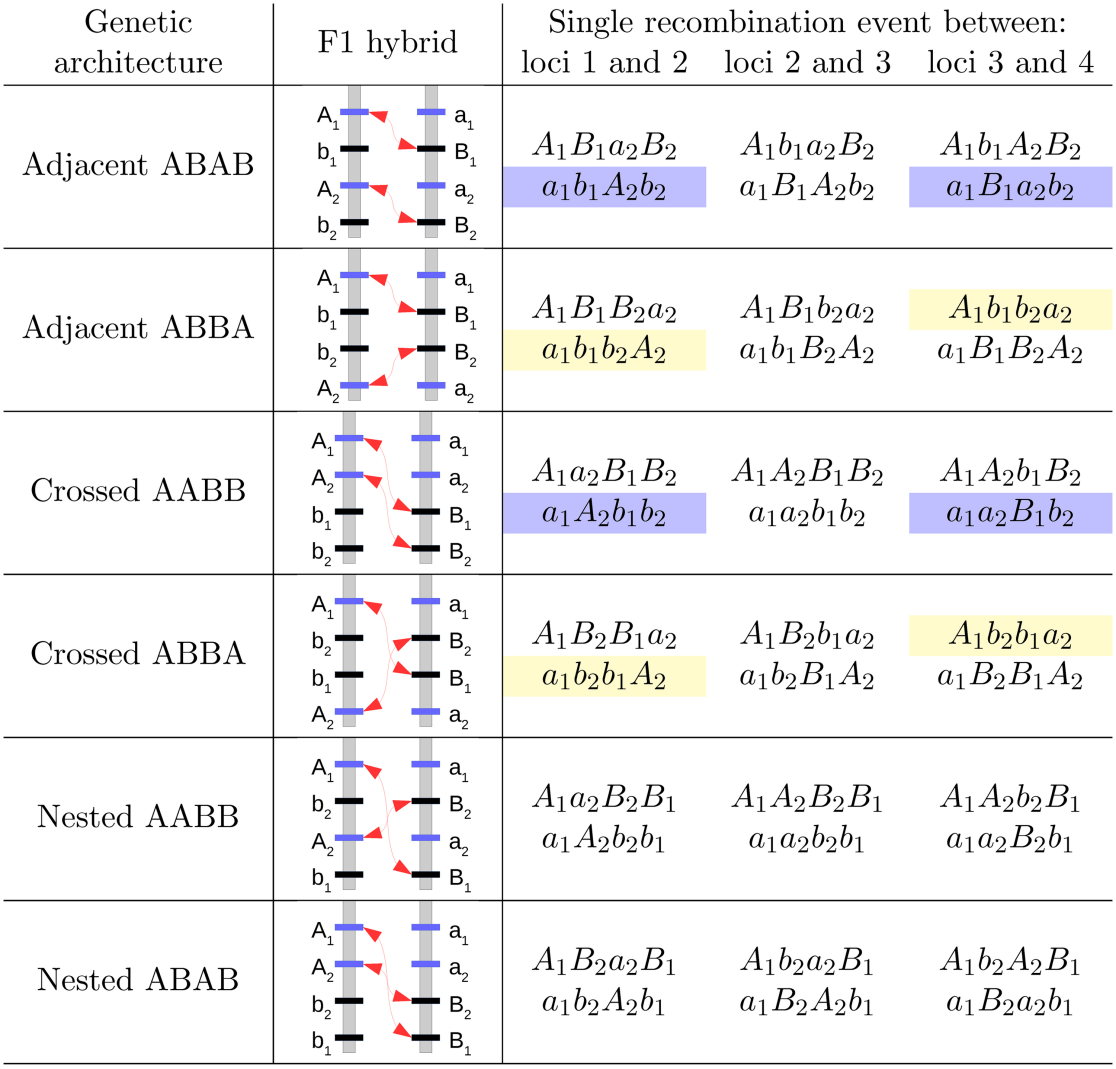
Haplotypes produced in the F2 breakdown, assuming a single recombination event, explain how different linkage architectures leads to different outcomes for the same loci. By identifying the relatively “epistasis-free” haplotype formed, one can infer whether hybrid speciation may be a likely outcome. In blue, we highlight these “epistasis-free” haplotypes that are important for hybrid speciation and in yellow those that are important for fixation of the parental haplotype from population 1.

Assuming a symmetric contact, we observe that two of the linkage architectures, the “Adjacent ABAB” and “Crossed ABAB” architectures exhibit a non-monotonic behavior with maximum hybrid speciation probability for intermediate recombination rates. These linkage architectures have in common that the loci *A*_1_ and *B*_2_ are located at the ends of the chromosomal region that contains the four focal loci. Hybrid speciation becomes almost certain for large population sizes and indeed corresponds to the deterministic outcome (i.e. in the absence of drift) for these two linkage architectures. More precisely, we observe a local maximum of the hybrid speciation probability for recombination rates around *r* = 0.1. The “Adjacent ABAB” and “Crossed ABAB” architectures, which show this behavior, are characterized by a higher marginal fitness of the *A*_2_ and *B*_1_ alleles compared to the other alleles in the deterministic case, which promotes hybrid speciation. For all other architectures either *A*_1_ and *A*_2_ or *A*_2_ and *B*_2_ have the highest marginal fitness. The higher marginal fitness stems from the production of the *a*_1_*B*_1_*a*_2_*b*_2_ and *a*_1_*b*_1_*A*_2_*b*_2_ haplotypes in the F2 generation (for the “Adjacent ABAB” architecture) that are relatively free of epistasis. The outcomes of a single recombination event per genome for all 6 architectures are given in Fig 6 and illustrate how the “Adjacent ABAB” and “Crossed ABAB” architectures stand out in the production of the haplotypes that are needed for hybrid speciation. Importantly, recombination is necessary to generate these haplotypes, but too much recombination will cancel their advantage. Indeed, for *r* = 0.5, all haplotypes are produced at the same frequency in the absence of selection. The dual effect of recombination leads therefore to the observed maximum in the hybrid speciation probability for intermediate recombination rates. When the DMIs are located on two different chromosomes (as in Fig 4), this effect does not appear. Indeed, while recombination still breaks linkage disequilibrium, it no longer generates the relatively “epistasis-free” haplotype and therefore leads to a monotonous increase in the hybrid speciation probability with increasing recombination rate. This behavior, specific to the “Adjacent ABAB” and “Crossed AABB” linkage architectures, is observed both for codominant and recessive DMIs.

As illustrated in S10 Fig, the recessive case is qualitatively similar to the codominant one. We recover the distinctive pattern between linkage architectures, where the “Adjacent ABAB” and “Crossed AABB” architectures are more likely to generate hybrid speciation for intermediate recombination rates. However, for the “Adjacent ABBA” and “Crossed ABBA” linkage architectures, the recessive case differs from the codominant by the existence of two local maxima for the hybrid speciation probability as a function of recombination. These two architectures are characterized by an indirect selective advantage of one of the two parental haplotypes over the other, as the partially derived haplotypes *A*_1_*b*_1_*b*_2_*a*_2_ and *a*_1_*b*_1_*b*_2_*A*_2_ are more likely to be formed than their counterparts (*a*_1_*B*_1_*b*_2_*a*_2_ or *a*_1_*b*_1_*B*_2_*a*_2_, see Fig 6), which leads to a slightly higher marginal fitness of the *A*_1_*b*_1_*b*_2_*A*_2_ haplotype compared to *a*_1_*B*_1_*B*_2_*a*_2_. The first maximum is obtained at large intermediate recombination rates; it corresponds to the one observed for codominant DMIs. However, a second one can be observed at lower recombination rate if the population size is large enough. It results from a subtle balance between drift, recombination and selection, which we explain in detail in Supplementary Section S3.1.

The hybrid speciation probability for codominant versus recessive DMIs differs significantly when considering lethal incompatibilities (see Supplementary Section S3.1). Hybrid speciation becomes impossible for codominant DMIs because no viable hybrids can be produced. This is not the case for recessive incompatibilities, as they can partially escape the strong selection against hybrids. In fact, due to the masking effect provided in F1 and F1-like genotypes, we observe an almost indistinguishable pattern for deleterious (*ϵ* = −0.2) and lethal (*ϵ* = −0.99) recessive DMIs, see S14 Fig. Similarly, the time to hybrid speciation is similar between the deleterious and lethal recessive cases, see Supplementary Section S3.3.

Fig 5 also illustrates the impact of the population size on the outcome. In general, a larger population size results in a higher probability of hybrid speciation. This is especially true when the deterministic outcome corresponds to hybrid speciation (i.e., for the “Adjacent ABAB” and “Crossed AABB” architectures). In large populations, derived alleles are less likely to be lost during the reciprocal sorting of the genetic incompatibilities. The main exception to this rule exists when the deterministic outcome is the fixation of one parental haplotype. In that case, an intermediate population size will maximize the likelihood of hybrid speciation, as illustrated in Fig 5 for the “Adjacent ABBA” and “Crossed ABBA” architectures (see also S10 Fig). This intermediate value corresponds to a balance between a strong drift regime in which the ancestral and “epistasis free” haplotypes are most likely to fix, and the deterministic regime in which the *A*_1_*b*_1_*b*_2_*A*_2_ parental haplotype fixes.

#### Symmetric contact is not always the best condition for hybrid speciation

**Fig 7:**
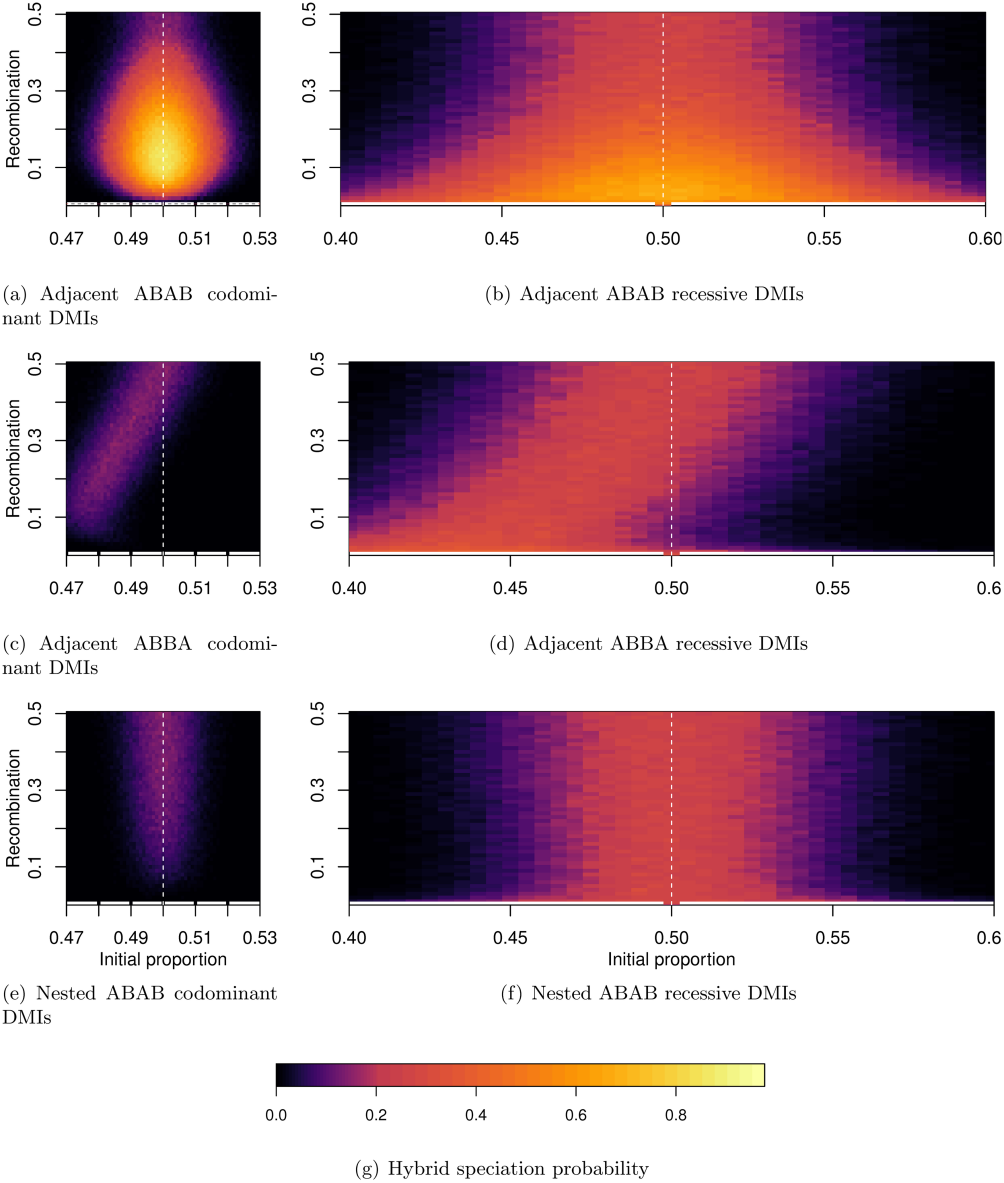
Probability of hybrid speciation for both recessive and codominant DMIs as a function of the recombination rate between the different loci (they are all equidistant) and the initial contribution of both parental species. Different linkage architectures generate an unexpected pattern: for the “Adjacent ABAB” architecture, we observe a Goldilocks zone for hybrid speciation; for the “Adjacent ABBA”, the probability of hybrid speciation is no longer symmetric along the *i*_*p*_ = 0.5 axis (white dashed line). Each panel corresponds to a given linkage architecture and a dominance scheme: (a) “Adjacent ABAB”, codominant DMIs; (b) “Adjacent ABAB”, recessive DMIs; (c) “Adjacent ABBA”, codominant DMIs; (d) “Adjacent ABBA” recessive DMIs; (e) “Nested ABAB”, codominant DMIs; (f) “Nested ABAB” recessive DMIs.

Fig 5 was obtained for *i*_*p*_ = 0.5, i.e. when both parental populations contribute equally to the hybrid population. It corresponds to the case that is the most frequently investigated in models. Fig 7 illustrates what happens when we release this assumption. From the single-DMI dynamics, one would expect a decrease in the hybrid speciation probability as illustrated in Fig 5. This is not always true. Depending on the linkage architecture, the probability of hybrid speciation may be higher for asymmetric contributions from the parental populations. This phenomenon is also observed for intermediate recombination rates; thus, only a consideration of dominance scheme, recombination rate, and symmetry together allows for an accurate statement on the hybrid speciation probability (see Fig 7). Fig 6 provides us with an explanation for the observed pattern: for intermediate recombination rate (*r* ≈ 1/3), there is on average one recombination event per haplotype per generation. For the two architectures concerned (“Adjacent ABBA” and “Crossed ABBA”), in this scenario and with perfect symmetry, both alleles *A*_1_ and *A*_2_ have a marginal fitness that is slightly higher than alleles *B*_1_ and *B*_2_ (S4 Fig), which leads to the fixation of the parental haplotype *A*_1_*b*_1_*b*_2_*A*_2_ in the deterministic case. Therefore, a lower initial frequency of these alleles at the initial contact balances this selective advantage, which results in larger hybrid speciation probabilities than under symmetry. This behavior is only observed for the two architectures discussed above (“Adjacent ABBA” and “Crossed ABBA”). For the other architectures, the two derived alleles that have a slight indirect selective advantage are *A*_2_ and *B*_1_ for “Adjacent ABAB” and “Crossed AABB” (which correspond to the cases of high probabilities of hybrid speciation) or *A*_2_ and *B*_2_ for the two “Nested” architectures. In both cases, since the symmetry between the A and B alleles is respected, hybrid speciation is most likely at *i*_*p*_ = 0.5.

## Discussion

We here characterized the purging process of single and multiple DMIs upon formation of an isolated hybrid population. Specifically, we quantified the effects of the linkage architecture and the dominance of the epistatic interactions on the reciprocal sorting of incompatibilities, which has been proposed as a mechanism to induce homoploid hybrid speciation. We found that for the exact same set of loci, their order along the chromosome can increase the probability of observing hybrid speciation from unlikely to almost certain. We demonstrate that the main determinant of this pattern is which haplotypes are formed during the F2 breakdown. For the linkage architectures that promote hybrid speciation, there exists a Goldilocks zone in which an intermediate recombination rate maximizes the hybrid speciation probability. In addition, we show that symmetric contact of incompatible loci that are under equal selection pressure does not always generate the highest probability of hybrid speciation, and that this result cannot be predicted from the study of independent DMIs. Finally, hybrid speciation with lethal codominant DMIs is impossible, whereas for recessive DMIs, in which the F1 generation does not suffer a fitness disadvantage, reciprocal sorting is similarly probable with intermediate and strong epistasis.

### Linkage architecture as major determinant of hybrid speciation probability

Abbott *et al*. [7] recently emphasized that “an important challenge in studies of hybrid speciation is to ask whether there is an ‘optimal’ genetic distance for homoploid hybrid speciation [3, 43].” Although Abbott *et al*. [7] were arguably referring to the degree of divergence and thus, to the number and strength of isolating barriers that have established between two species, our study adds an additional important factor to their list: the linkage architecture of the isolating barriers, and the recombination rate between them. Specifically, our results demonstrate that intermediate recombination rates and specific linkage architectures maximize the probability of hybrid speciation.

We can speculate whether the presence of multiple DMIs should increase the probability of hybrid speciation. Based on our results, we believe that it should depend on the nature of the incompatibilities: additional recessive DMIs should increase the hybrid speciation probability while more codominant DMIs should reduce it. Firstly, we showed that for codominant DMIs, the fixation of derived alleles of the same parental population is correlated (S2 Fig), which hinders their reciprocal sorting even if they are located on different chromosomes. Hence, recombination is not sufficient to decrease the initially existing linkage disequilibrium. Adding additional codominant incompatibility loci will result in stronger selection against F1 individuals which strengthens the correlation of parental alleles, and reduces the probability of reciprocal sorting. Secondly, we have shown that for lethal codominant DMIs (S14 Fig), hybrid speciation is impossible. Extrapolating from these two observations, we propose that this effect will outpace the increase in hybrid speciation probability due to having more chances to have at least one pair of reciprocal sorting.

On the other hand, in the recessive model, F1 hybrids do not suffer a fitness cost, and the fixation of derived alleles from the same parental origin is uncorrelated. In addition, stronger epistasis does not affect the probability of hybrid speciation in the recessive model (S10 and S14 Figs). Thus, having more than two recessive DMIs should increase the chances that at least two are reciprocally sorted, which is sufficient for hybrid speciation according to our definition.

Our results can be discussed in the context of parapatric speciation and the role of genomic islands of divergence [44, 45]. According to the respective theory [46], during the speciation process and in presence of gene flow, islands of divergence are formed around the first genes involved in reproductive isolation. These genes will reduce gene flow locally around them, which favors the accumulation of weakly locally adapted mutations in their vicinity, as well as incompatible genes, reinforcing and extending islands of divergence. For hybrid speciation according to our model, the existence of such islands implies that many derived alleles *A*_*k*_ may be found together on the same island, as locally adaptive loci tend to be rearranged in clusters [47]. In such cases the linkage disequilibrium between the different *A*_*k*_ alleles will be harder to break, which makes reciprocal sorting less probable. From the perspective of this model, hybrid speciation should therefore be most likely early during speciation, when no strong islands of divergence have formed yet. We further note that our model considers a single hybridization event without any further gene exchange with both parental populations, which resembles the colonization of a new environment. Continuous gene flow between populations, which is often a key feature of studies of genomic islands of speciation, should further reduce the probability of hybrid speciation [25], because migration creates selective pressure against the hybrid haplotypes.

### The probability of hybrid speciation and reciprocal sorting in nature

Our results imply that although specific linkage architectures may indeed induce hybrid speciation with high probability, it remains on average unlikely. Other factors that we neglect in the minimal model presented here (such as polymorphic or complex DMIs, and ecological factors) may alter the hybrid speciation probability. However, our results are consistent with the scarcity of putative cases of homoploid hybrid speciation observed in nature [16–22]. Recently, Runemark *et al*. [48] reported that the Italian sparrow hybrid species resulted from multiple occurrences of hybridization between the Spanish and House sparrow along the Mediterranean Sea, which is in concordance with our observation of a Goldilocks zone. The observed resolution of all hybridization events towards a single mitochondrial origin (i.e. all Italian sparrows possess the mitochondrial DNA of House sparrows) indicates that either the mitochondria played an important role with respect to the sorting of the incompatibilities, or that there is an asymmetry in the formation of the different hybrid populations, in which Spanish sparrow males mate with House sparrow females.

### The nature of genetic incompatibilities

Both theoretical considerations and empirical evidence suggest that most DMIs should be recessive [29, 49]. However, any kind of dominance pattern of the epistatic interactions can in theory exist [50]. Here, we showed that single codominant DMIs are resolved much faster than recessive ones. Therefore, when non-equilibrium populations are sampled, the excess of recessive incompatibilities may not necessarily reflect the true proportion of recessive incompatibilities but rather a sampling bias.

### Genetic isolation of the hybrid population

Homoploid hybrid speciation relies on an apparent paradox: it requires that the existing genetic barriers are strong enough for the hybrid population to be isolated from both parental populations, while the same barrier needs to be weak enough to allow the formation of the hybrid population in the first place. We find that the reciprocal sorting of the two DMIs leads to a significant barrier for codominant DMIs (see S5 Fig) regardless of the genetic and linkage architecture of the incompatible loci. On the contrary, recessive DMIs only reduce the introgression probability significantly when the incompatibilities are lethal and the incompatible loci are far apart from each other. The measure for reproductive isolation we used here, the probability of introgression of an unlinked neutral allele, is conservative compared to measures at the barrier loci themselves [40, 51, 52]. However, S5 Fig illustrates that early isolation of a hybrid population from its parental sources is possible for both dominance schemes. Nevertheless, in contrast to the polyploidisation scenario, complete reproductive isolation by means of homoploid hybrid speciation cannot be an immediate outcome but may happen by building on the initial reproductive isolation that reciprocal sorting confers.

### The time to hybrid speciation

We specifically evaluated the timing of two events during the process of hybrid speciation. The first is when the genetic conflict caused by the DMIs is resolved by losing one of the interacting partners in each incompatibility pair; we call this the resolution time. From this point onwards, there are no epistatic interactions left in the population and the evolution of the remaining genotypes is dictated only by genetic drift and direct selection. The second event is the time at which all polymorphism is lost; conditioned on reciprocal sorting we call this the time to hybrid speciation. Only then, the isolating barrier to the parental populations is fixed in the population.

We find that initially, codominant DMIs are resolved faster, which leads to a short resolution time. This process is even faster with low recombination, because the lack of recombination decreases the probability to break the epistatically interacting haplotypes. (This also leads to a low probability of hybrid speciation for codominant DMIs with low recombination.) However, after resolution of the DMI, the derived alleles still need to become fixed in order for hybrid speciation to occur. This process is usually faster for recessive DMIs and compensates for the slower initial sorting, such that both codominant and recessive DMIs lead to similar times to hybrid speciation (see S15 and S16 Figs).

Schumer *et al*. [24] emphasized that hybrid speciation can sometimes happen quickly. Somewhat contradictory, we find that the time to hybrid speciation (scaled by population size) increases with smaller population sizes, and that there is relatively little variation in the times to hybrid speciation for constant population size (S15 and S16 Figs). For example, for a population of size N = 50, the time to hybrid speciation is around 5N = 250 generations (i.e., longer than the average fixation time of a neutral allele) independent of the linkage architecture of the DMI. For a population of size N = 50000, it is only around 0.1N = 5000 generations. That is because both epistatic and direct selection act more efficiently in large populations.

### Population size and selection

In our model, we consider populations of constant size. Relaxing this assumption (i.e. switching from soft selection to hard selection), one would expect hybrid speciation to be less likely for at least two reasons. First, selection against the different derived alleles in the early purging phase is stronger; indeed, with soft selection the effect of a mutation is weighted by the mean fitness of the population. Therefore, in maladapted populations, the effect of deleterious mutations is slightly dampened. Second, the expected decrease in population size that is associated with the purging phase increases the impact of drift, which means that reciprocal sorting is less likely even with favorable linkage architectures (as selection is not strong enough to counteract its effect). Lastly, even if the DMIs are resolved in opposite directions, the different derived alleles will be at low frequency when their interacting partner is lost, and therefore more likely to be lost by drift subsequently. Overall, this implies that hybrid speciation via reciprocal sorting is on average less likely than illustrated here. Furthermore, one-time secondary contact between two diverged populations (or species) is usually geographically restricted, and therefore tends to happen for small populations. However, this apparent rarity of hybrid speciation can be counteracted by the frequent formation of hybrid populations; this could suggest that the reported cases of homoploid speciation may simply reflect a geographical distribution conducive to the formation and isolation of hybrid population. The Italian Sparrow seems to fit this scenario remarkably well [48].

The search for signs of hybrid speciation in very large populations, for example yeast, will be an exciting avenue for hybrid speciation research in the future (see e.g., [53]). That is because, firstly, multiple DMIs, both inter- and intraspecific, have been identified in yeast [30, 54, 55]. In addition, yeast can be easily maintained in the lab and lends itself to powerful experimental approaches. The large population size and the possibility to create and maintain multiple replicates are two strong advantages of experimental-evolution approaches to potentially quantify the reciprocal sorting of DMIs. Note that a case of natural homoploid hybrid speciation in yeast was recently reported, in which reproductive isolation was indeed attributed to postzygotic mechanisms [56].

One remarkable genetic incompatibility in yeast that could serve as a contributor to hybrid speciation was identified by Bui *et al*. [57]. Intriguingly, the phenotype of the incompatibility is an increased mutation rate, which could provide the fuel for adaptation to a new environment upon hybridization [58]. Intrinsic incompatibilities are usually deleterious, however in this specific case they could give a temporary advantage to the hybrid population. Following the formation of a hybrid population, the incompatible haplotype could spread in the population while the hybrid population is adapting to new (harsh) conditions. Such a mechanism could therefore be powerful in a colonization scenario. In the initial stage, the mutator phenotype might increase the possibility of recruiting adaptive mutations. Sorting of the incompatibility happens only at a later stage, which results in a synergy of forming genetic isolating barriers from the parental source populations while delaying the incompatibility-sorting phase until the population has adapted to its environment.

## Conclusion

The probability of hybrid speciation is subject to continuing debate [9, 10, 24]. The reciprocal sorting of parental incompatibilities has been proposed as one credible mechanism to achieve hybrid speciation. Our work legitimates the existing disagreement by demonstrating that the hybrid speciation probability via reciprocal parental incompatibility sorting is highly variable and dependent on the linkage architecture and the dominance type of the involved incompatibilities. Specifically, the linkage architecture determines not only whether hybrid speciation is achievable or not, but also whether equal or unequal initial proportions of the parental populations are favorable for hybrid speciation. In addition, we show that across all studied scenarios, intermediate recombination rates maximize the likelihood of reciprocal sorting; i.e., interactions on the same chromosome are favorable for hybrid speciation. Altogether, we arrive at the prediction that in nature, hybrid speciation via reciprocal sorting of incompatibilities should indeed be rare; at the same time however, it can become almost deterministic (and, thus, repeatable) under specific genetic and demographic circumstances. Such circumstances could be met in microorganisms such as yeast, which have large population sizes and potential for repeated hybridization.

## Supporting information

S1 Fig Fitness landscapes for the codominant and recessive models. Here, we assume the “Adjacent ABAB” architecture and drop the indices to improve readability of the figure. The white dots indicate the position of the parental genotypes and the black dots the position of the hybrid speciation haplotypes. We use the “default” set of parameters: *α*_*i*_ = *β*_*i*_ = 0.001, *ϵ* = −0.2. The fitness advantage of the parental genotypes as compared to the ancestral genotype, chosen here as 0.001^4^ ≈ 0.004, is too small to be visible in the illustration. The arrangement of the haplotypes is arbitrary.
(TIF)

S2 Fig Correlation between fixation of allele *A*_1_ and allele *A*_2_ for codominant (left) and recessive (right) DMIs. Each incompatibility pair is located on a different chromosome. Colors indicate recombination rates between the *A*_*k*_ and *B*_*k*_ loci involved in the DMIs: *r* = 0.5 in black, *r* = 0.1 in purple, *r* = 0.05 in blue and *r* = 0.005 in cyan. Other parameters used are: *α*_*i*_ = *β*_*j*_ = 0.001, *ϵ* = −0.2, *N* = 5000.
(TIF)

S3 Fig Time to resolution of both DMIs conditioning on hybrid speciation. Parameters used here are: *α*_*i*_ = *β*_*j*_ = 0.001, *ϵ* = −0.2, *N* = 5000. For any initial frequency, we performed 1000 simulations and then extracted those simulations that resulted in hybrid speciation.
(TIF)

S4 Fig Marginal fitness of the four derived alleles in the deterministic model (no drift) for the first 100 generations. For the “Adjacent ABAB” and “Crossed AABB” architectures, alleles *A*_2_ and *B*_1_ have a marginal fitness advantage over *A*_1_ and *B*_2_. For the “Adjacent ABBA” and “Crossed ABBA” architectures, alleles *A*_1_ and *A*_2_ have a marginal fitness advantage over *B*_1_ and *B*_2_. Lastly, for the “Nested ABAB” and “Nested AABB” architectures, alleles *A*_2_ and *B*_2_ have a marginal fitness advantage over *A*_1_ and *B*_1_. All DMIs represented here are codominant. Both parental populations contributed equally to the hybrid population, *i*_*p*_ = 0.5. All loci are equidistant with a recombination rate between adjacent loci of *r* = 0.2. Others parameters used are: *α*_*i*_ = *β*_*j*_ = 0.001, *ϵ* = −0.2.
(TIF)

S5 FigRelative fixation probability of an unlinked neutral marker introduced by a parental individual migrating into the monomorphic hybrid population.This probability is displayed relative to the fixation probability of a similar neutral marker that appears in a hybrid individual, *p*_*n*_ = 1/5000. The x-axis corresponds to the recombination rate between the different incompatible loci; the neutral marker is always located on a different chromosome. We estimated the fixation probabilities over 10^6^ replicates. Black lines indicate when the fixation probability is significantly distinct from *p*_*n*_ = 1/5000: any data point below the solid lines is significantly different under a Bonferroni correction (i.e. $\frac{p}{p_{n}}<0.725$ or $\frac{p}{p_{n}}>1.305$). Dashed lines correspond to 95% confidence intervals without Bonferroni correction and provide a visual guide.(TIF)

S6 Fig Illustration of the fitness landscapes for a single DMI in our model and an “adaptive” DMI from [24], S1 Fig. To facilitate the comparison, we use the notation of [24], as presented in Table S1 Table. Parametrization was chosen such that the fitness differences between parental and ancestral genotypes and the strength of the incompatibility for the homozygote incompatible ABAB are the same.
(TIF)

S7 Fig Probability of fixation of allele *A* following formation of the hybrid population. In one case (red dots), haplotype *Ab* is introduced at proportion *i*_*p*_ and *aB* at proportion 1 − *i*_*p*_. To demonstrate that the frequency-dependent effect is dependent on the relative proportion of the *A* and *B* allele, in the other case (black dots), both haplotypes *Ab* and *aB* are introduced at proportion *i*_*q*_ and the ancestral haplotype *ab* is introduced at proportion 1 − 2*i*_*q*_.
(TIF)

S8 Fig Dominance affects the probability of recovering the ancestral haplotype in a hybrid population. Panel (a) shows the probability of recovering the ancestral haplotype *ab* in an isolated hybrid population for the two dominance schemes both for neutral (*α* = *β* = 0) and slightly advantageous mutations (*α* = *β* = 0.001). Panel (b) tracks the marginal fitness of the *ab* haplotype in the deterministic model, for an (almost) symmetric contact (solid lines) and an asymmetric proportion of the parental genomes (dashed lines). Blue and red dots correspond to the data from panel (a). Due to the masking effect of recessivity, the marginal fitness of *ab* is always lower in the recessive case than in the codominant case. Panels (c) and (d) correspond to subsets of panel (b) and illustrate at which point the order of probabilities is reversed for symmetric (panel (d)) and asymmetric contact (panel (c)). Order inversion means that from this time point onwards, masking no longer provides an advantage to the derived alleles. The longer the derived alleles are masked by recessivity, the more likely the ancestral haplotype will fix while both derived alleles are present at an equally low frequency, and therefore susceptible to be lost through drift. Other parameters used are: *N* = 5000, *ϵ* = −0.2 and *α* = *β* = 0.001 for panels (b-d). In panel (a), each data point is obtained from 2000 simulations.
(TIF)

S9 Fig Probability of recovering the ancestral haplotype increases with the time it takes to resolve the DMIs. Colors represent different recombination rates between the *A* and *B* loci. Each dot is the result of 1000 simulations. Parameters used are: *α* = *β* = 0.001 and *N* = 5000.
(TIF)

S10 Fig Hybrid speciation probability for recessive DMIs as a function of recombination for the six possible linkage architectures. We represent this probability for four different population sizes: *N* = 50 in yellow, *N* = 500 in orange, *N* = 5000 in red and *N* = 50000 in purple. Other parameters are *α*_*k*_ = *β*_*j*_ = 0.001, *ϵ* = −0.2 and *i*_*p*_ = 0.5 (i.e. the contribution of both parental populations is symmetric). This figure corresponds to Fig 5 for codominant DMIs.
(TIF)

S11 Fig Proportions of possible evolutionary outcomes for the “Crossed ABBA” architecture. The x-axis corresponds to the recombination rate and each panel shows a different population size. To better illustrate the underlying mechanisms, we represent both hybrid haplotypes separately with *A*_1_*B*_2_*b*_1_*a*_2_ in green and *a*_1_*b*_2_*B*_1_*A*_2_ in yellow.
(TIF)

S12 Fig Proportions of possible evolutionary outcomes for the “Adjacent ABBA” architecture. The x-axis corresponds to the recombination rate and each panel shows a different population size. To better illustrate the underlying mechanisms, we represent both hybrid haplotypes separately with *A*_1_*B*_2_*b*_1_*a*_2_ in green and *a*_1_*b*_2_*B*_1_*A*_2_ in yellow.
(TIF)

S13 Fig Hybrid speciation probability as a function of the population size for recessive (top) and codominant (bottom) DMIs. We focus on the two architectures that displayed a second local maximum of hybrid speciation probability for low recombination rate, here *r* = 0.005. Each color corresponds to a different evolutionary outcome.
(TIF)

S14 Fig Hybrid speciation probability for (quasi-)lethal DMIs as a function of recombination. We consider both codominant and recessive DMIs and two population sizes. Hybrid speciation does not occur for codominant DMIs regardless of population size. For recessive DMIs, the hybrid speciation probability is qualitatively identical to the less deleterious case, see S10 Fig with the blue dots here matching the red dots in S10 Fig and the red diamonds the purple dots in S10 Fig. Parameters used are: *α* = *β* = 0.001, *ϵ* = −0.99.
(TIF)

S15 Fig Time to hybrid speciation is relatively unaffected by the dominance of the epistatic interaction, the strength of the epistasis or the linkage architecture (here: Adjacent ABAB, Adjacent ABBA, Crossed ABAB). Each row of panels represents a different linkage architecture, for which the time to hybrid speciation is shown for codominant (left), recessive (middle), and recessive lethal DMIs (right). We show the average time to hybrid speciation, i.e. to fixation of one of the two hybrid haplotypes, scaled by the size of the population as a function of the recombination rate. Colors indicate different population sizes; purple: *N* = 50000, red: *N* = 5000, orange: *N* = 500 yellow: *N* = 50. Each set of simulations was obtained from 1000 simulations, of which those resulting in hybrid speciation were retained. We only show the time to hybridization if we observed at least 4 occurrences of hybrid speciation. Standard errors are represented by black bars.
(TIF)

S16 Fig Time to hybrid speciation is relatively unaffected by the dominance of the epistatic interaction, the strength of the epistasis or the linkage architecture (here: Crossed ABBA, Nested ABAB, Nested AABB). Each row of panels represents a different linkage architecture, for which the time to hybrid speciation is shown for codominant (left), recessive (middle), and recessive lethal DMIs (right). We show the average time to hybrid speciation, i.e. to fixation of one of the two hybrid haplotypes, scaled by the size of the population as a function of the recombination rate. Colors indicate different population sizes; purple: *N* = 50000, red: *N* = 5000, orange: *N* = 500 yellow: *N* = 50. Each set of simulations was obtained from 1000 simulations, of which those resulting in hybrid speciation were retained. We only show the time to hybridization if we observed at least 4 occurrences of hybrid speciation. Standard errors are represented by black bars.
(TIF)

S17 Fig The time to hybrid speciation is similar between codominant and recessive DMIs despite a much faster resolution of the two DMIs in the codominant case. We show the time to resolution of the two DMIs (blue and cyan) and of loss of all polymorphism (i.e., a haplotype has fixed; black and gray). We compare the time of fixation of a hybrid haplotype (black) to the average fixation time of a haplotype (gray). In addition, we compare the average time to resolution of both DMIs for all evolutionary outcomes (cyan) and conditioned on the occurrence of hybrid speciation (blue). The linkage architecture used here is “Adjacent ABAB”. Each parameter set was obtained from 1000 simulations. We only display the time to hybrid speciation if we observed at least 4 occurrences of hybrid speciation for the respective parameter combination.
(TIF)

S1 Table Comparison of the parametrization of genetic incompatibilities between the model of [24] (top) and our model (bottom). The derived alleles follow the same nomenclature. However, the ancestral alleles, which we called “a” and “b” are represented by “x” in the Schumer model.
(PDF)

S1 File Supplementary information. (PDF)
