# Supplementary figures and images for: In search of the Goldilocks zone for hybrid speciation
Source: PLoS Genet. 2018 Sep 7;14(9):e1007613. doi: 10.1371/journal.pgen.1007613 (PMC6145587; doi:10.1371/journal.pgen.1007613)

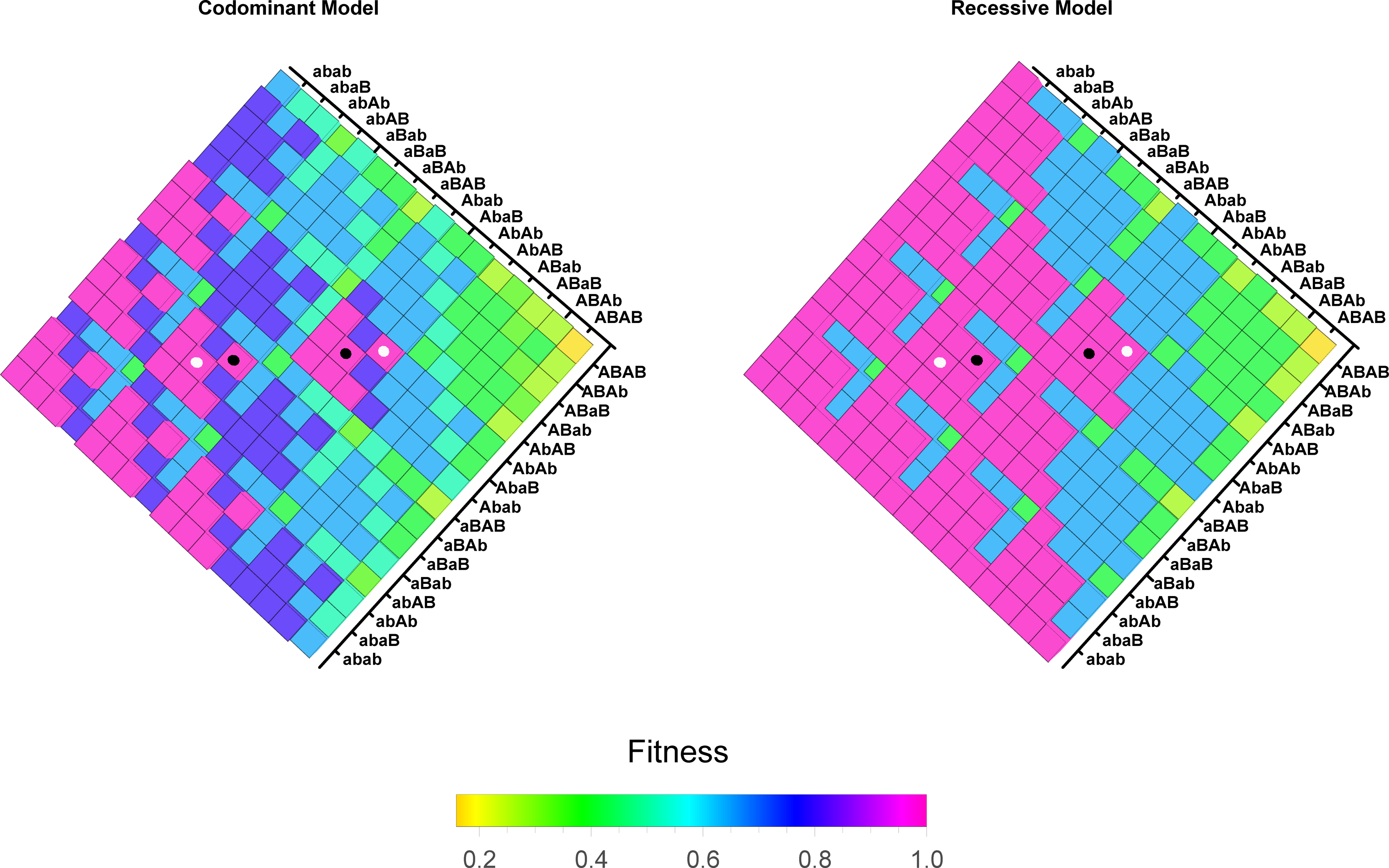

Supplement: S1 Fig — Here, we assume the “Adjacent ABAB” architecture and drop the indices to improve readability of the figure. The white dots indicate the position of the parental genotypes and the black dots the position of the hybrid speciation haplotypes. We use the “default” set of parameters: αi = βi = 0.001, ϵ = −0.2. The fitness advantage of the parental genotypes as compared to the ancestral genotype, chosen here as 0.0014 ≈ 0.004, is too small to be visible in the illustration. The arrangement of the haplotypes is arbitrary. (TIF) [file pgen.1007613.s001.tif]

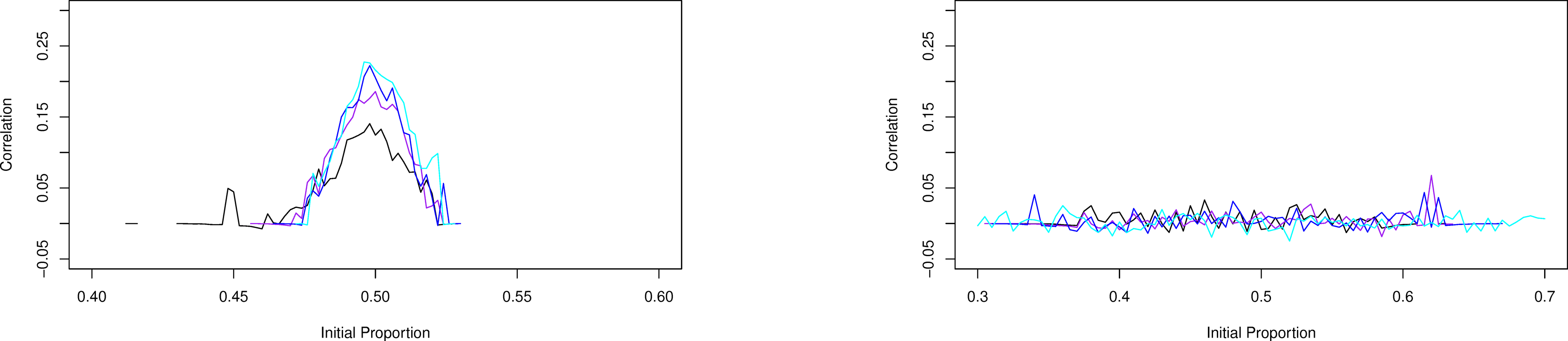

Supplement: S2 Fig — Each incompatibility pair is located on a different chromosome. Colors indicate recombination rates between the Ak and Bk loci involved in the DMIs: r = 0.5 in black, r = 0.1 in purple, r = 0.05 in blue and r = 0.005 in cyan. Other parameters used are: αi = βj = 0.001, ϵ = −0.2, N = 5000. (TIF) [file pgen.1007613.s002.tif]

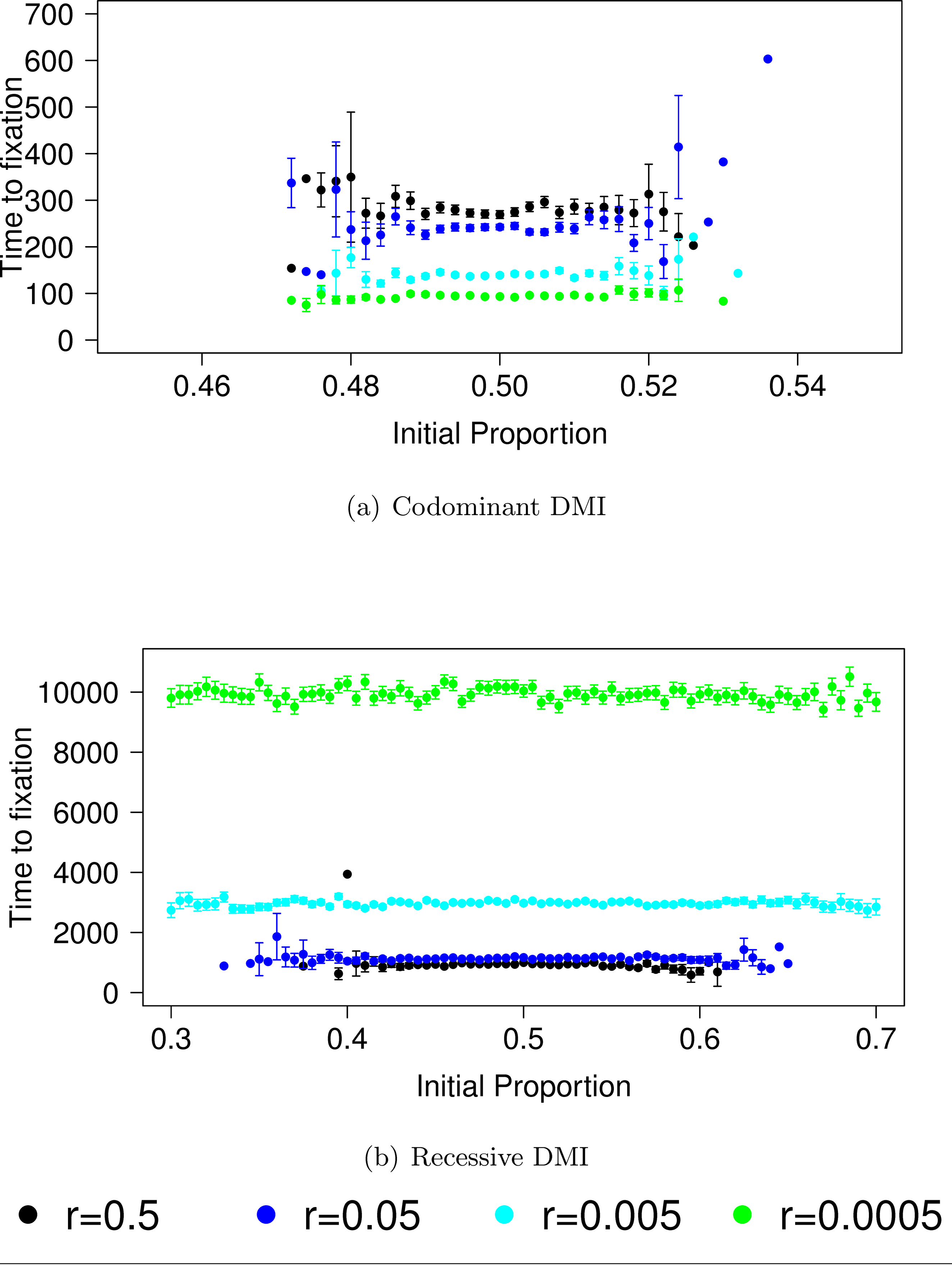

Supplement: S3 Fig — Parameters used here are: αi = βj = 0.001, ϵ = −0.2, N = 5000. For any initial frequency, we performed 1000 simulations and then extracted those simulations that resulted in hybrid speciation. (TIF) [file pgen.1007613.s003.tif]

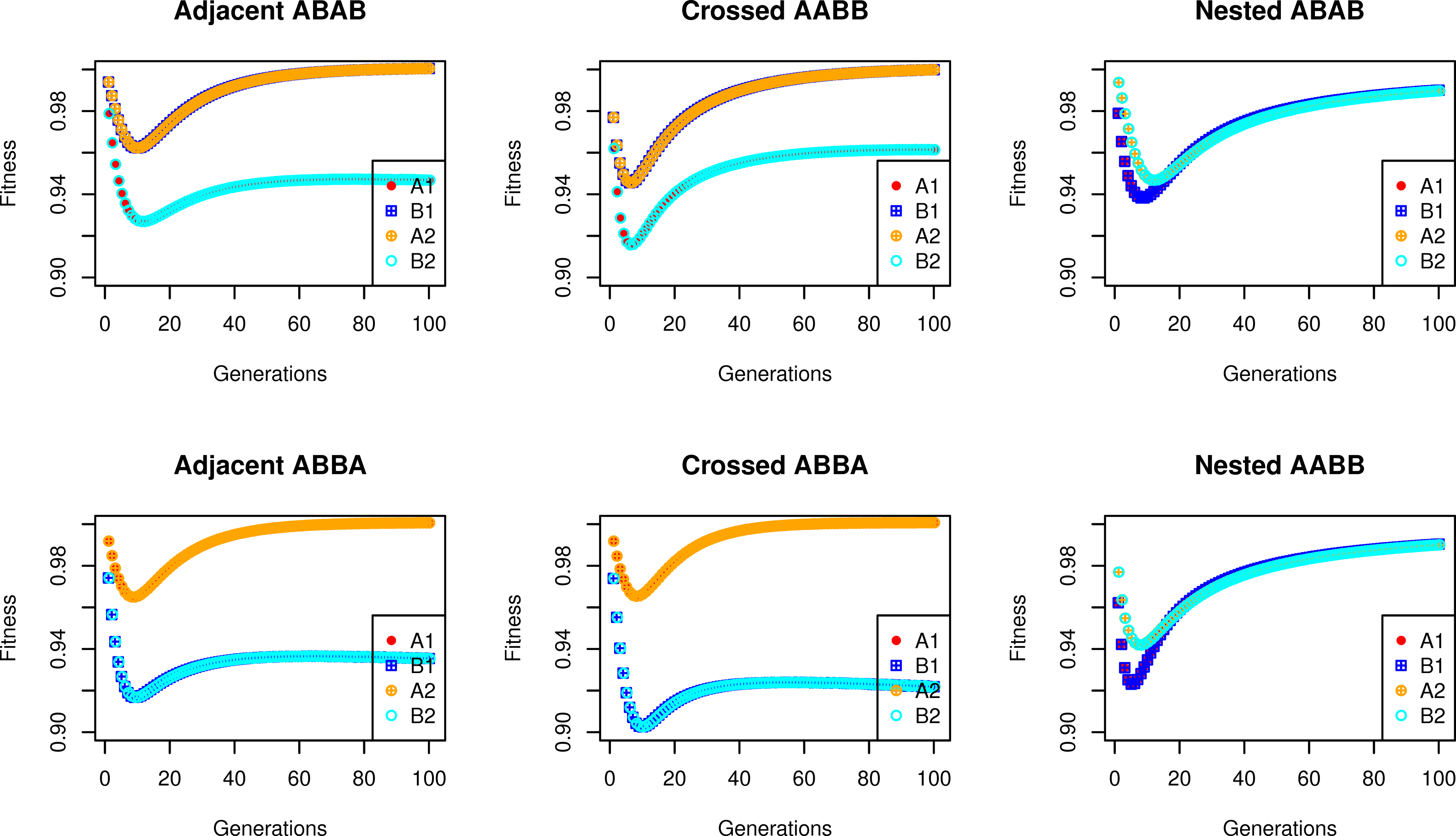

Supplement: S4 Fig — For the “Adjacent ABAB” and “Crossed AABB” architectures, alleles A2 and B1 have a marginal fitness advantage over A1 and B2. For the “Adjacent ABBA” and “Crossed ABBA” architectures, alleles A1 and A2 have a marginal fitness advantage over B1 and B2. Lastly, for the “Nested ABAB” and “Nested AABB” architectures, alleles A2 and B2 have a marginal fitness advantage over A1 and B1. All DMIs represented here are codominant. Both parental populations contributed equally to the hybrid population, ip = 0.5. All loci are equidistant with a recombination rate between adjacent loci of r = 0.2. Others parameters used are: αi = βj = 0.001, ϵ = −0.2. (TIF) [file pgen.1007613.s004.tif]

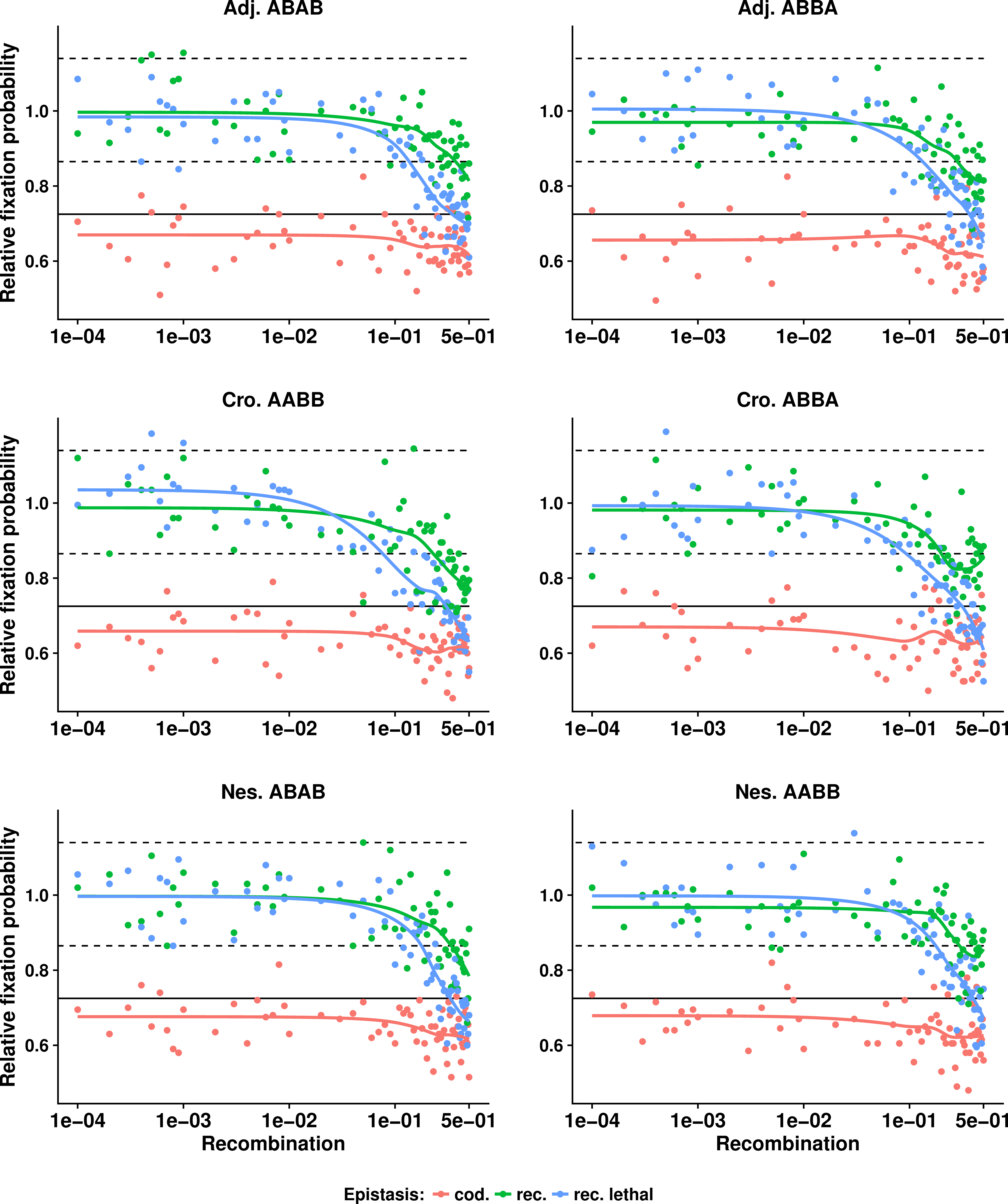

Supplement: S5 Fig — This probability is displayed relative to the fixation probability of a similar neutral marker that appears in a hybrid individual, pn = 1/5000. The x-axis corresponds to the recombination rate between the different incompatible loci; the neutral marker is always located on a different chromosome. We estimated the fixation probabilities over 106 replicates. Black lines indicate when the fixation probability is significantly distinct from pn = 1/5000: any data point below the solid lines is significantly different under a Bonferroni correction (i.e. ppn<0.725 or ppn>1.305). Dashed lines correspond to 95% confidence intervals without Bonferroni correction and provide a visual guide. (TIF) [file pgen.1007613.s005.tif]

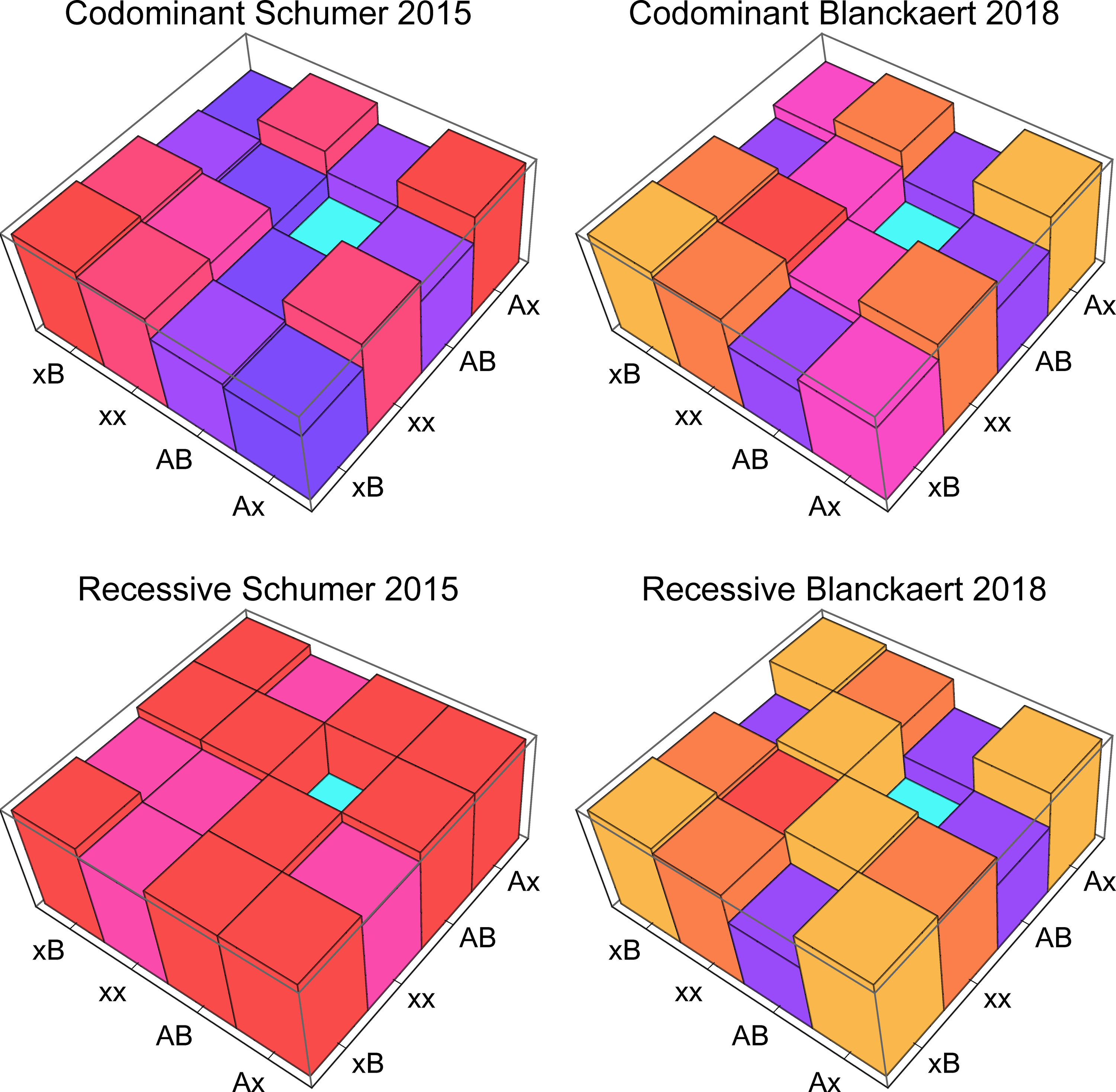

Supplement: S6 Fig — To facilitate the comparison, we use the notation of [24], as presented in Table S1 Table. Parametrization was chosen such that the fitness differences between parental and ancestral genotypes and the strength of the incompatibility for the homozygote incompatible ABAB are the same. (TIF) [file pgen.1007613.s006.tif]

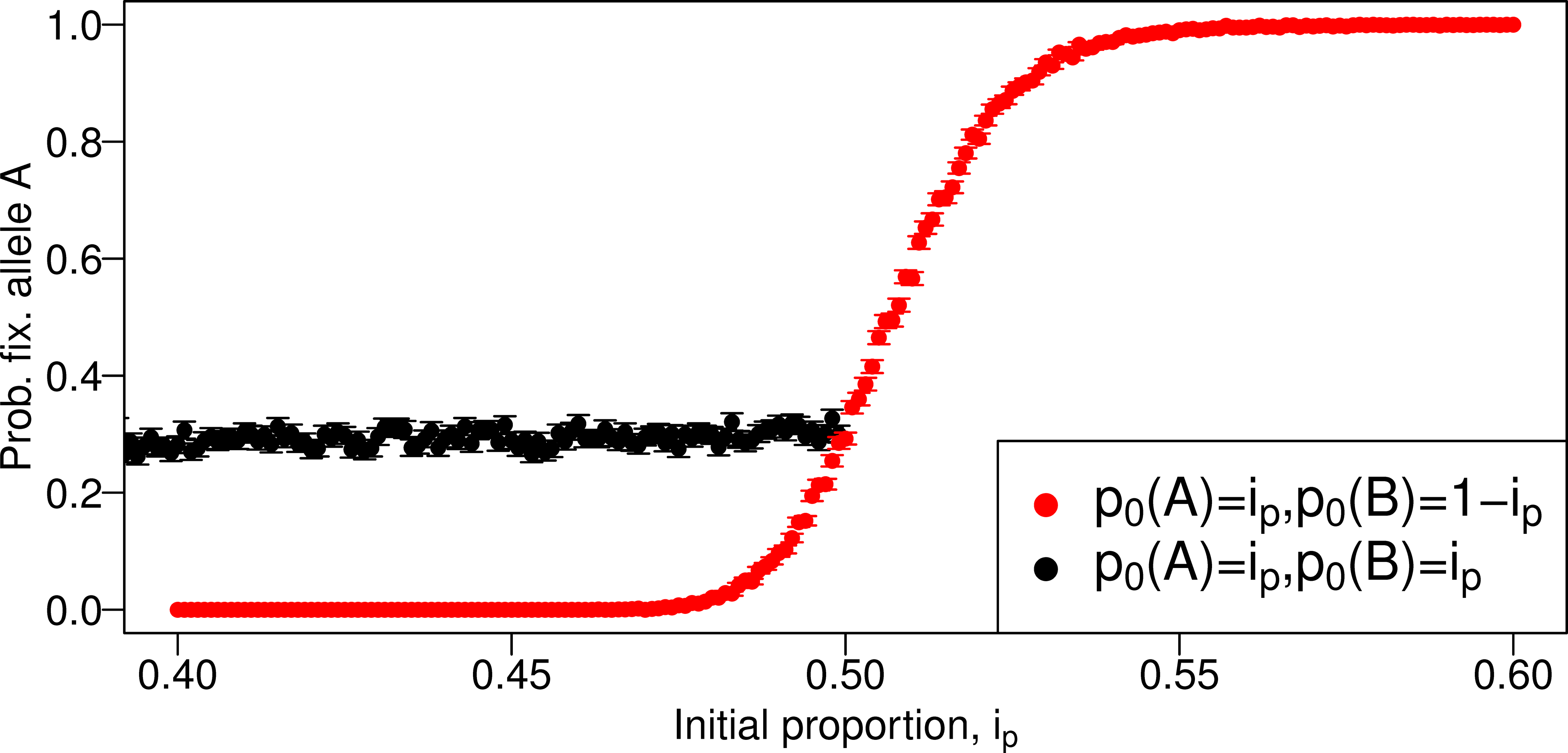

Supplement: S7 Fig — In one case (red dots), haplotype Ab is introduced at proportion ip and aB at proportion 1 − ip. To demonstrate that the frequency-dependent effect is dependent on the relative proportion of the A and B allele, in the other case (black dots), both haplotypes Ab and aB are introduced at proportion iq and the ancestral haplotype ab is introduced at proportion 1 − 2iq. (TIF) [file pgen.1007613.s007.tif]

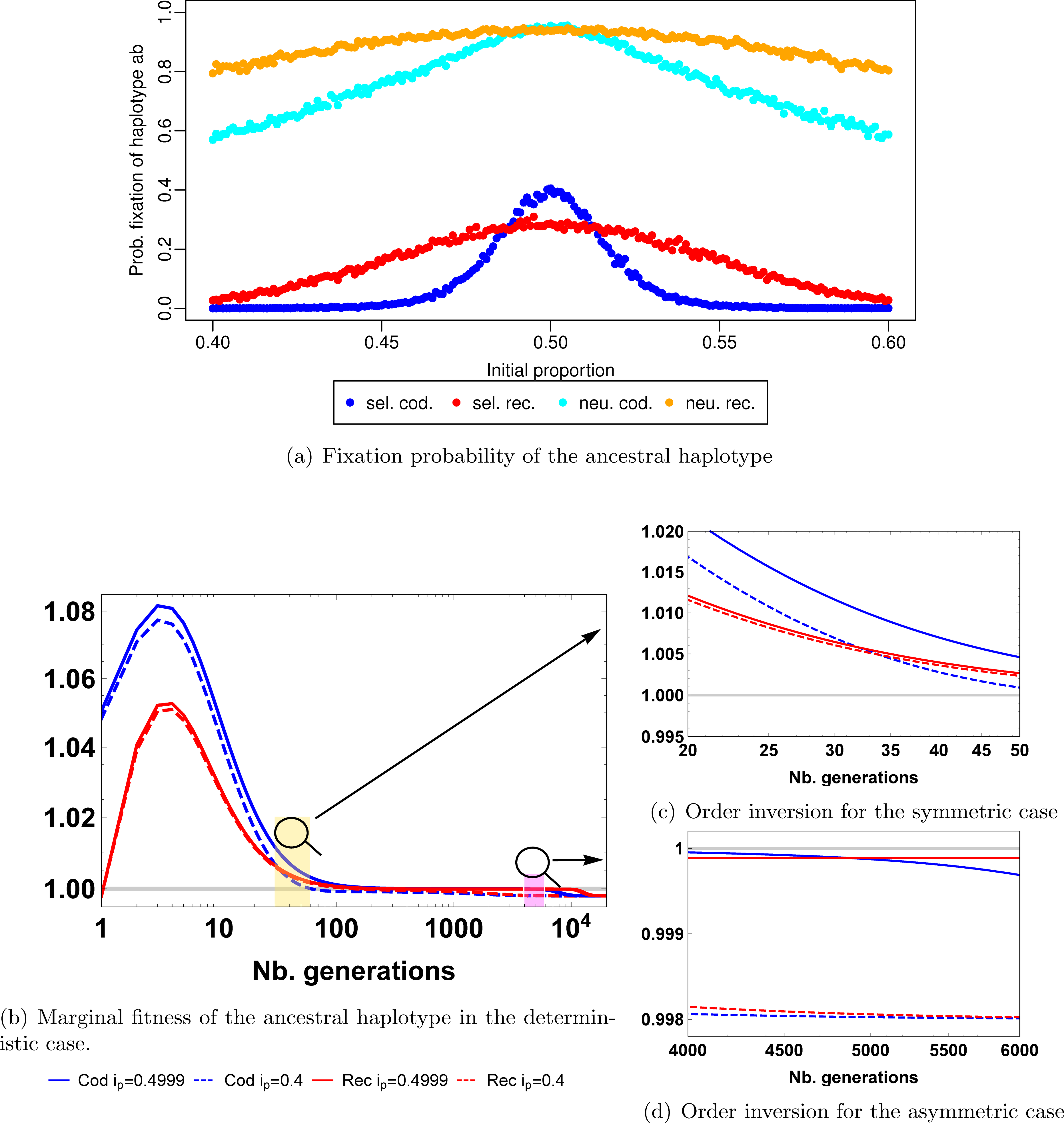

Supplement: S8 Fig — Panel (a) shows the probability of recovering the ancestral haplotype ab in an isolated hybrid population for the two dominance schemes both for neutral (α = β = 0) and slightly advantageous mutations (α = β = 0.001). Panel (b) tracks the marginal fitness of the ab haplotype in the deterministic model, for an (almost) symmetric contact (solid lines) and an asymmetric proportion of the parental genomes (dashed lines). Blue and red dots correspond to the data from panel (a). Due to the masking effect of recessivity, the marginal fitness of ab is always lower in the recessive case than in the codominant case. Panels (c) and (d) correspond to subsets of panel (b) and illustrate at which point the order of probabilities is reversed for symmetric (panel (d)) and asymmetric contact (panel (c)). Order inversion means that from this time point onwards, masking no longer provides an advantage to the derived alleles. The longer the derived alleles are masked by recessivity, the more likely the ancestral haplotype will fix while both derived alleles are present at an equally low frequency, and therefore susceptible to be lost through drift. Other parameters used are: N = 5000, ϵ = −0.2 and α = β = 0.001 for panels (b-d). In panel (a), each data point is obtained from 2000 simulations. (TIF) [file pgen.1007613.s008.tif]

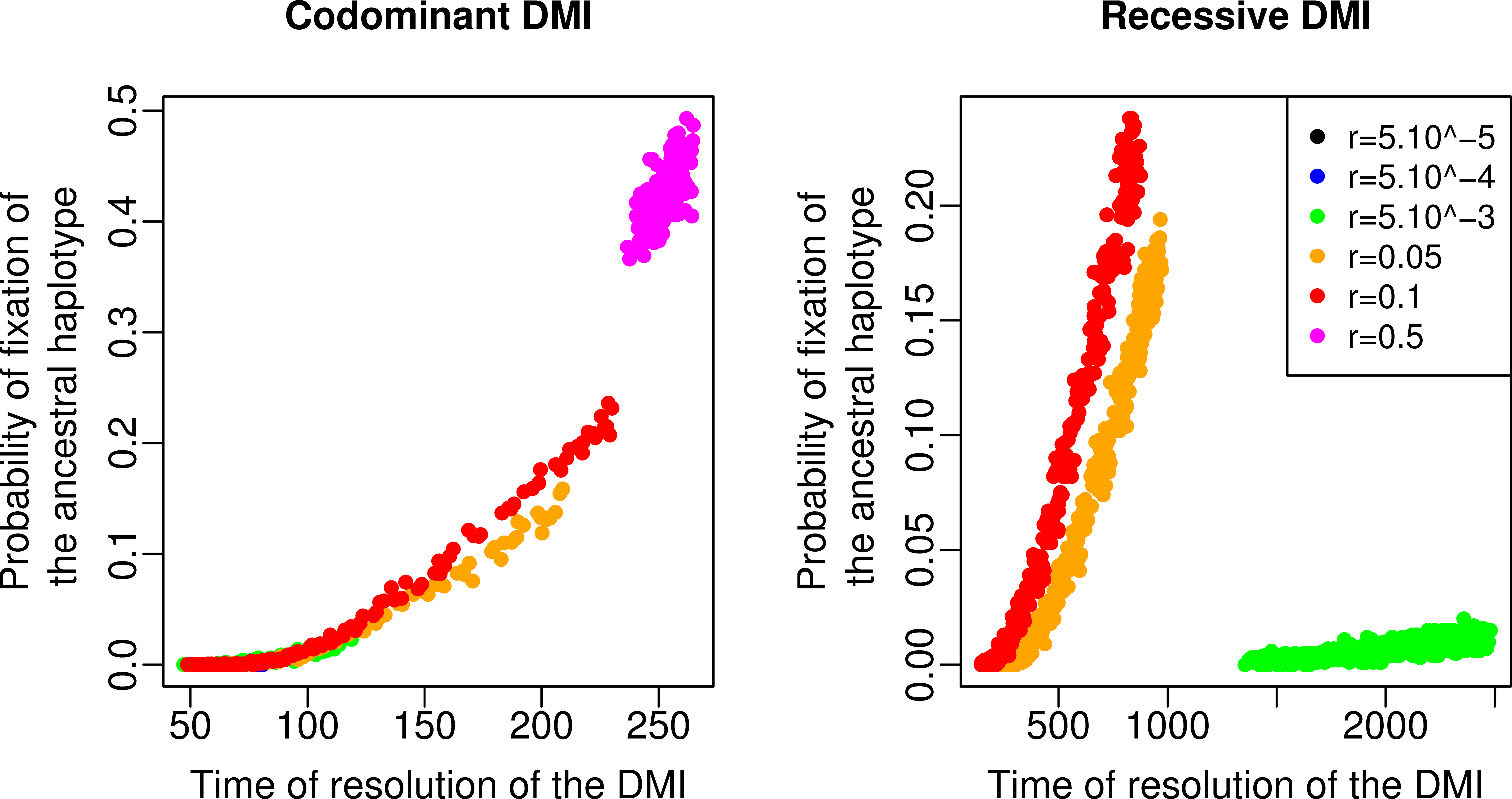

Supplement: S9 Fig — Colors represent different recombination rates between the A and B loci. Each dot is the result of 1000 simulations. Parameters used are: α = β = 0.001 and N = 5000. (TIF) [file pgen.1007613.s009.tif]

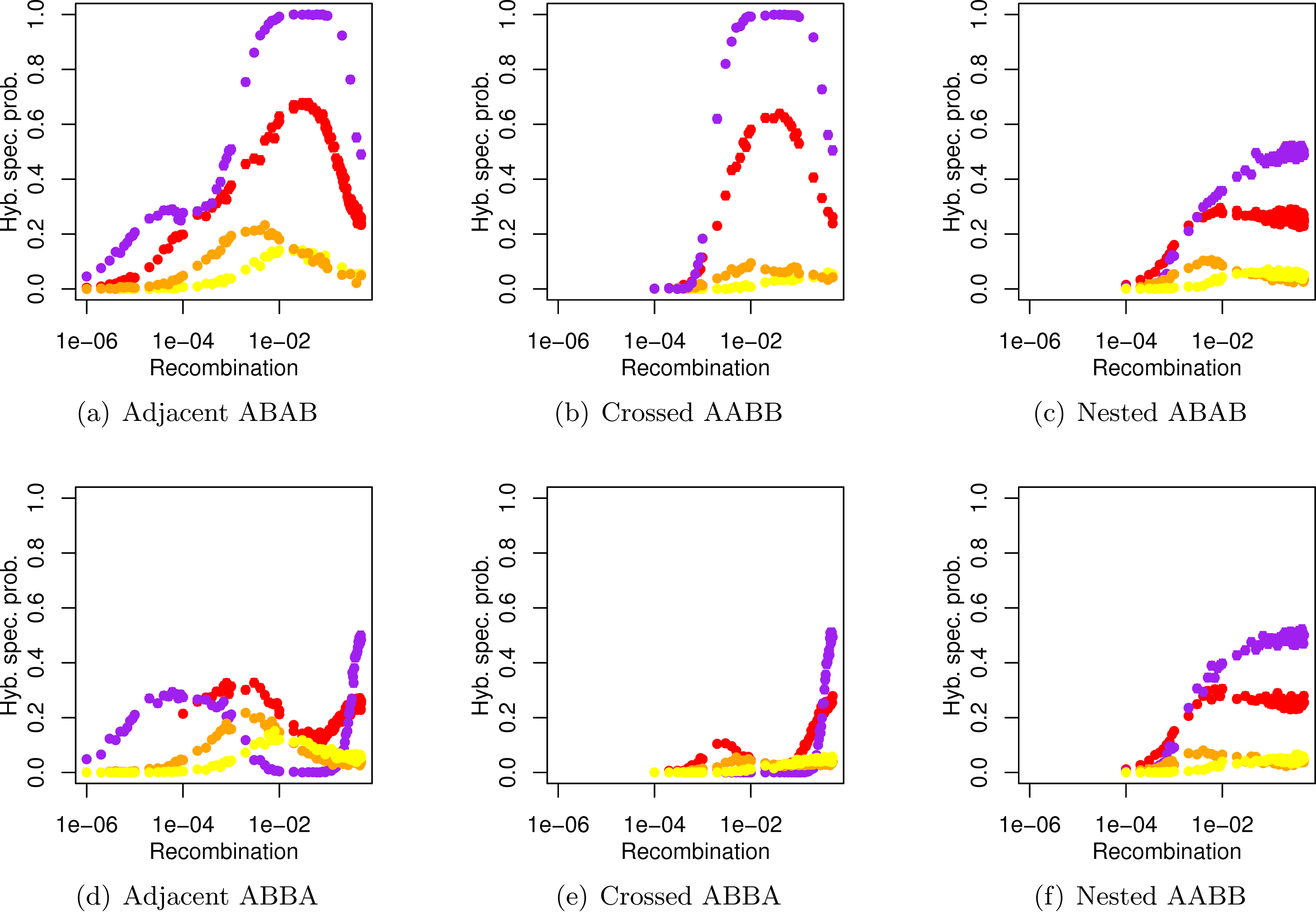

Supplement: S10 Fig — We represent this probability for four different population sizes: N = 50 in yellow, N = 500 in orange, N = 5000 in red and N = 50000 in purple. Other parameters are αk = βj = 0.001, ϵ = −0.2 and ip = 0.5 (i.e. the contribution of both parental populations is symmetric). This figure corresponds to Fig 5 for codominant DMIs. (TIF) [file pgen.1007613.s010.tif]

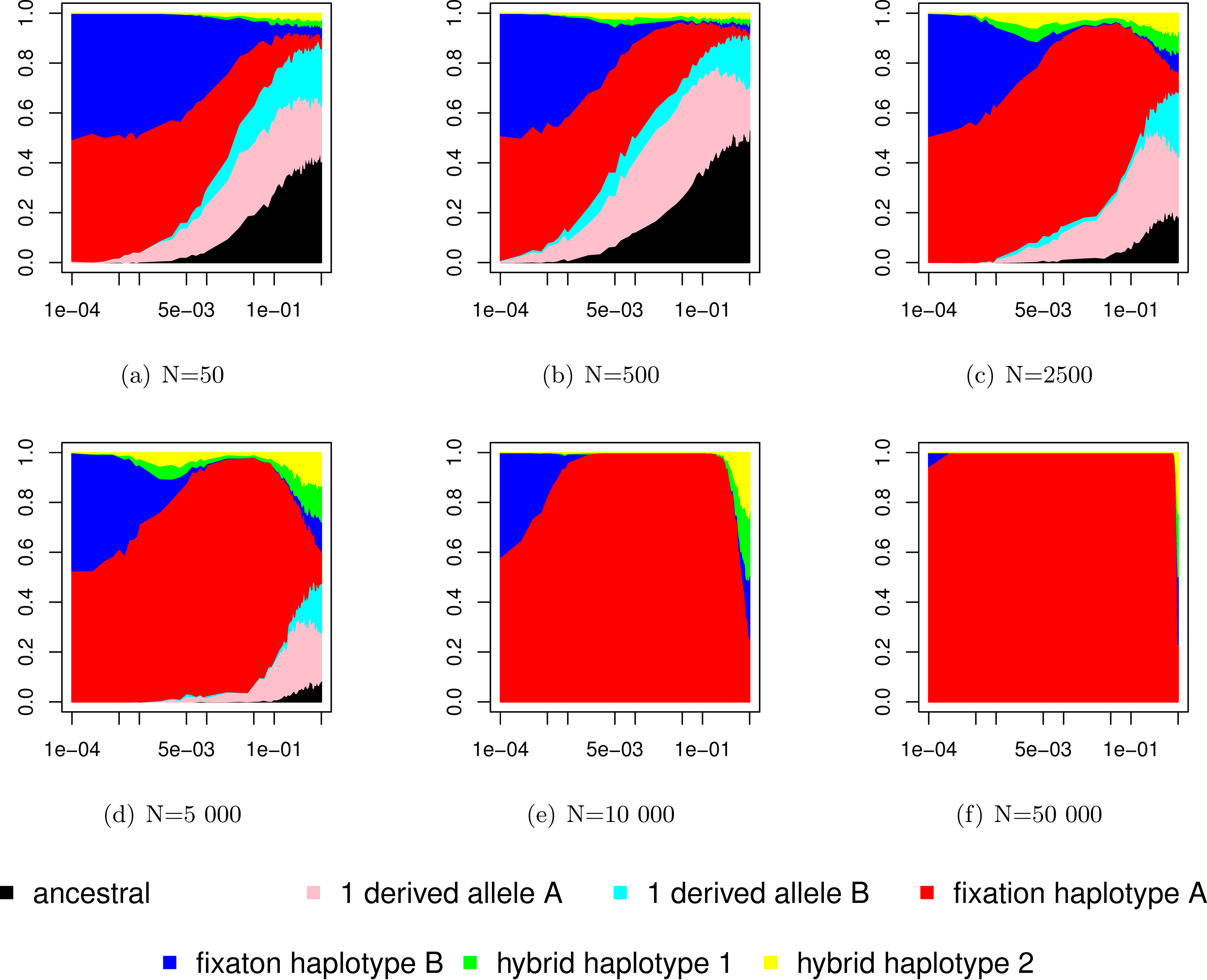

Supplement: S11 Fig — The x-axis corresponds to the recombination rate and each panel shows a different population size. To better illustrate the underlying mechanisms, we represent both hybrid haplotypes separately with A1B2b1a2 in green and a1b2B1A2 in yellow. (TIF) [file pgen.1007613.s011.tif]

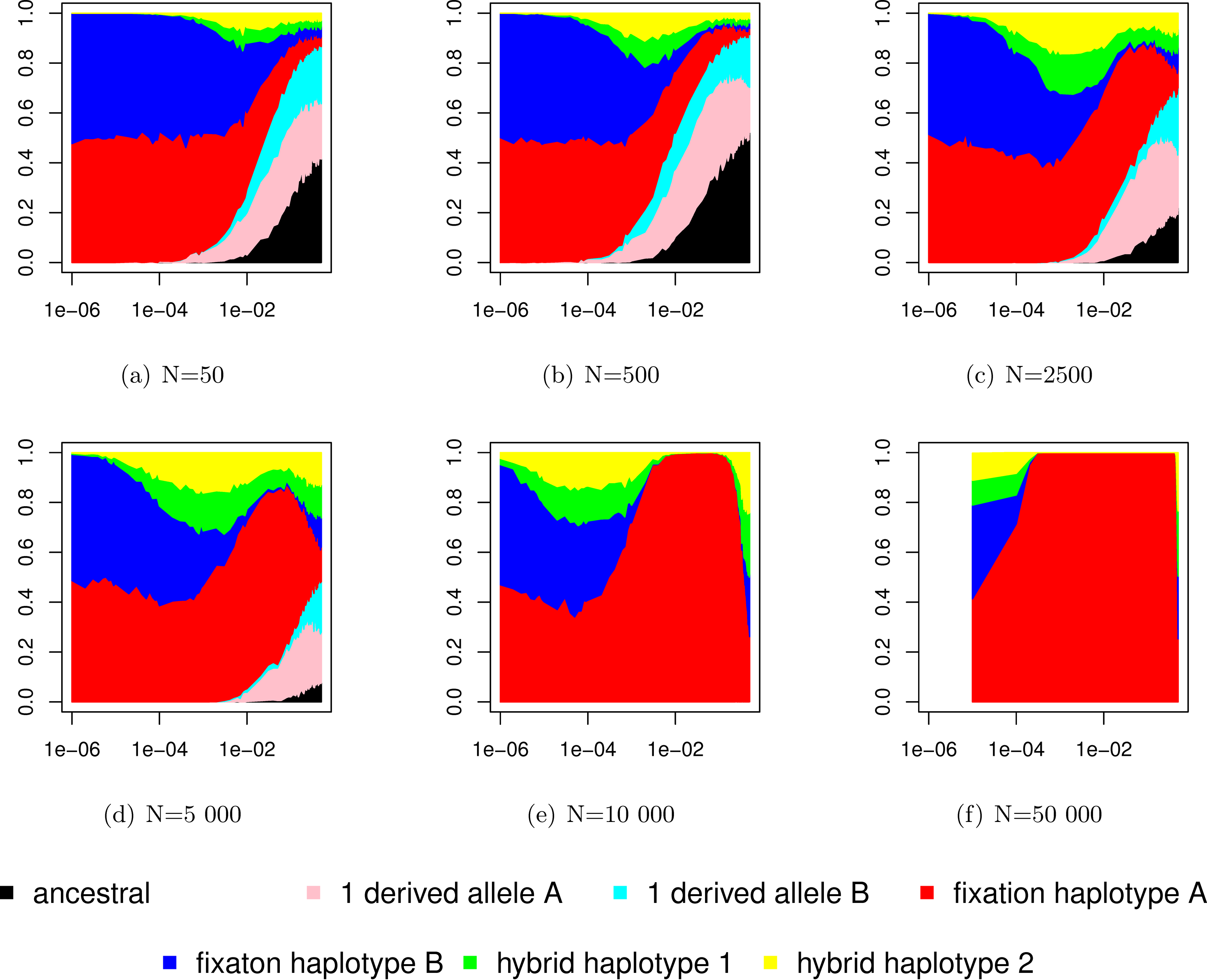

Supplement: S12 Fig — The x-axis corresponds to the recombination rate and each panel shows a different population size. To better illustrate the underlying mechanisms, we represent both hybrid haplotypes separately with A1B2b1a2 in green and a1b2B1A2 in yellow. (TIF) [file pgen.1007613.s012.tif]

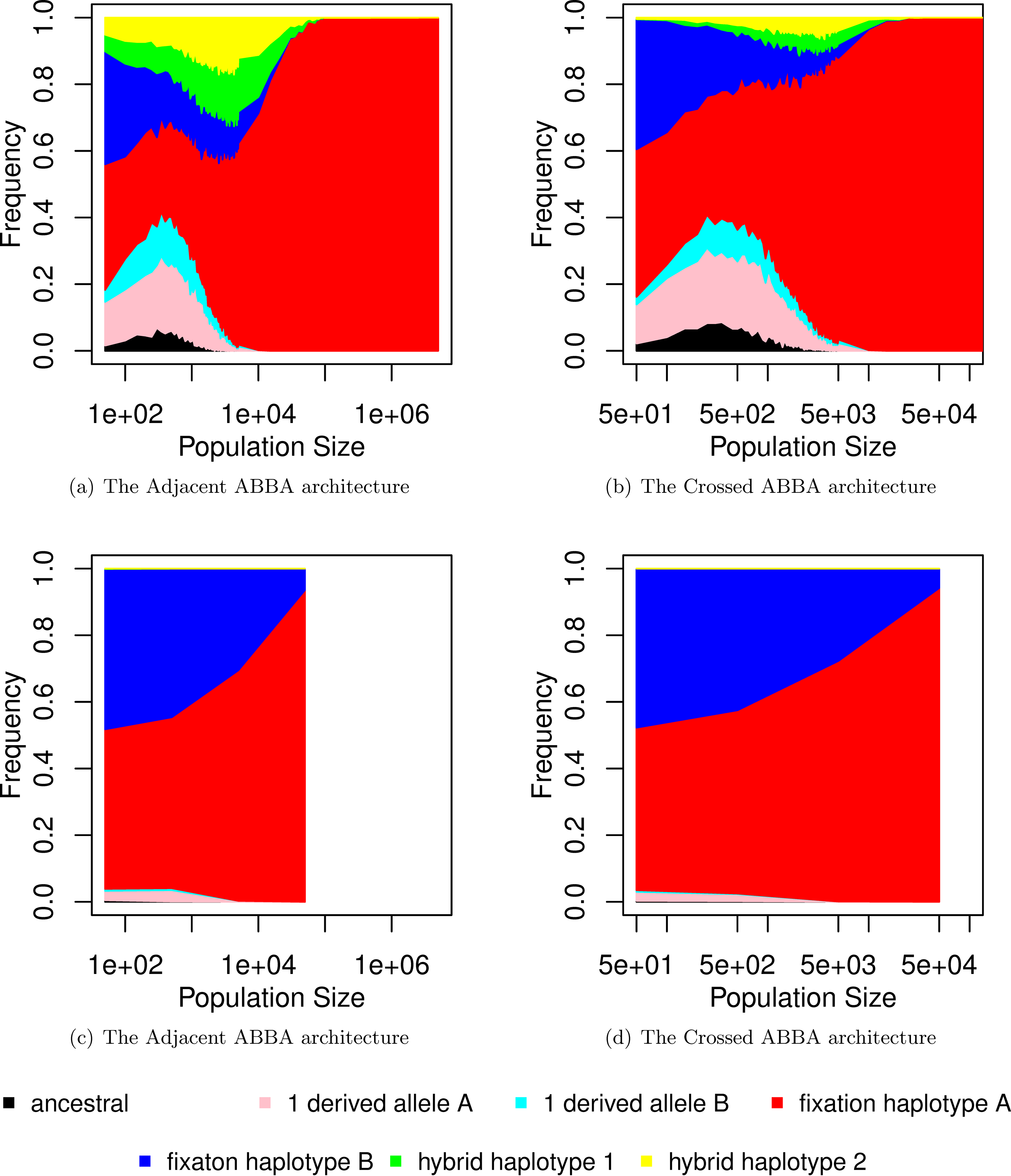

Supplement: S13 Fig — We focus on the two architectures that displayed a second local maximum of hybrid speciation probability for low recombination rate, here r = 0.005. Each color corresponds to a different evolutionary outcome. (TIF) [file pgen.1007613.s013.tif]

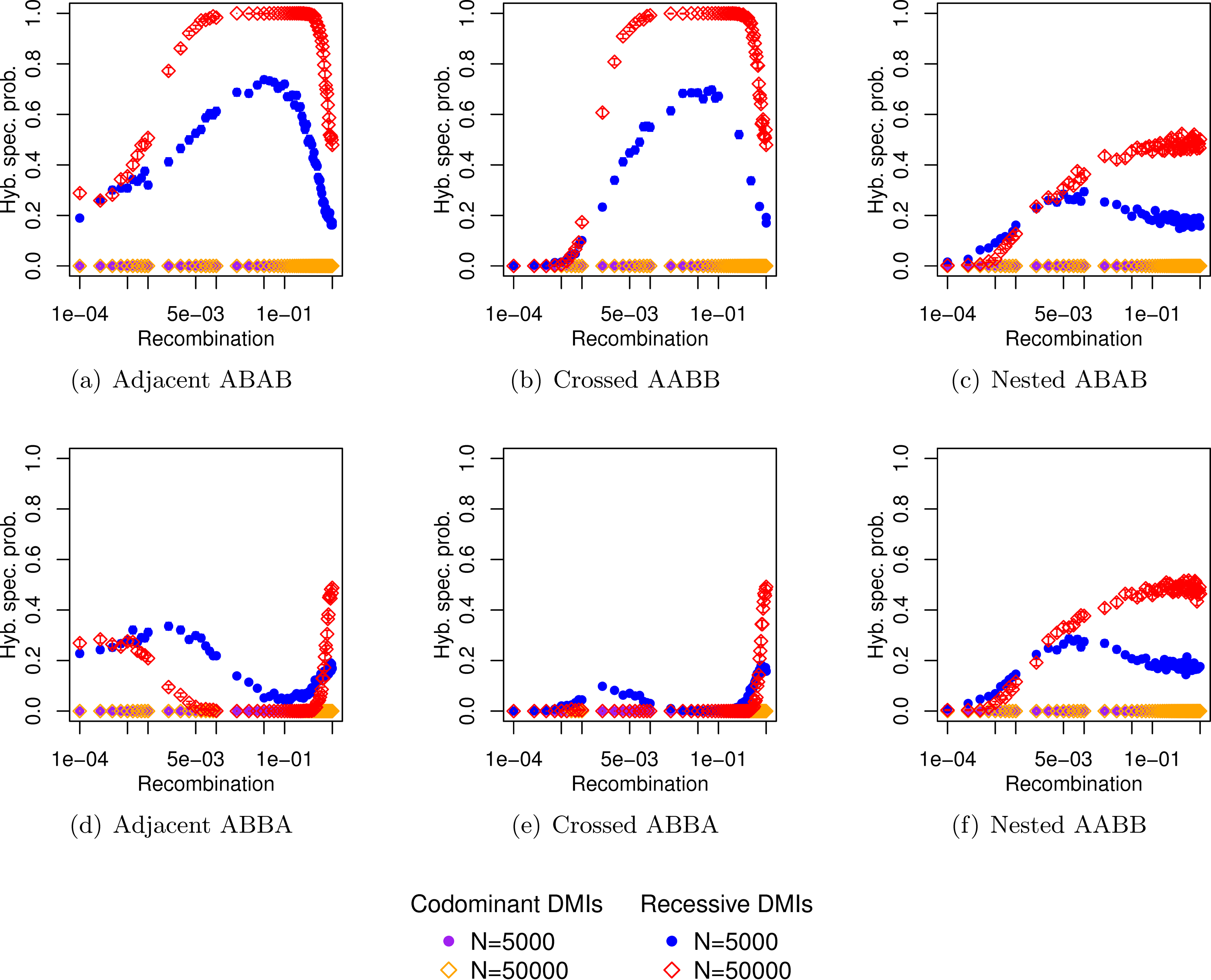

Supplement: S14 Fig — We consider both codominant and recessive DMIs and two population sizes. Hybrid speciation does not occur for codominant DMIs regardless of population size. For recessive DMIs, the hybrid speciation probability is qualitatively identical to the less deleterious case, see S10 Fig with the blue dots here matching the red dots in S10 Fig and the red diamonds the purple dots in S10 Fig. Parameters used are: α = β = 0.001, ϵ = −0.99. (TIF) [file pgen.1007613.s014.tif]

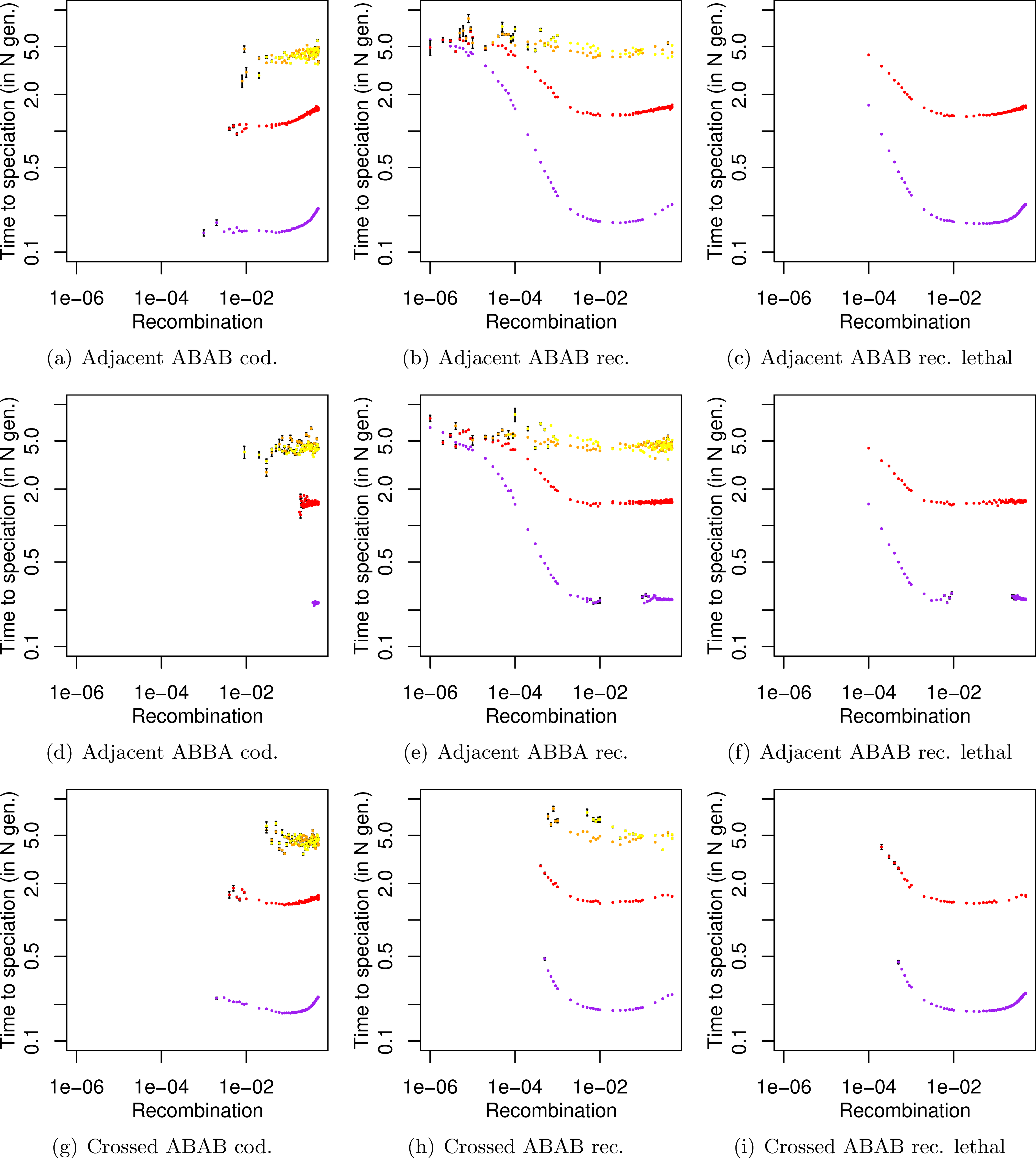

Supplement: S15 Fig — Each row of panels represents a different linkage architecture, for which the time to hybrid speciation is shown for codominant (left), recessive (middle), and recessive lethal DMIs (right). We show the average time to hybrid speciation, i.e. to fixation of one of the two hybrid haplotypes, scaled by the size of the population as a function of the recombination rate. Colors indicate different population sizes; purple: N = 50000, red: N = 5000, orange: N = 500 yellow: N = 50. Each set of simulations was obtained from 1000 simulations, of which those resulting in hybrid speciation were retained. We only show the time to hybridization if we observed at least 4 occurrences of hybrid speciation. Standard errors are represented by black bars. (TIF) [file pgen.1007613.s015.tif]

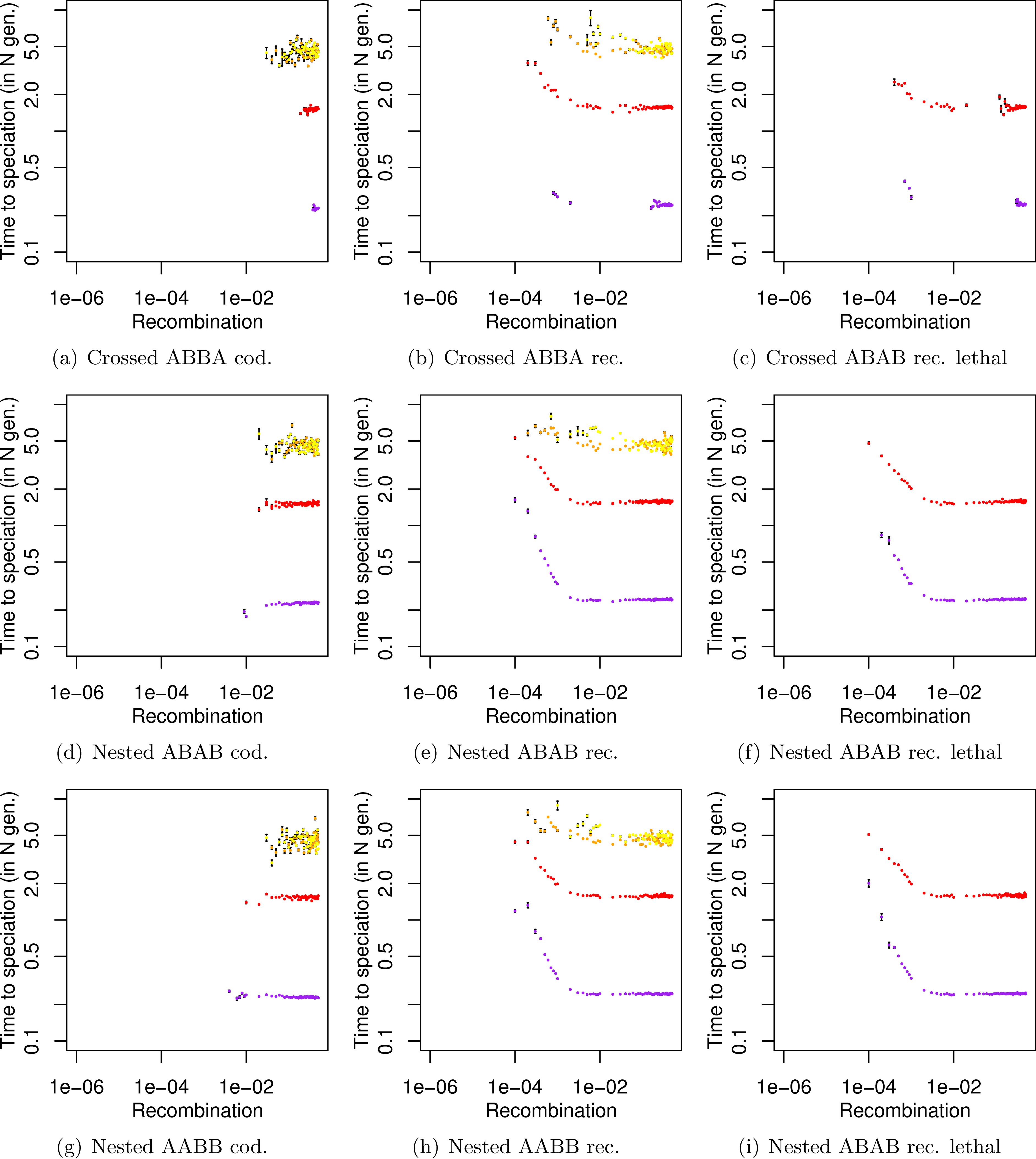

Supplement: S16 Fig — Each row of panels represents a different linkage architecture, for which the time to hybrid speciation is shown for codominant (left), recessive (middle), and recessive lethal DMIs (right). We show the average time to hybrid speciation, i.e. to fixation of one of the two hybrid haplotypes, scaled by the size of the population as a function of the recombination rate. Colors indicate different population sizes; purple: N = 50000, red: N = 5000, orange: N = 500 yellow: N = 50. Each set of simulations was obtained from 1000 simulations, of which those resulting in hybrid speciation were retained. We only show the time to hybridization if we observed at least 4 occurrences of hybrid speciation. Standard errors are represented by black bars. (TIF) [file pgen.1007613.s016.tif]

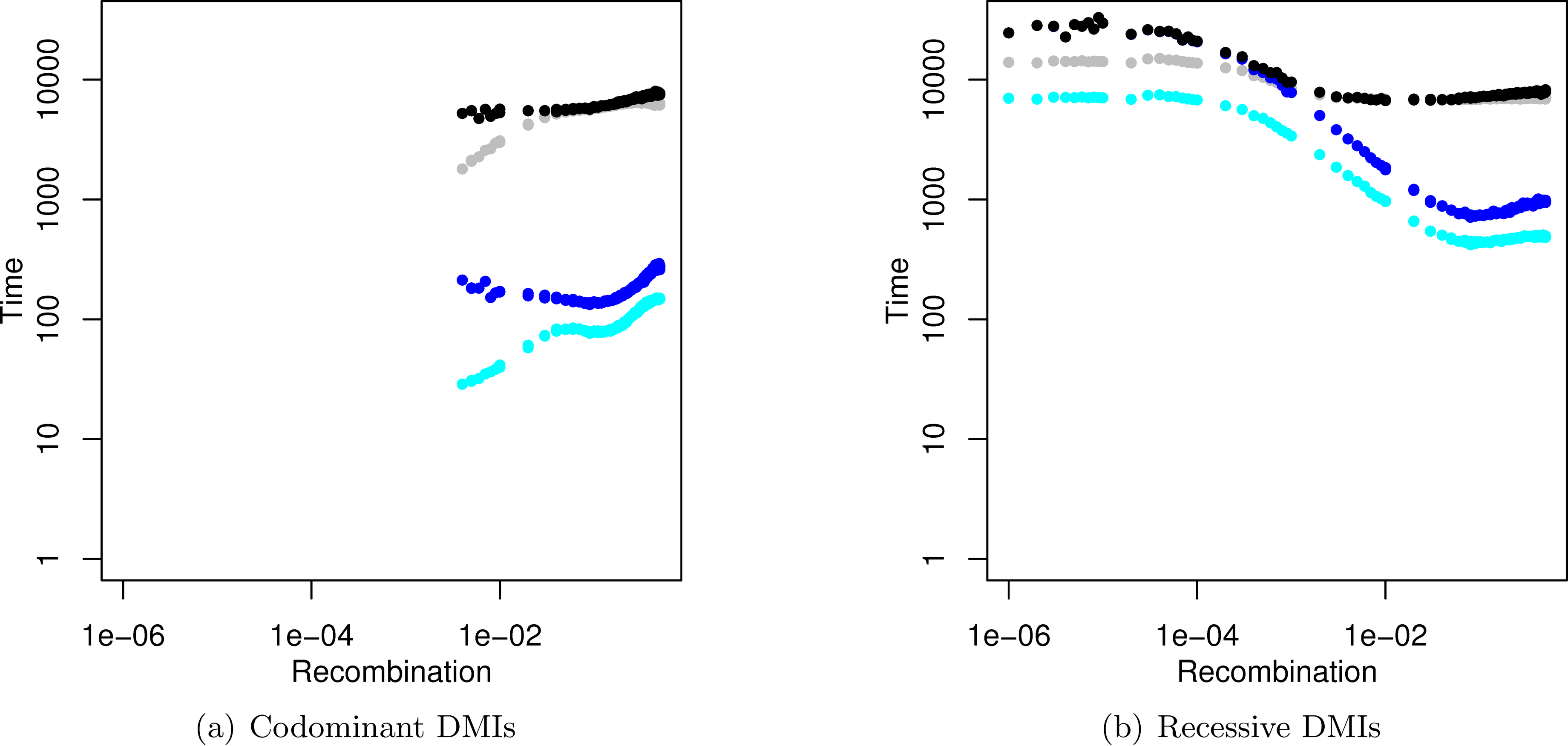

Supplement: S17 Fig — We show the time to resolution of the two DMIs (blue and cyan) and of loss of all polymorphism (i.e., a haplotype has fixed; black and gray). We compare the time of fixation of a hybrid haplotype (black) to the average fixation time of a haplotype (gray). In addition, we compare the average time to resolution of both DMIs for all evolutionary outcomes (cyan) and conditioned on the occurrence of hybrid speciation (blue). The linkage architecture used here is “Adjacent ABAB”. Each parameter set was obtained from 1000 simulations. We only display the time to hybrid speciation if we observed at least 4 occurrences of hybrid speciation for the respective parameter combination. (TIF) [file pgen.1007613.s017.tif]
