# Supplementary material for: In search of the Goldilocks zone for hybrid speciation
Source: PLoS Genet. 2018 Sep 7;14(9):e1007613. doi: 10.1371/journal.pgen.1007613 (PMC6145587; doi:10.1371/journal.pgen.1007613)
Supplement: S1 Table — The derived alleles follow the same nomenclature. However, the ancestral alleles, which we called “a” and “b” are represented by “x” in the Schumer model. (PDF) [file pgen.1007613.s018.pdf]

	xB	xx	AB	Ax
xB	1			
xx	1-(1-h_B) s_1	1-s_1		
AB	1-$h_A s_2$	1-$f(h_B s_1, h_A s_2)$	1-s_2	
Ax	1-$f(h_B s_1, h_A s_2)$	1-(1-h_A) s_1	1-h_B s_2	1

	aB	ab	AB	Ab
aB	$(1+\beta)^2$			
ab	$(1+\beta)$	1		
AB	$(1+\alpha)(1+\beta)^2(1+\epsilon)^2$	$(1+\alpha)(1+\beta)(1+\phi\epsilon)$	$(1+\alpha)^2(1+\beta)^2(1+\epsilon)^4$	
Ab	$(1+\alpha)(1+\beta)(1+\phi\epsilon+1)$	$(1+\alpha)$	$(1+\alpha)^2(1+\beta)(1+\epsilon)^2$	$(1+\alpha)^2$

Table 1: Comparison of the parametrization of genetic incompatibilities between the model of Schumer et al. (2015) (top) and our model (bottom). The derived alleles follow the same nomenclature. However, the ancestral alleles, which we called “a” and “b” are represented by “x” in the Schumer model.
