# Supplementary material for: In search of the Goldilocks zone for hybrid speciation
Source: PLoS Genet. 2018 Sep 7;14(9):e1007613. doi: 10.1371/journal.pgen.1007613 (PMC6145587; doi:10.1371/journal.pgen.1007613)
Supplement: S1 File — (PDF) [file pgen.1007613.s019.pdf]

Supplementary File: In search of the Goldilocks zone for hybrid speciation

Alexandre Blanckaert *[1] and Claudia Bank[1]

[1]Instituto Gulbenkian de Ciencia, 2780-156 Oeiras, Portugal

August 17, 2018

Extra figures

*blanckaert.a@gmail.com

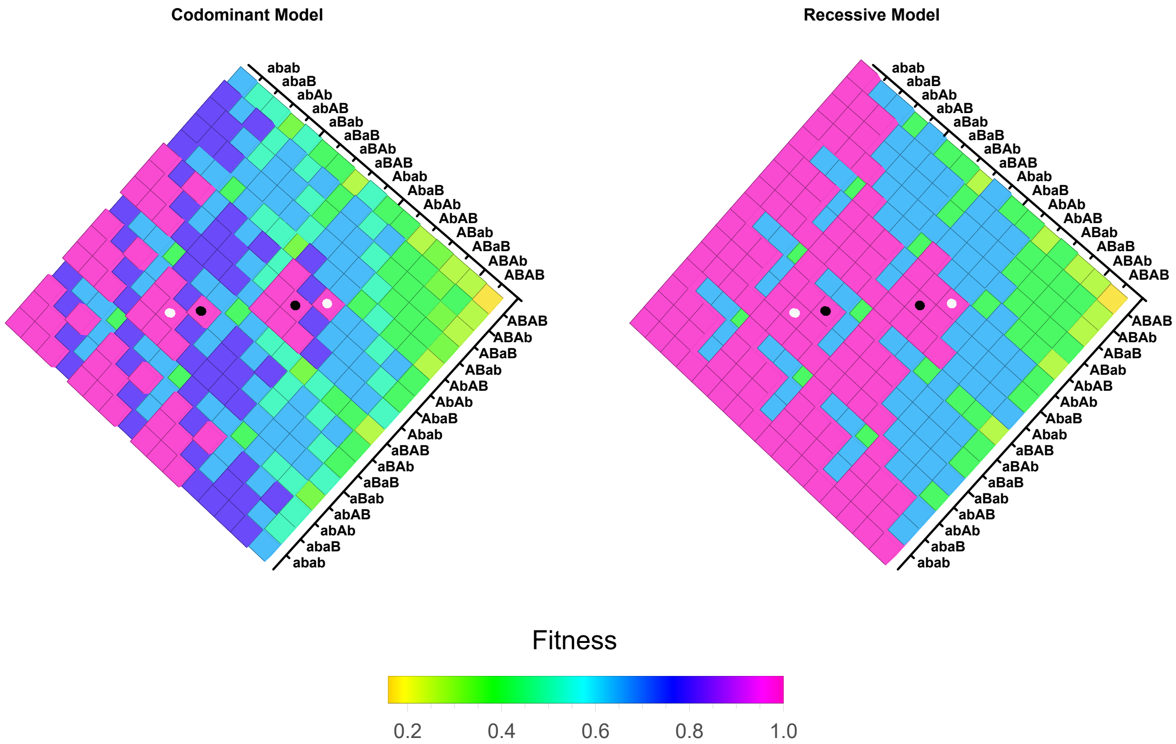

Figure S1: Fitness landscapes for the codominant and recessive models. Here, we assume the "Adjacent ABAB" architecture and drop the indices to improve readability of the figure. The white dots indicate the position of the parental genotypes and the black dots the position of the hybrid speciation haplotypes. We use the "default" set of parameters: $\alpha_i = \beta_i = 0.001, \epsilon = -0.2$. The fitness advantage of the parental genotypes as compared to the ancestral genotype, chosen here as $0.001^4 \approx 0.004$, is too small to be visible in the illustration. The arrangement of the haplotypes is arbitrary.

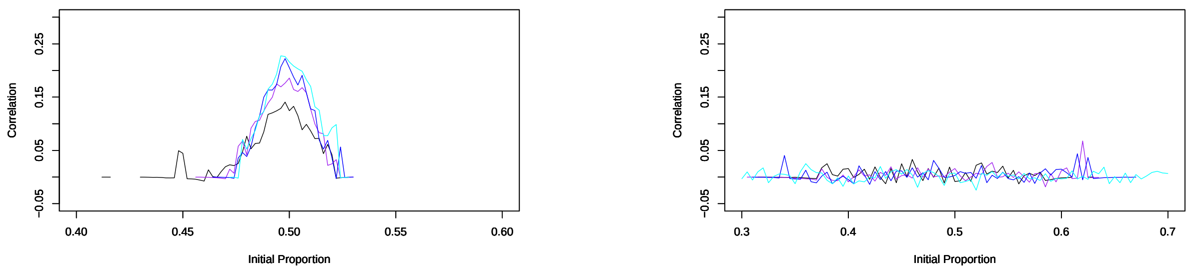

Figure S2: Correlation between fixation of allele A_1 and allele A_2 for codominant (left) and recessive (right) DMIs. Each incompatibility pair is located on a different chromosome. Colors indicate recombination rates between the A_k and B_k loci involved in the DMIs: $r = 0.5$ in black, $r = 0.1$ in purple, $r = 0.05$ in blue and $r = 0.005$ in cyan. Other parameters used are: $\alpha_i = \beta_j = 0.001, \epsilon = -0.2, N = 5000$.

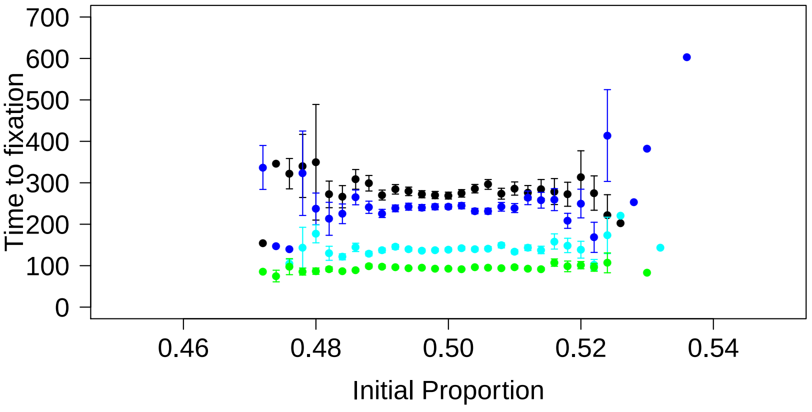

(a) Codominant DMI

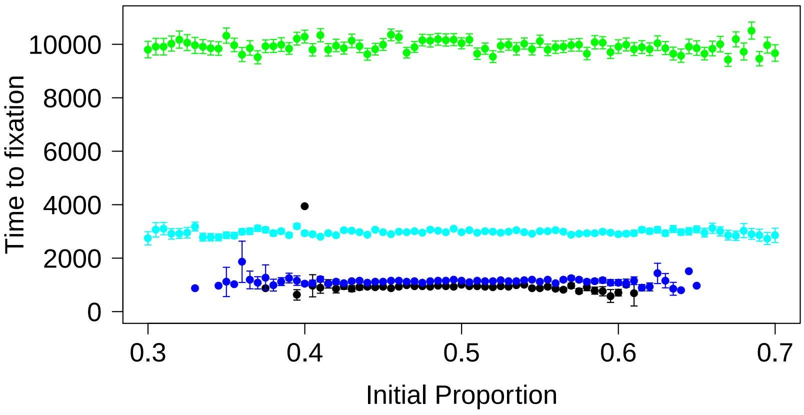

(b) Recessive DMI

● r=0.5 ● r=0.05 ● r=0.005 ● r=0.0005

Figure S3: Time to resolution of both DMIs conditioning on hybrid speciation. Parameters used here are: $\alpha_i = \beta_j = 0.001, \epsilon = -0.2, N = 5000$. For any initial frequency, we performed 1000 simulations and then extracted those simulations that resulted in hybrid speciation.

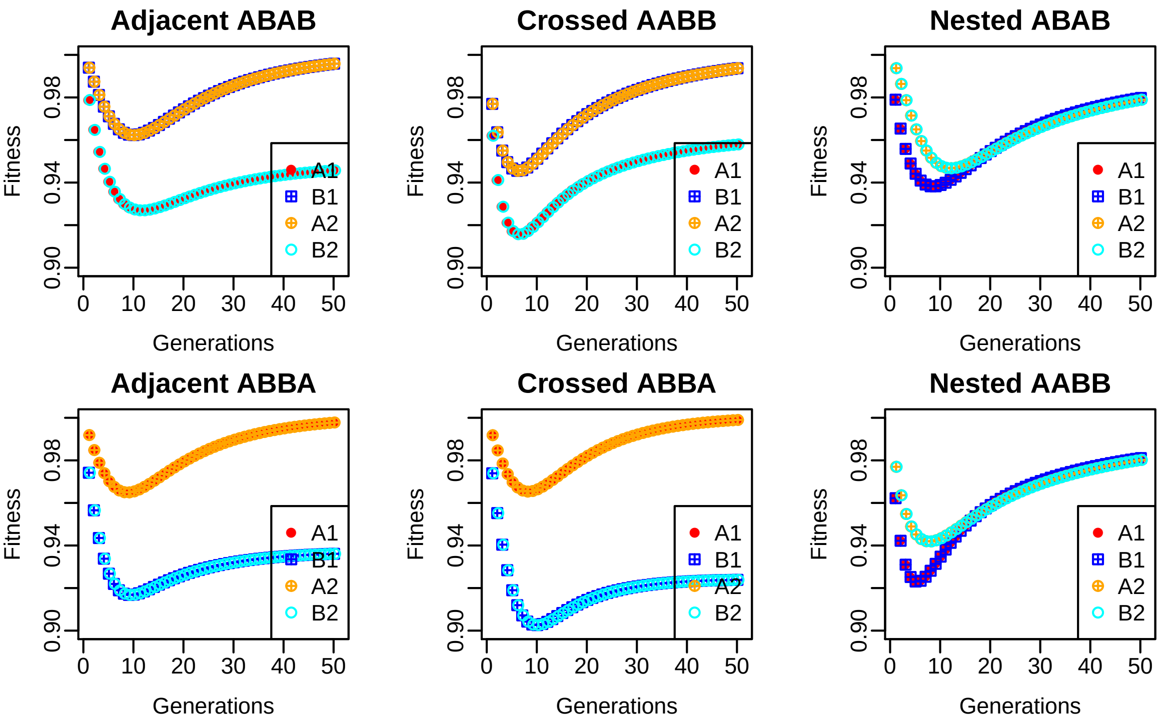

Figure S4: Marginal fitness of the four derived alleles in the deterministic model (no drift) for the first 100 generations. For the "Adjacent ABAB" and "Crossed AABB" architectures, alleles A_2 and B_1 have a marginal fitness advantage over A_1 and B_2. For the "Adjacent ABBA" and "Crossed ABBA" architectures, alleles A_1 and A_2 have a marginal fitness advantage over B_1 and B_2. Lastly, for the "Nested ABAB" and "Nested AABB" architectures, alleles A_2 and B_2 have a marginal fitness advantage over A_1 and B_1. All DMIs represented here are codominant. Both parental populations contributed equally to the hybrid population, $i_p = 0.5$. All loci are equidistant with a recombination rate between adjacent loci of $r = 0.2$. Others parameters used are: $\alpha_i = \beta_j = 0.001, \epsilon = -0.2$.

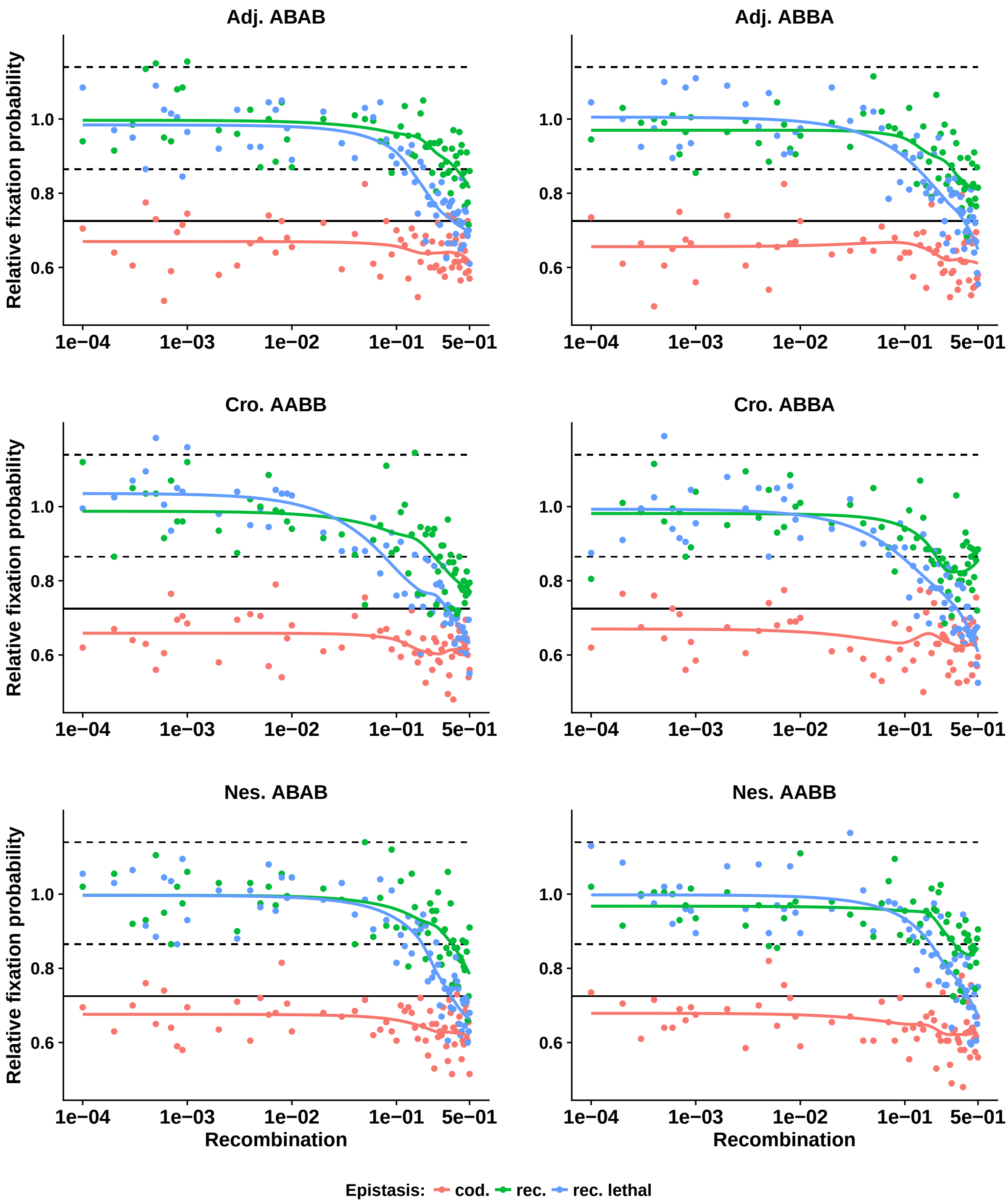

Figure S5: Relative fixation probability of an unlinked neutral marker introduced by a parental individual migrating into the monomorphic hybrid population. This probability is displayed relative to the fixation probability of a similar neutral marker that appears in a hybrid individual, $p_n = 1/5000$. The x-axis corresponds to the recombination rate between the different incompatible loci; the neutral marker is always located on a different chromosome. We estimated the fixation probabilities over 10^6 replicates. Black lines indicate when the fixation probability is significantly distinct from $p_n = 1/5000$: any data point below the solid lines is significantly different under a Bonferroni correction (i.e. $\frac{p}{p_n} < 0.725$ or $\frac{p}{p_n} > 1.305$). Dashed lines correspond to 95% confidence intervals without Bonferroni correction and provide a visual guide.

	xB	xx	AB	Ax
xB	1			
xx	1-(1-h_B) s_1	1-s_1		
AB	1-$h_A s_2$	1-$f(h_B s_1, h_A s_2)$	1-s_2	
Ax	1-$f(h_B s_1, h_A s_2)$	1-(1-h_A) s_1	1-h_B s_2	1

	aB	ab	AB	Ab
aB	$(1+\beta)^2$			
ab	$(1+\beta)$	1		
AB	$(1+\alpha)(1+\beta)^2(1+\epsilon)^2$	$(1+\alpha)(1+\beta)(1+\phi\epsilon)$	$(1+\alpha)^2(1+\beta)^2(1+\epsilon)^4$	
Ab	$(1+\alpha)(1+\beta)(1+\phi\epsilon+1)$	$(1+\alpha)$	$(1+\alpha)^2(1+\beta)(1+\epsilon)^2$	$(1+\alpha)^2$

Table S1: Comparison of the parametrization of genetic incompatibilities between the model of Schumer et al. (2015) (top) and our model (bottom). The derived alleles follow the same nomenclature. The ancestral alleles, which we called “a” and “b” are represented by “x” in the Schumer model.

S 1 Model: Comparison with Schumer et al. (2015)

We model classical DMIs (Orr and Turelli, 2001) with a small direct selective advantage of the derived type. We illustrate here how our implementation is similar to the case of “adaptive DMIs” from Schumer et al. (2015). Both implementations are presented next to each other in Table S1 and illustrated in Figure S6 for comparison.

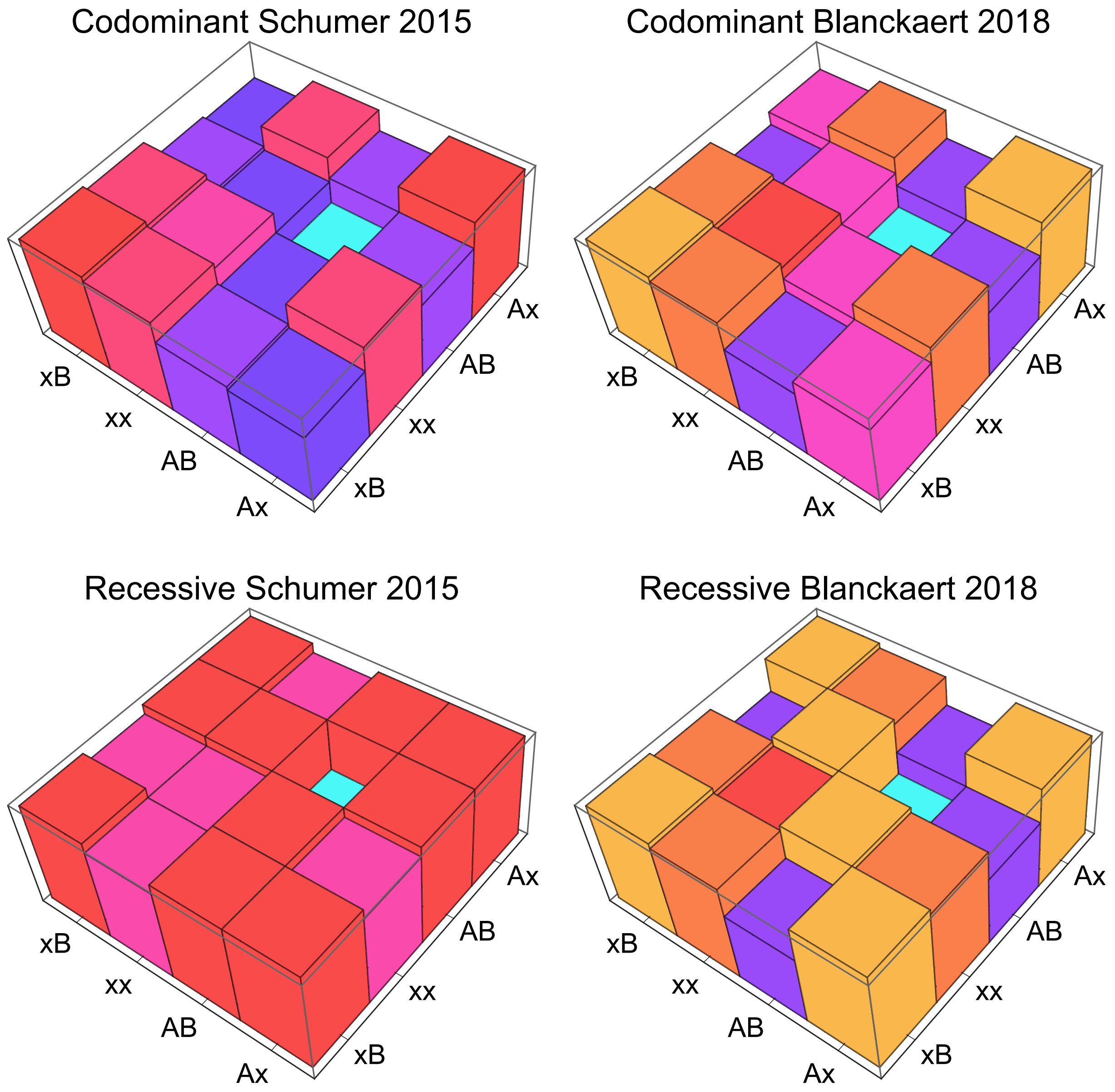

Figure S6: Illustration of the fitness landscapes for a single DMI in our model and an "adaptive" DMI from Schumer et al. (2015), Fig. S1. To facilitate the comparison, we use the notation of Schumer et al. (2015), as presented in Table S1. Parametrization was chosen such that the fitness differences between parental and ancestral genotypes and the strength of the incompatibility for the homozygote incompatible ABAB are the same.

S 2 Resolution of a single DMI

S 2.1 Relative proportion of A and B alleles, not initial frequency, determines evolutionary outcome

As illustrated in Figure 3 in the main manuscript, the fate of the DMI is strongly dependent on the initial frequency of the two parental genotypes. Here, we demonstrate that the relative proportion of the parental genotypes, not their initial frequency, determines the evolutionary outcome. This confirms the intuition that frequency-dependent selection based on the relative proportion of A and B alleles in the population drives the allele-frequency dynamics. In Figure S7, we compare the fate of the DMIs in the original setting (i.e., starting with 100% parental genotypes), and when both parental genotypes are introduced at the same initial i_q frequency (thus representing a relative proportion of 50% A and B alleles) and the ancestral haplotype at frequency $1 - 2i_q$.

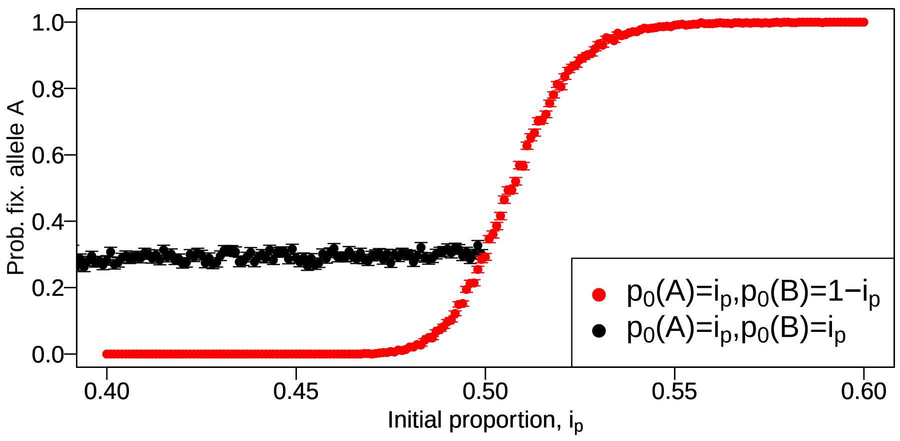

Figure S7: Probability of fixation of allele A following formation of the hybrid population. In one case (red dots), haplotype Ab is introduced at proportion i_p and aB at proportion $1 - i_p$. To demonstrate that the frequency-dependent effect is dependent on the relative proportion of the A and B allele, in the other case (black dots), both haplotypes Ab and aB are introduced at proportion i_q and the ancestral haplotype ab is introduced at proportion $1 - 2i_q$.

S 2.2 Loss of adapted alleles from the hybrid population

Fixation of the ancestral haplotype is a likely event, especially for neutral DMIs and symmetric proportions of starting genotypes. That is because the expression of the incompatibility reduces the marginal fitness of both derived alleles, which creates a hybrid cost and therefore indirectly generates positive selection for the ancestral haplotype. It is important to note that this positive selection is frequency-dependent, but it is not dependent of the frequency of the haplotype ab itself but on the relative frequency of the incompatible alleles A and B (see above).

The dominance pattern has an interesting effect on the probability of fixing the ancestral haplotype. Around a symmetric contact, the probability of fixation of the ancestral haplotype is higher for a codominant DMI than for a recessive DMI. If the contact is asymmetric, the opposite is observed: a recessive DMI is more likely to lead to fixation of the ancestral haplotype. That is because in the case of a symmetric contact, both derived alleles are driven to very low frequencies by the indirect frequency-dependent selection described above (as it is a function of $p_A p_B$), which

leaves them sensitive to the effect of genetic drift once epistasis becomes negligibly weak. In the recessive case, selection becomes weak while the derived alleles are still at higher frequency (as selection strength is a function of $p_A^2 p_B$ or $p_A p_B^2$), and therefore the probability to maintain one of the derived alleles becomes larger. Conversely, in the case of asymmetric contact, the more frequent derived allele is more protected from extinction because frequency-dependent selection ceases when the less frequent allele becomes rare, decreasing the chance to recover the ancestral haplotype. This behavior is specific to the codominant case, as recessive derived alleles remain at a higher frequency in the first place. In both cases, direct selection (if $\alpha, \beta \ll \|\epsilon\|$) plays a role mainly in the second phase: only once the (first) purging phase is finished, directional selection helps the surviving derived allele to reestablish itself in the population.

The time to resolve the DMI is another crucial factor that determines the evolutionary outcome. That is because the longer the derived alleles remain at low frequencies, the longer they are susceptible to drift, Figure S9. DMI resolution is especially slow for recessive DMIs, which counterbalances the effect described above, i.e. the frequency of an incompatible recessive allele remains higher than the frequency of an incompatible codominant allele during the purging phase. This leads to the following dichotomy: on the one hand, a recessive allele remains at a higher frequency but spends much more time (see Fig. S8) at this frequency: its exposure to drift is relatively less but over a longer period of time. On the other hand, codominant incompatible alleles face an opposite scenario: they are more exposed to drift but for a shorter time, which leads to the difference between the symmetric and asymmetric case. Indeed, as illustrated in an example in Fig. S8, the advantage of the masking effect lasts around ≈ 50 generations in the asymmetric case whereas it lasts ≈ 4700 generations in the symmetric case.

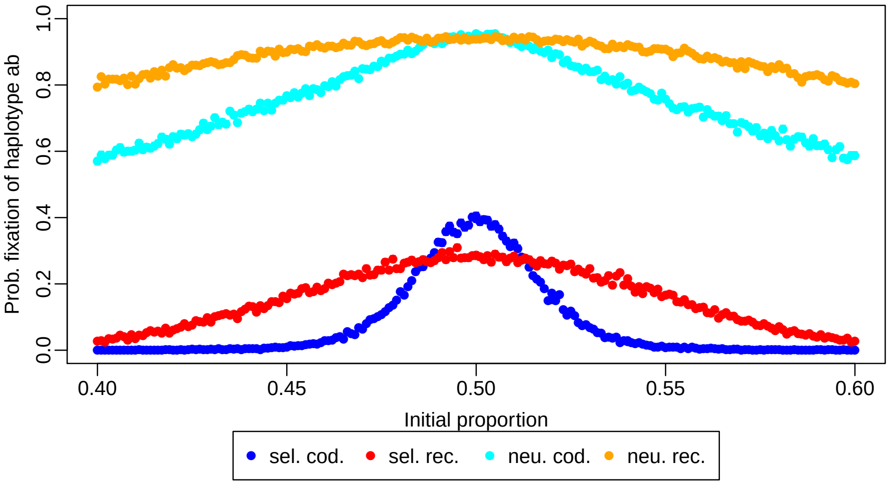

(a) Fixation probability of the ancestral haplotype

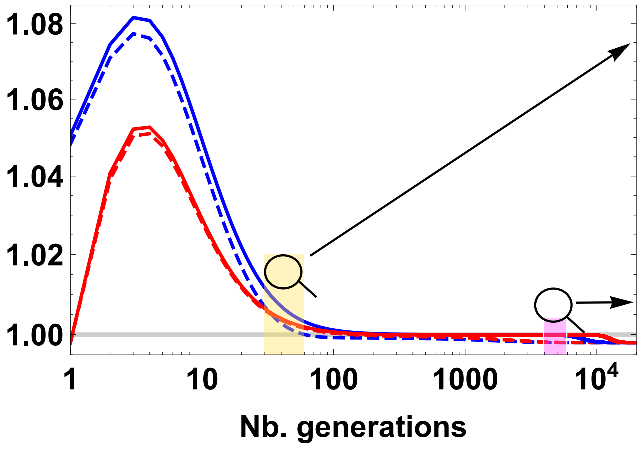

(b) Marginal fitness of the ancestral haplotype in the deterministic case.

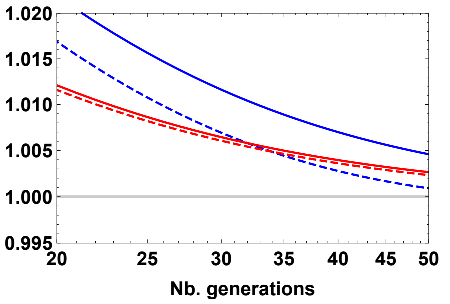

(c) Order inversion for the symmetric case

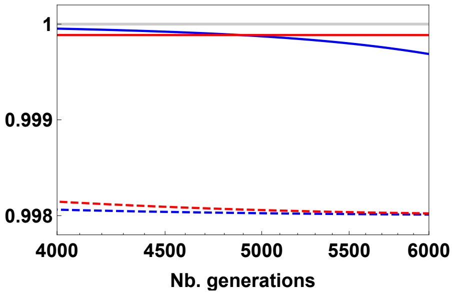

(d) Order inversion for the asymmetric case

Figure S8: Dominance affects the probability of recovering the ancestral haplotype in a hybrid population. Panel (a) shows the probability of recovering the ancestral haplotype ab in an isolated hybrid population for the two dominance schemes both for neutral ($\alpha = \beta = 0$) and slightly advantageous mutations ($\alpha = \beta = 0.001$). Panel (b) tracks the marginal fitness of the ab haplotype in the deterministic model, for an (almost) symmetric contact (solid lines) and an asymmetric proportion of the parental genomes (dashed lines). Blue and red dots correspond to the data from panel (a). Due to the masking effect of recessivity, the marginal fitness of ab is always lower in the recessive case than in the codominant case. Panels (c) and (d) correspond to subsets of panel (b) and illustrate at which point the order of probabilities is reversed for symmetric (panel (d)) and asymmetric contact (panel (c)). Order inversion means that from this time point onwards, masking no longer provides an advantage to the derived alleles. The longer the derived alleles are masked by recessivity, the more likely the ancestral haplotype will fix while both derived alleles are present at an equally low frequency, and therefore susceptible to be lost through drift. Other parameters used are: $N = 5000, \epsilon = -0.2$ and $\alpha = \beta = 0.001$ for panels (b-d). In panel (a), each data point is obtained from 2000 simulations.

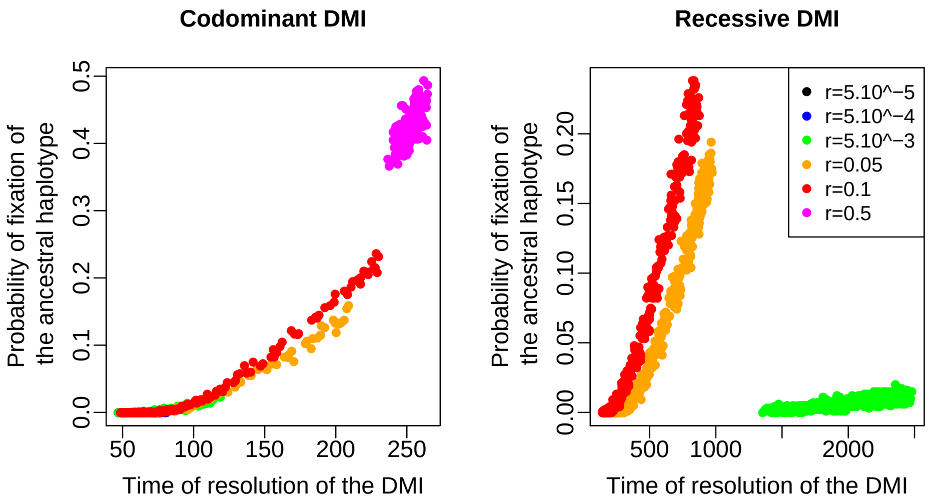

Figure S9: Probability of recovering the ancestral haplotype increases with the time it takes to resolve the DMIs. Colors represent different recombination rates between the A and B loci. Each dot is the result of 1000 simulations. Parameters used are: $\alpha = \beta = 0.001$ and $N = 5000$.

S 3 Resolution of two DMIs

S 3.1 Recessive DMIs

The probability of hybrid speciation as a function of recombination is discussed for codominant DMIs in the main manuscript. Hybrid speciation for recessive DMIs is qualitatively similar, as illustrated by Figure S10. Although hybrid speciation is usually rare, we observe a high probability of hybrid speciation for two specific linkage architectures: the "Adjacent ABAB" and "Crossed AABB" architectures. Also the "Nested ABAB" and "Nested AABB" architectures show qualitatively the same behavior between recessive and codominant DMIs. However, there is one major difference for two linkage architectures, the "Adjacent ABBA" and the "Crossed ABBA" architectures. For these two architectures we observe two local maxima of the hybrid speciation probability. Whereas the local maximum at large recombination rate is similar to that observed for codominant DMIs, the second one at low recombination rate is unique to recessive DMIs. Below, we explain where this second maximum comes from by detailing the outcome of the secondary contact across recombination rate regimes.

- With no recombination, there are only two possible outcomes: fixation of one of the two parental haplotypes. Here, either outcome has a 50% chance of happening if the genotypes are introduced at the same proportion and their fitness is identical. This is indeed what we observe in Figures S11 and S12 panels a) to d). (Note that this is not observable in the last two panels because the population size ($N * r \geq 1$) is too large and therefore recombination is not rare enough.)
- When each locus is located on a different chromosome ($r = 0.5$), all possible haplotypes are generated in the F2 generation. The final outcome corresponds to the fixation of the fittest haplotypes at equal probabilities: the "pure" parental haplotypes and the two hybrid haplotypes, which all have a fitness $(1 + s)^4$ (as $\alpha_k = \beta_k = s$) when fixed in the population. This is visible in Figure S11 (panel e-f) or Figure S12. When the population size is large, the role of drift is reduced, and the sorting of the DMIs can happen without losing more than two derived alleles.
- Between these two extremes, we can consider two intermediate regimes: one for rare recombination, and one that also described in the main manuscript, in which there is around one recombination event per individual per generation.
  - In the case of one recombination event per generation (roughly $r \approx 1/3$), the outcome of the hybrid breakdown in the F2 generation is crucial. Among the six recombinant haplotypes (see Table 2 in the main manuscript), only two are relatively epistasis-free (highlighted in yellow in the table): $A_1b_1b_2a_2$ and $a_1b_1b_2A_2$ for the "Adjacent ABBA" and "Crossed ABBA" architecture. This creates a marginal fitness advantage for both A_1 and A_2 compared to B_1 and B_2. Both DMIs are thus resolved towards the A allele in the deterministic case, which leads to the fixation of the parental haplotype $A_1b_1b_2A_2$, as illustrated in Fig. S11 panel d) to f) and Fig. S12. The strength of this effect depends on the number of "epistasis-free" haplotypes generated per generation and therefore occurs for a larger range of recombination rates for larger population sizes. Thus, the same mechanism that creates a high probability for hybrid speciation in the "Adjacent ABAB" and "Crossed ABAB" architectures creates a low-probability valley in the "Adjacent ABBA" and "Crossed ABBA" architectures.
  - The second peak of the hybrid speciation probability, which is unique to the recessive model, occurs for low recombination rates. Here, recombination is rare and therefore the "epistasis-free" haplotypes discussed above are not always generated. The DMIs can therefore sometimes be resolved towards different parental origins, which leads to hybrid speciation. This happens mainly in large populations because smaller

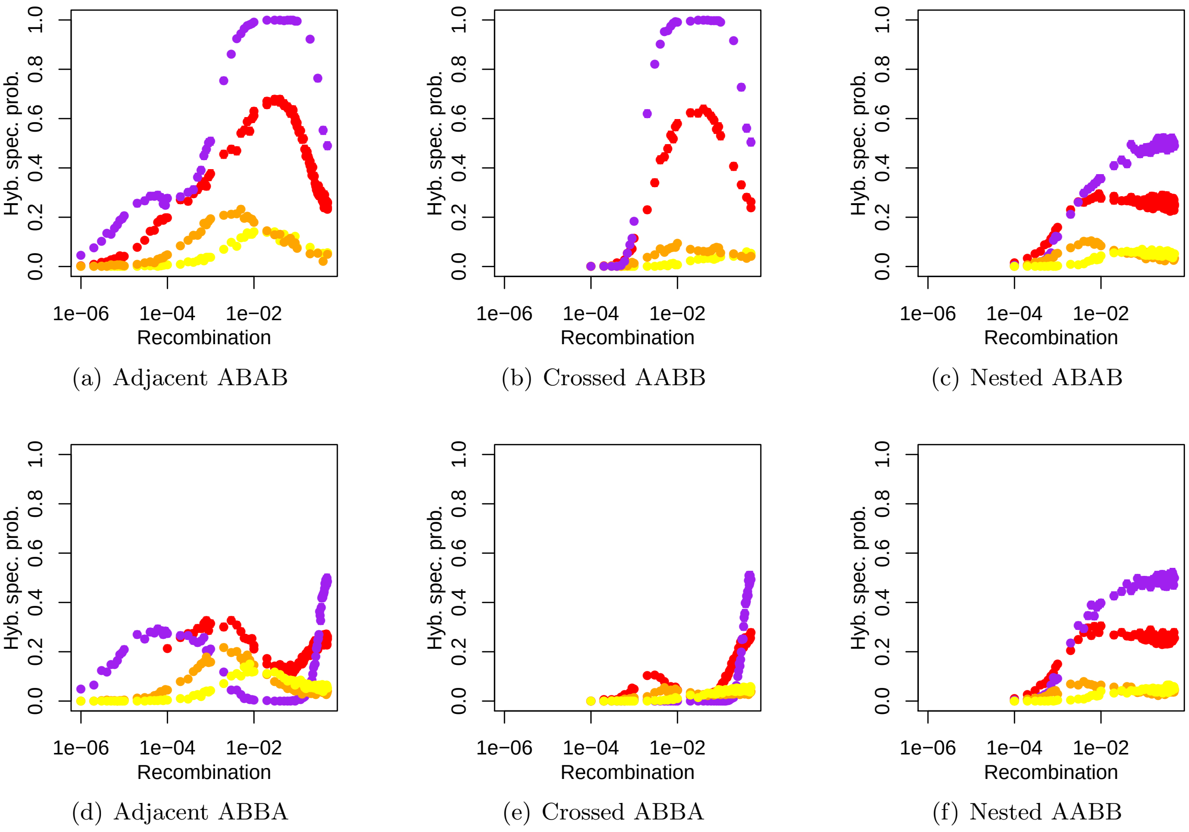

(a) Adjacent ABAB (b) Crossed AABB (c) Nested ABAB

(d) Adjacent ABBA (e) Crossed ABBA (f) Nested AABB

Figure S10: Hybrid speciation probability for recessive DMIs as a function of recombination for the six possible linkage architectures. We represent this probability for four different population sizes: $N = 50$ in yellow, $N = 500$ in orange, $N = 5000$ in red and $N = 50000$ in purple. Other parameters are $\alpha_k = \beta_j = 0.001, \epsilon = -0.2$ and $i_p = 0.5$ (i.e. the contribution of both parental populations is symmetric). This figure corresponds to Fig. 5 for codominant DMIs.

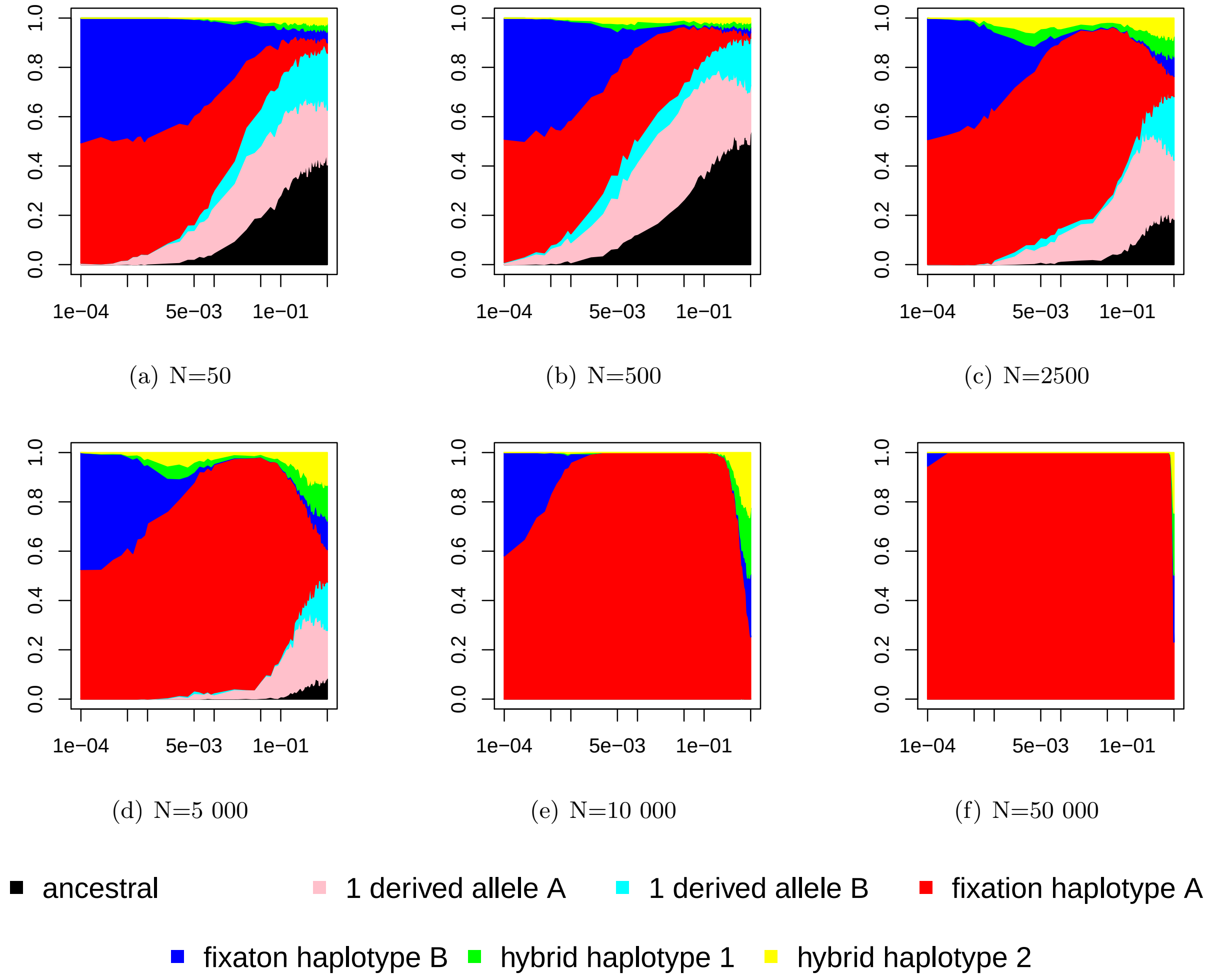

Figure S11: Proportions of possible evolutionary outcomes for the "Crossed ABBA" architecture. The x-axis corresponds to the recombination rate and each panel shows a different population size. To better illustrate the underlying mechanisms, we represent both hybrid haplotypes separately with $A_1B_2b_1a_2$ in green and $a_1b_2B_1A_2$ in yellow.

populations tend to be associated with loss of some of the diverged alleles even after the resolution of the DMI due to drift (as shown by the proportion of the pink and cyan color in the first row of Fig. S11 and Fig. S12). Codominant incompatibilities do not exhibit this behavior as the incompatibilities are expressed in any F1-cross. Therefore, we usually observe resolution of codominant DMIs towards one of the two parental haplotypes. Such resolution is rather rapid, leaving no time for the generation of the "epistasis-free" haplotypes in any meaningful quantity. Reciprocal sorting is close to impossible in this scenario.

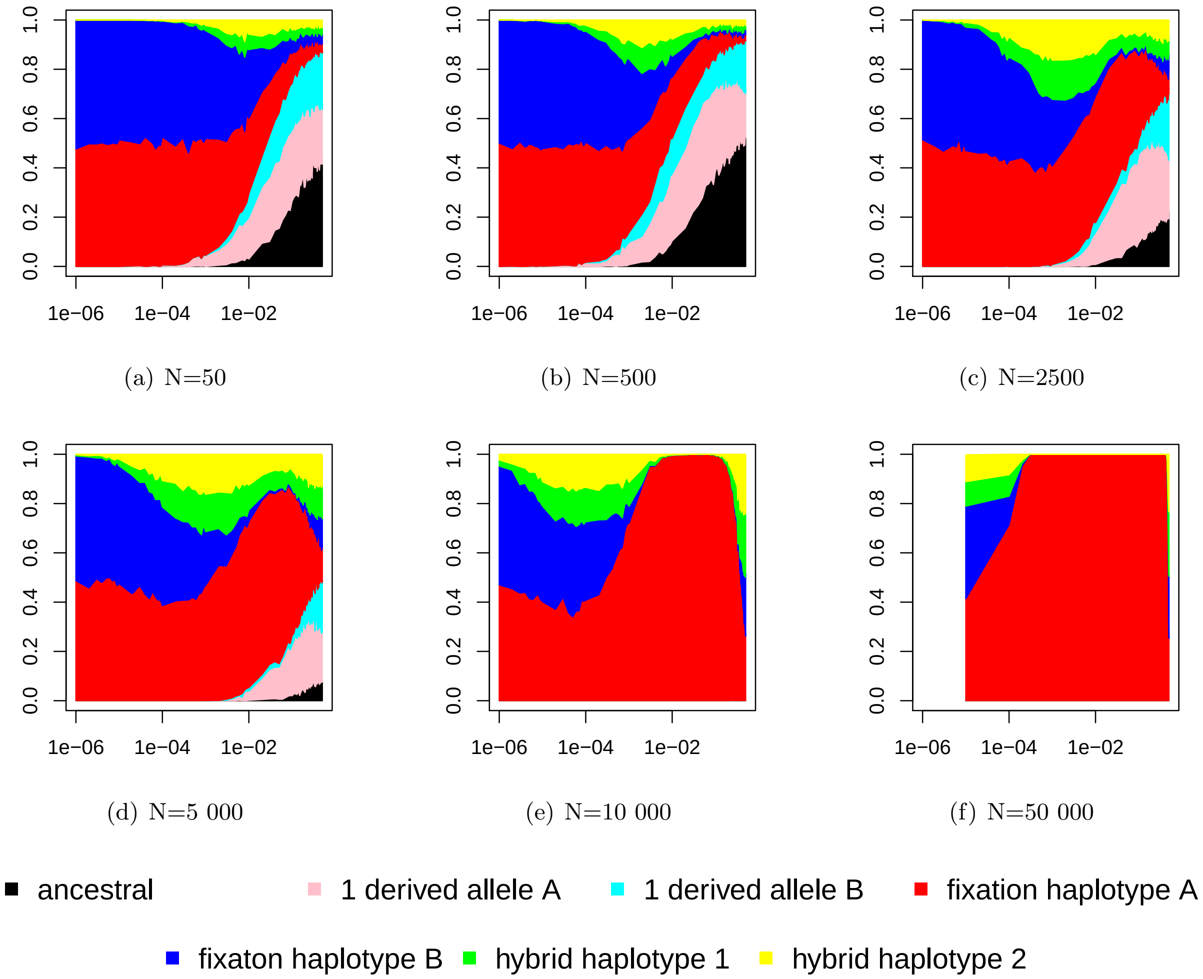

Figure S12: Proportions of possible evolutionary outcomes for the “Adjacent ABBA” architecture. The x-axis corresponds to the recombination rate and each panel shows a different population size. To better illustrate the underlying mechanisms, we represent both hybrid haplotypes separately with $A_1B_2b_1a_2$ in green and $a_1b_2B_1A_2$ in yellow.

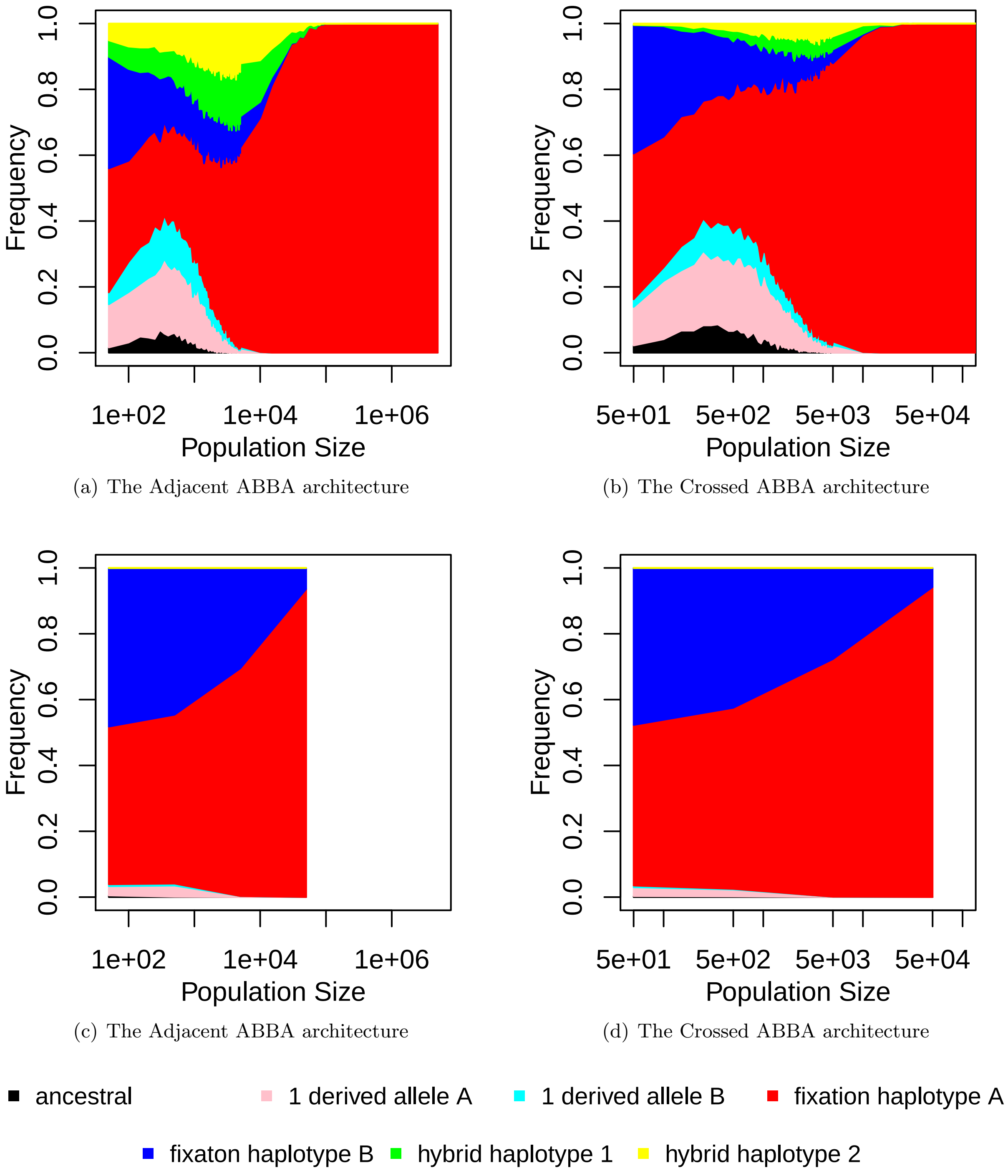

(a) The Adjacent ABBA architecture

(b) The Crossed ABBA architecture

(c) The Adjacent ABBA architecture

(d) The Crossed ABBA architecture

Figure S13: Hybrid speciation probability as a function of the population size for recessive (top) and codominant (bottom) DMIs. We focus on the two architectures that displayed a second local maximum of hybrid speciation probability for low recombination rate, here $r = 0.005$. Each color corresponds to a different evolutionary outcome.

S 3.2 Lethal incompatibilities

In the main manuscript, we consider DMIs that are strong but not lethal ($\epsilon = -0.2$). Lethal incompatibilities ($\epsilon = -0.99$; we did not use $\epsilon = -1$ to avoid potential computational issues when normalizing fitness values) correspond to a limiting case that is frequently investigated and biologically interesting. Figure S14 represents the hybrid speciation probability for all 6 linkage architectures, both for recessive and codominant DMIs. The outcome for codominant is utterly changed because hybrid speciation becomes impossible. That is because F1 hybrids do not survive, which prevents the formation of different F2 hybrids and thus reciprocal sorting. For recessive DMIs, the effect of DMIs is masked in the F1 generation, and upon formation of the F2 the most fit epistasis-free haplotypes compete, which makes the strength of epistasis almost irrelevant. The probability of hybrid speciation is thus very similar to the case investigated and illustrated in Fig. S10. Also with respect to the time to hybrid speciation (discussed in Subsection S 3.3), there is no apparent difference between recessive deleterious DMIs ($\epsilon = -0.2$) and recessive lethal ($\epsilon = -0.99$) DMIs.

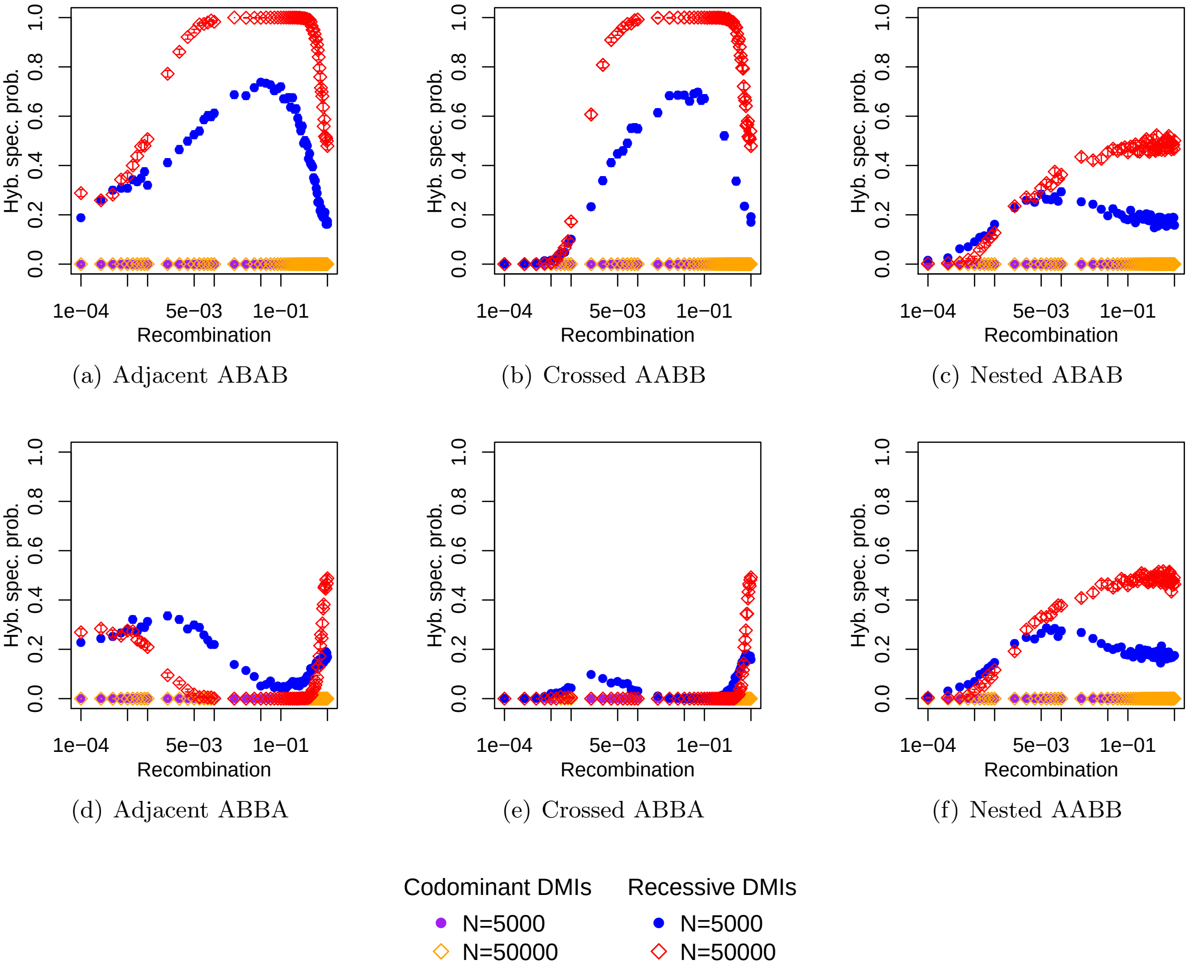

(a) Adjacent ABAB (b) Crossed AABB (c) Nested ABAB

(d) Adjacent ABBA (e) Crossed ABBA (f) Nested AABB

Figure S14: Hybrid speciation probability for (quasi-)lethal DMIs as a function of recombination. We consider both codominant and recessive DMIs and two population sizes. Hybrid speciation does not occur for codominant DMIs regardless of population size. For recessive DMIs, the hybrid speciation probability is qualitatively identical to the less deleterious case, see Fig. S10 with the blue dots here matching the red dots in Fig. S10 and the red diamonds the purple dots in Fig. S10. Parameters used are: $\alpha = \beta = 0.001$, $\epsilon = -0.99$.

S 3.3 Time to hybrid speciation

In this section, we focus on the time to hybrid speciation, which is the time to the long-term equilibrium of the dynamics conditioned on the outcome being reciprocal sorting. This time is strongly correlated with the population size and all observed values lie within one order of magnitude of N, from $0.5N$ for very large populations (purple dots in Fig. S15 and S16) to $5N$ for very small populations (orange and yellow dots). Surprisingly, hybrid speciation for codominant and recessives DMIs occurs in a rather similar time frame unless recombination rates are very low. This similarity is unexpected because of the striking differences in the time of the resolution of single DMIs discussed in the first part of the Results section of the main text (see Fig. 3 and S17). Intuitively, one may hypothesize that the similar hybrid speciation times occur because only quickly resolved recessive DMIs can lead to hybrid speciation. This is the opposite of what we observe (see Fig. S17): those simulations that result in hybrid speciation have on average a longer resolution time of the two DMIs as compared with an average simulation. A possible explanation for this pattern is that the strong linkage disequilibrium which is present in the hybrid population at the beginning has to be broken up for hybrid speciation to occur, and this is more likely to happen in more slowly resolving populations.

The longer resolution time (i.e., the time until the epistatic conflict is lost from the population) of recessive DMIs is compensated by the much shorter fixation time of the remaining derived alleles. That is because the derived alleles that are involved in recessive DMIs tend to remain at higher frequency during the purging phase (as discussed in Subsection S 2.2) and can therefore sweep much faster in the population once the DMIs have been purged. At low recombination rates, the time to hybrid speciation and the time resolution of the DMIs become indistinguishable for recessive DMIs (Fig. S17). Since epistasis is masked as long as both derived alleles are heterozygous when present together in the same genome, recessive incompatible alleles can remain in the population without suffering from epistasis in the (near) absence of recombination. Various non-incompatible haplotypes are segregating in the population and resolution of the DMIs and hybrid speciation happen at the same time. For example, if $A_1a_2b_1b_2$ and $a_1A_2B_1b_2$ are the two most frequent haplotypes, the resolution is driven by selection. If $A_1a_2b_1B_2$ and $a_1A_2B_1b_2$ are the most frequent haplotypes, then drift determines the outcome.

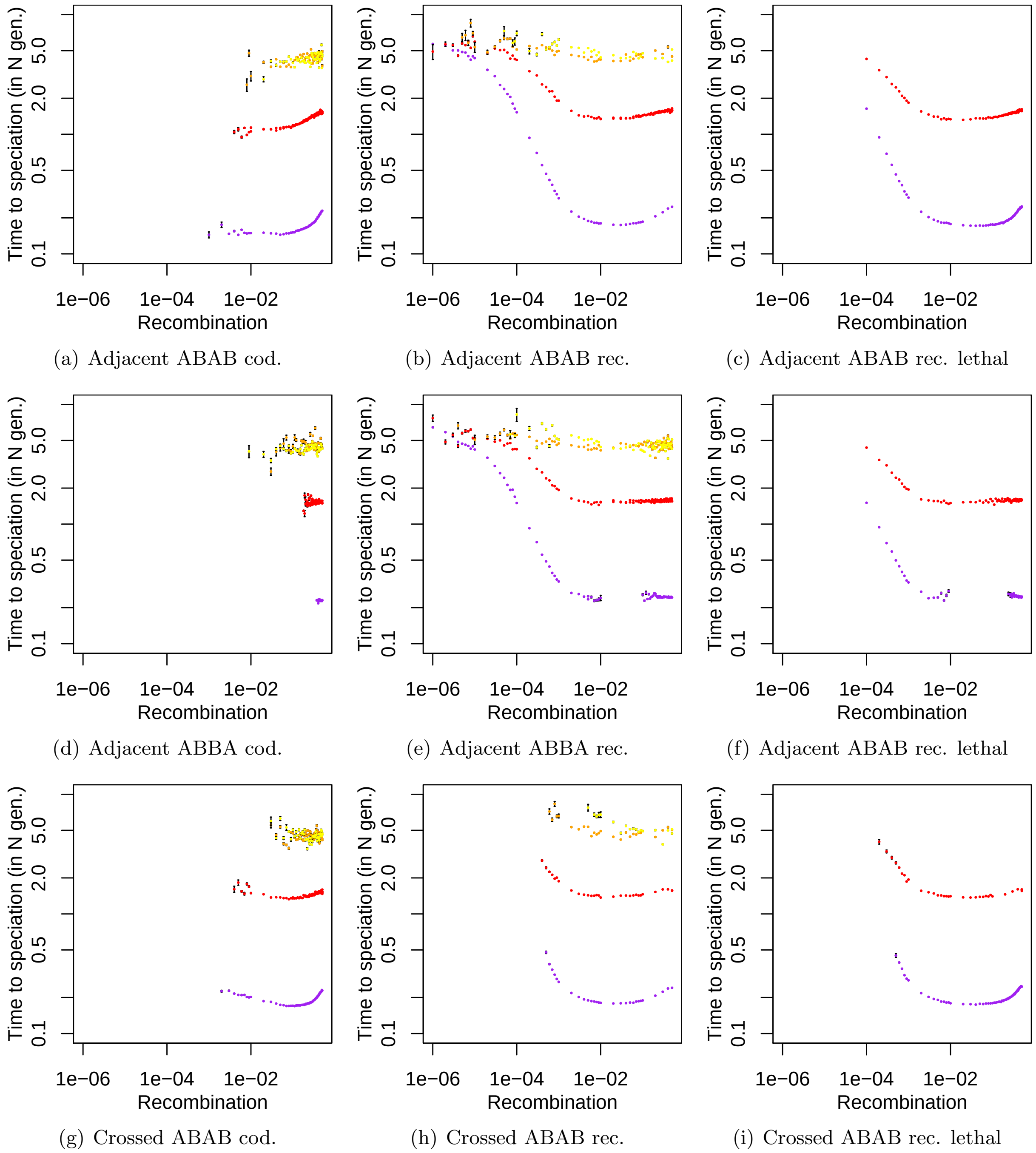

(a) Adjacent ABAB cod. (b) Adjacent ABAB rec. (c) Adjacent ABAB rec. lethal

(d) Adjacent ABBA cod. (e) Adjacent ABBA rec. (f) Adjacent ABAB rec. lethal

(g) Crossed ABAB cod. (h) Crossed ABAB rec. (i) Crossed ABAB rec. lethal

Figure S15: Time to hybrid speciation is relatively unaffected by the dominance of the epistatic interaction, the strength of the epistasis or the linkage architecture (here: Adjacent ABAB, Adjacent ABBA, Crossed ABAB). Each row of panels represents a different linkage architecture, for which the time to hybrid speciation is shown for codominant (left), recessive (middle), and recessive lethal DMIs (right). We show the average time to hybrid speciation, i.e. to fixation of one of the two hybrid haplotypes, scaled by the size of the population as a function of the recombination rate. Colors indicate different population sizes; purple: $N = 50000$, red: $N = 5000$, orange: $N = 500$ yellow: $N = 50$. Each set of simulations was obtained from 1000 simulations, of which those resulting in hybrid speciation were retained. We only show the time to hybridization if we observed at least 4 occurrences of hybrid speciation. Standard errors are represented by black bars.

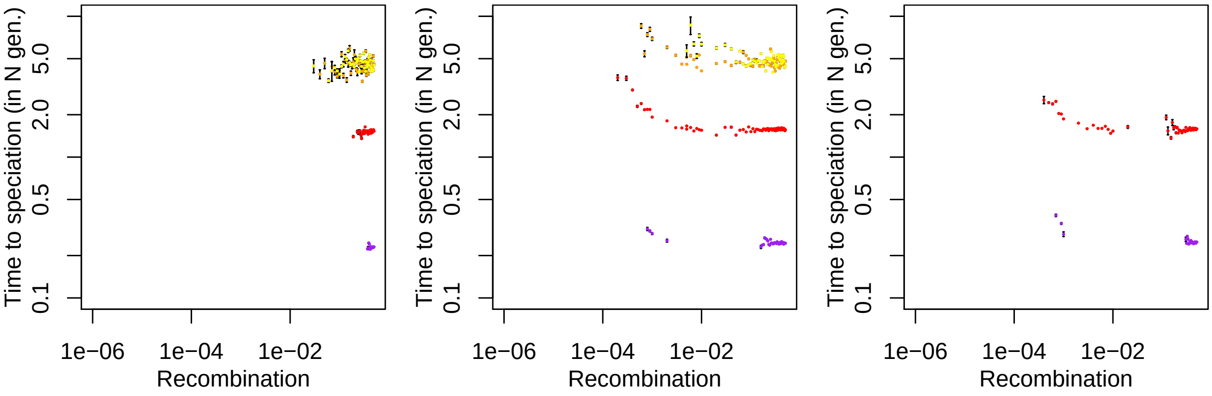

(a) Crossed ABBA cod. (b) Crossed ABBA rec. (c) Crossed ABAB rec. lethal

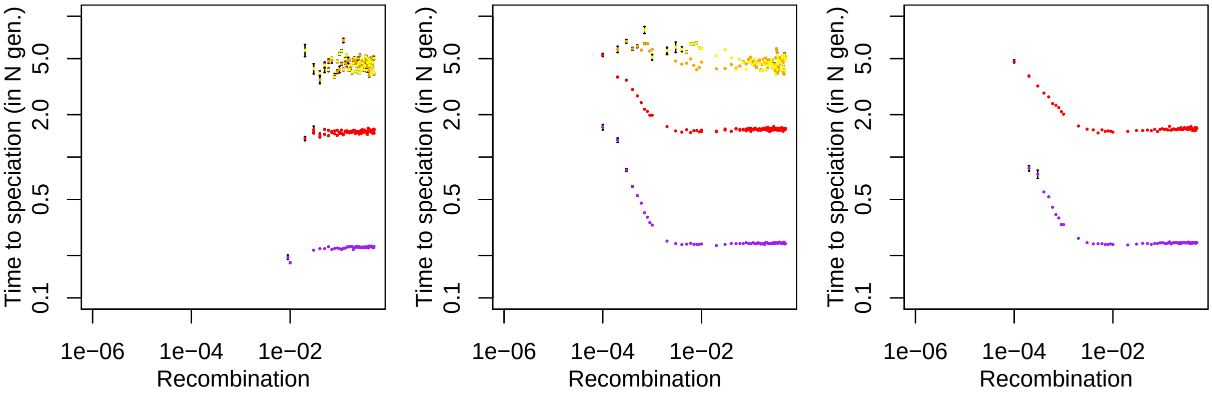

(d) Nested ABAB cod. (e) Nested ABAB rec. (f) Nested ABAB rec. lethal

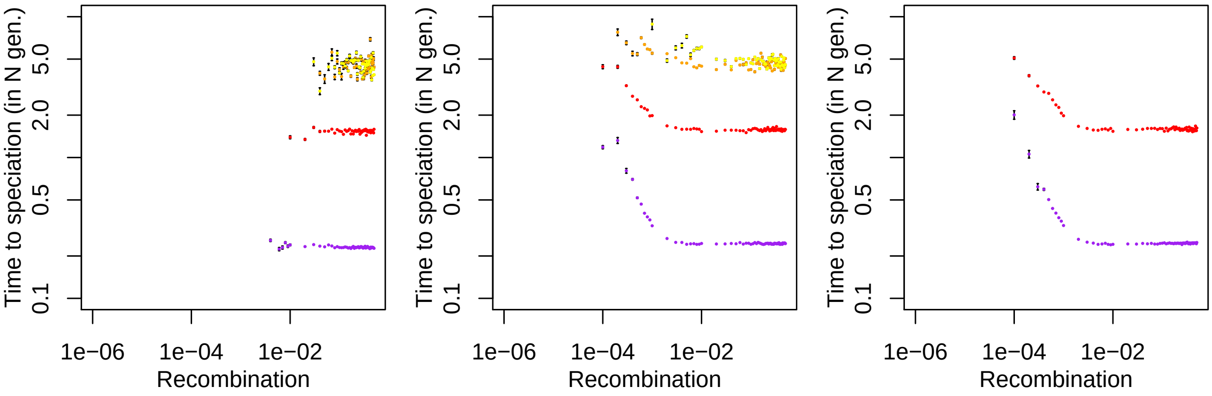

(g) Nested AABB cod. (h) Nested AABB rec. (i) Nested ABAB rec. lethal

Figure S16: Time to hybrid speciation is relatively unaffected by the dominance of the epistatic interaction, the strength of the epistasis or the linkage architecture (here: Adjacent ABAB, Adjacent ABBA, Crossed ABAB). Each row of panels represents a different linkage architecture, for which the time to hybrid speciation is shown for codominant (left), recessive (middle), and recessive lethal DMIs (right). We show the average time to hybrid speciation, i.e. to fixation of one of the two hybrid haplotypes, scaled by the size of the population as a function of the recombination rate. Colors indicate different population sizes; purple: $N = 50000$, red: $N = 5000$, orange: $N = 500$ yellow: $N = 50$. Each set of simulations was obtained from 1000 simulations, of which those resulting in hybrid speciation were retained. We only show the time to hybridization if we observed at least 4 occurrences of hybrid speciation. Standard errors are represented by black bars.

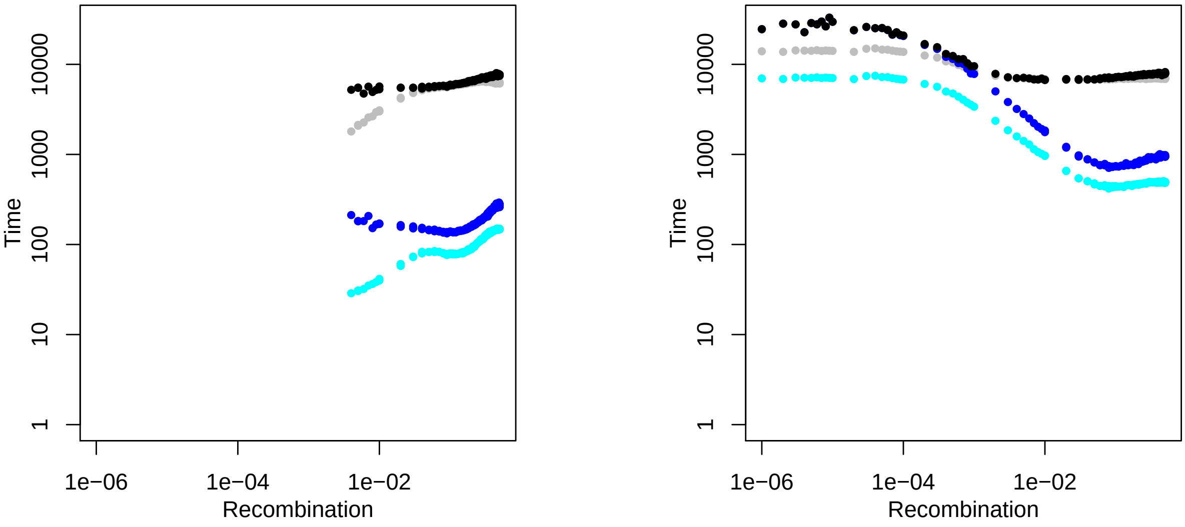

(a) Codominant DMIs (b) Recessive DMIs

Figure S17: The time to hybrid speciation is similar between codominant and recessive DMIs despite a much faster resolution of the two DMIs in the codominant case. We show the time to resolution of the two DMIs (blue and cyan) and of loss of all polymorphism (i.e., a haplotype has fixed; black and gray). We compare the time of fixation of a hybrid haplotype (black) to the average fixation time of a haplotype (gray). In addition, we compare the average time to resolution of both DMIs for all evolutionary outcomes (cyan) and conditioned on the occurrence of hybrid speciation (blue). The linkage architecture used here is “Adjacent ABAB”. Each parameter set was obtained from 1000 simulations. We only display the time to hybrid speciation if we observed at least 4 occurrences of hybrid speciation for the respective parameter combination.

S 4 Stability of the hybrid haplotype

We analyzed the local stability of fixation of the hybrid haplotype in the "Adjacent ABAB" linkage architecture. Although the deterministic system of 16 equations is not analytically solvable, fixation of the hybrid haplotype is an obvious equilibrium. We obtain

$$s < \min\left(\frac{1}{(\epsilon_1+1)^2} - 1, \frac{1}{(\epsilon_2+1)^2} - 1\right) \tag{S1}$$

as condition for local stability of fixation of the $a_1B_1A_2b_2$ and $A_1b_1a_2B_2$ haplotypes in the deterministic model. We assume that all derived alleles have the same fitness advantage s. This condition holds both for codominant and recessive DMIs because since the interacting allele is fixed, an invading mutation will suffer the same amount of epistasis in both models. This condition also holds for the "Crossed AABB" architecture for codominant and recessive DMIs.

References

H. Orr and M. Turelli. The evolution of postzygotic isolation: accumulating Dobzhansky-Muller incompatibilities. *Evolution*, 55(6):1085–1094, 2001.

M. Schumer, R. Cui, G. Rosenthal, and P. Andolfatto. Reproductive isolation of hybrid populations driven by genetic incompatibilities. *PLoS Genet*, 11(3):e1005041, 2015.
